# The determinants of health and health status of individuals in police custody in Australia: A scoping review

**DOI:** 10.1371/journal.pone.0338957

**Published:** 2025-12-30

**Authors:** Joshua F. Ginnane, Claudia Martin, Rebecca J. Winter, Mark Stoové, Shelley J. Walker

**Affiliations:** 1 Justice Health, Burnet Institute, Melbourne, Australia; 2 School of Global Public Health, New York University, New York, United States of America; 3 School of Public Health and Preventive Medicine, Monash University, Melbourne, Australia; 4 Department of Gastroenterology, St. Vincent’s Hospital Melbourne, Australia; 5 Australian Research Centre in Sex, Health and Society, La Trobe University, Melbourne, Australia; 6 National Drug Research Institute, Curtin University, Melbourne, Australia; Kore University of Enna: Universita degli Studi di Enna 'Kore', ITALY

## Abstract

**Background:**

In Australia, the health of people in police custody facilities and the conditions of their detention have been the focus of repeated scrutiny and investigations since the Royal Commission into Aboriginal Deaths in Custody was published in 1991. However, there remains a lack of comprehensive, consolidated evidence on the health needs of this population. To address this gap, we conducted a scoping review to identify and describe the available information on their health status and determinants of health.

**Methods:**

We followed the JBI methodology for scoping reviews, including searching databases of academic and grey literature, and hand searching websites, citations and review articles published since 2000. Final searches were completed on February 3, 2025. Study characteristics, determinants of health, and health conditions were extracted from included sources, and analyzed by jurisdiction, date, and document type.

**Results:**

We identified 172 relevant information sources, including 141 grey literature documents and 31 academic publications, published between 2000 and 2024 from every state and territory in Australia. Sources most frequently used data from New South Wales, Western Australia, and Queensland. More than half (57%) of sources used data from the Drug Use Monitoring in Australia (DUMA) program. Individual (age, gender, Indigenous status) and mental health and drug use characteristics were the most frequently reported data. Other important health determinants such as tobacco use, diet, exercise, stress, exposure to violence, and environmental conditions of police custody were largely underreported. No general health status or burden of illness studies were identified.

**Conclusions:**

Available information on the health of people in police custody in Australia is fragmented, mostly drawn from a single program, and focused primarily on mental health and substance use, while data on physical health and broader health determinants are limited. Enhancing systems that routinely monitor and transparently report health priorities for police custody are needed to improve health service provision and support environments that promote detainee health.

## Introduction

In Australia, police custody facilities–also referred to as police cells, watch houses, or police lock-ups –are primarily for the short-term detention of people after arrest and whilst awaiting a court decision or transfer to a more permanent place of detention, such as prison or a juvenile justice facility. The operation and resourcing of police custody facilities in Australia are the responsibility of the police force in each state and territory. The health and wellbeing of people held in police custody facilities in Australia has received repeated scrutiny, especially since the Royal Commission into Aboriginal Deaths in Custody (RCIADIC) in 1991, which found some custodial authorities did not honor their legal duty of care, and in some cases, deaths in custody were causally linked to failures to provide adequate care [1]. For decades, regulatory and review institutes in Australian states and territories (including the Australian Federal Police and Commonwealth Ombudsman [2], the Commonwealth and Law Enforcement Ombudsman [3] the Queensland Crime and Corruption Commission [4], and the Victorian Ombudsman and Office of Police Integrity [5,6]) have repeatedly raised concerns about the conditions of police custody.

In contrast to the publicly available information about people entering and leaving prisons in Australia, including routinely collected data on their demographics, socio-economic circumstances, and health status from the Australian Bureau of Statistics [7] and the Australian Institute of Health and Welfare (AIHW) [8], little is known about people in police custody facilities. The Australian Institute of Criminology publishes some data on police detainees through the National Deaths in Custody Program (NDICP) which reports the number and nature of deaths occurring in prisons, police custody, and youth detention each year [9], and the Drug Use Monitoring in Australia (DUMA) program which collects quarterly data from people in police custody to examine the relationship between drugs and crime, and monitor local drug markets [10]. However, these systems have methodological and reporting limitations [10,11] which means comprehensive data about the health and wellbeing needs of people in police custody facilities are sparse. Unfortunately, national Australian data on the number of people who transit through police custody each year is not publicly available. As colleagues and others have argued previously [12,13], this lack of contemporary, reliable and publicly available data about the health issues of people in police custody facilities hinders advocacy efforts and the development of targeted strategies and interventions to address their needs.

Wardrop and colleagues [14,15] conducted two global scoping reviews of relevance to the health of people held in police custody facilities in Australia. One review examined academic literature on healthcare infrastructure and processes in police custody facilities [15] and the other mapped the health issues and care outcomes of individuals brought into emergency departments by police, (which included people transferred from police custody facilities) [14]. However, both studies were global and included only limited Australian evidence, and neither reported information on determinants of health. Our objective is to address these gaps by identifying and describing the available information on the health status and determinants of health of people in police custody facilities in Australia.

### Review questions

Five review questions guided the design and execution of this scoping review: 1) What information on the determinants of health and health status of people in police custody facilities in Australia have been reported in academic or grey literature since 2000?; 2) How has this data been collected and published?; 3) What are the key characteristics of the available information (e.g., year and regularity of publication, type of data, collection methods)?; 4) How does the published information vary by jurisdiction and over time?; and 5) What gaps in the available information can be deduced by the identification and analysis of the above information sources?

### Inclusion criteria

#### Population.

This review focused exclusively on people held in police custody facilities in Australia. Information sources reporting exclusively on other types of individuals associated with these settings, such as custodial or health staff, were excluded. Where information sources reported on both the health of people detained and the health of other individuals such as staff, only the data relevant to people detained were included.

#### Concept.

This review considered information sources that included data on either the health status or determinants of health of people in police custody facilities. This review adopted the AIHW definition and framework of the determinants of health to categorize and select relevant determinants of interest [16]. The focus of our review was on a broad range of determinants of health including those that pertain to individual health and socio-demographics (prior to or while in police custody), and the environmental conditions of police custody facilities (Table 1).

**Table 1:** Determinants of health under investigation.

Concept component	Categories	Timing of interest
Determinants of health: Individual characteristics	• Age • Gender • Indigenous status • Ethnicity • Country of birth • Migration status • Refugee status	While in police custody facilities
Determinants of health: Structural	• Discrimination or racism	Prior to entering police custody facilities or while in these facilities
Determinants of health: Socioeconomic	• Employment status • Housing status • Level of education • Income • Previous incarceration • Neighborhood, suburb, or local government area of detainee	Prior to entering police custody facilities
Determinants of health: Health knowledge	• Health literacy	Prior to entering police custody facilities or while in these facilities
Determinants of health: Health Behaviors	• Tobacco product use • Alcohol use • Level of physical activity • Dietary habits • Illicit drug use • Sexual practices • Sleep patterns	Prior to entering police custody facilities or while in these facilities
Determinants of health: Psychosocial	• Stress • Isolation or loneliness	Prior to entering police custody facilities or while in these facilities
Determinants of health: Safety	• Level of risk taking • Experience of violence • Exposure to occupational risks	Prior to entering police custody facilities or while in these facilities
Determinants of health: Environmental	• Level of occupancy or overcrowding • Air quality or access to fresh air • Water quality • Heat/ cold • Sanitation • Access to healthcare • Duration of police detention	While in police custody facilities
Determinants of health: Biological	• Body weight • Blood pressure • Blood cholesterol • Blood glucose	While in police custody facilities

In addition, this review considered information sources with descriptions of the health status of people in police custody facilities, including quantitative information, such as the incidence or prevalence of disease, and qualitative information such as descriptions of the experiences of health, disease or injury. Information about interventions or strategies to improve health was beyond the scope of this review. If information sources were identified that provided data both on health and health interventions, then only data pertaining to the health status of people in police custody facilities were included. Information sources exclusively describing the health status of people before or after their detention in police custody facilities were excluded.

#### Context.

The context of interest for our scoping review is police custody facilities that are operated and managed by the police in all eight states and territories of Australia. We excluded information sources that solely investigated other closed settings such as prisons, secure mental health facilities, immigration detention facilities or juvenile justice facilities, or that reported on individuals in police custody being transferred to other healthcare settings. Where information sources were identified that included information from multiple settings, only the relevant data pertaining to people held in police custody facilities were charted. For example, if an information source reported deaths in police custody and in prisons, and the data were disaggregated, only data pertaining to the deaths that had occurred in police custody facilities were included.

#### Types of information sources.

This review included academic literature (including conference abstracts), and grey literature published between 2000 and February 3, 2025. The year 2000 was chosen to balance comprehensiveness with a focus on more recent evidence and to provide a sufficient timeframe for assessing potential trends in publications over time. Only information sources which contained primary information were eligible for inclusion; reviews or summaries of previously published findings were excluded. Study protocols, news articles, practice guidelines, and policy statements were also excluded. Coroner’s reports about the circumstances surrounding an individual death in police custody were excluded for three reasons. First, coroner’s reports have already been examined elsewhere [17,18]. Second, coroner’s reports can be difficult or sometimes impossible to access in Australia [18]. Third, these reports are generally limited to establishing the cause of death, while important determinants of health such as Indigenous status, or drug and alcohol use are often omitted [18]. Inclusion and exclusion criteria are summarized in Table 2.

**Table 2:** Summary of inclusion and exclusion criteria.

	Included	Excluded
**Population**	• Sources on individuals in police custody facilities in Australia.	• Sources on individuals in police custody outside of Australia. • Sources on individuals who work in police custody facilities.
**Concept**	• Sources on the health or determinants of health of individuals in police custody facilities. • Sources on the health or determinants of health of individuals in police custody facilities in the context of an intervention.	• Sources exclusively on the use of health interventions in police custody, with no reporting of baseline health data. • Sources exclusively on health or determinants of health of individuals before or after police custody detention.
**Context**	• Sources on individuals in police custody facilities, also known as police cells, the lock-up, or watchhouses.	• Sources on individuals in other custodial settings such as prisons, or secure medical facilities. • Sources on individuals transferred from police custody to healthcare facilities. • Sources on individuals detained by police under conditions other than police custody facilities (e.g., under arrest in the community, in police transport)
**Types of sources**	• Research studies (including conference abstracts) containing primary data. • Government reports containing primary data. • Non-governmental organization reports containing primary data. • Year of publication during or after 2000.	• Sources summarizing existing data. • Study protocols. • Reports or investigations into the circumstances surrounding an individual death in custody (e.g., coroner reports). • News articles. • Practice guidelines. • Policy statements. • Sources published before 2000.

## Methods

This review was conducted in accordance with the JBI methodology [19], and the findings were reported in accordance with the Preferred Reporting Items for Systematic reviews and Meta-Analyses (PRISMA) extension for scoping reviews [20]. The objectives, research questions, inclusion criteria, and search methods were specified in advance and documented in a protocol registered on Open Science Framework in June 2024 [21]. During the screening process, additional categories of health data and health determinant data were identified. These were incorporated into the protocol and data collection tool in December 2024, with an amendment recorded on the registry page in February 2025. This amendment was registered after the screening of information sources had begun but prior to the commencement of data charting. As all included information sources were previously published, no ethics approval was required.

### Search strategy

The search strategy was designed with the assistance of a medical sciences librarian. Relevant academic and grey literature were identified using four methods. The first search method involved using systematic searches with combinations of key words and MeSH terms (where applicable) on MEDLINE (Ovid), Embase (Ovid), PsychINFO (Ovid), CINAHL Complete (EBSCO), Criminal Justice Abstracts (EBSCO), Scopus, Web of Science, and Criminal Justice Database (ProQuest).

The second search method involved systematic searches on five grey literature databases accessed through Informit: POLICY (Analysis and Policy Observatory); Australian Public Affairs Full Text; Aboriginal and Torres Strait Islander Health Bibliography; Australian Criminology Database; and AGIS Plus Text. See S1 Appendix for full search strategies used on academic and grey literature databases. As highlighted above, search results were limited to information sources published during or after the year 2000. All database searches were run on July 2, 2024, and repeated on February 3, 2025, before the screening process was finalized.

The third method involved hand searching for relevant information sources on the websites of organizations and agencies responsible for the health of police detainees, the physical conditions in police custody facilities, or complaints about police custody facilities in each state and territory in Australia. Searches used pre-specified key words that aligned with the review concept and context of interest. Hand searching was completed by the first author (JFG), between January 13 and February 10, 2025. For consistency and transparency, the search date, search terms, filters, number of results, and any relevant information sources identified were recorded in a log (S2 Appendix). If no search function was available, or if additional pages were browsed, these were also recorded in the same log.

The fourth method used to identify relevant information sources involved hand searching the references of included articles, and any systematic, scoping, or narrative review articles encountered during the screening process. Systematic, scoping, or narrative review articles were automatically excluded from our review but were logged in S3 Appendix for transparency. No authors or researchers outside of the authorship team were contacted as part of the search strategy.

### Information source selection

Search results from databases were uploaded to Covidence software and duplicates removed [22]. These information sources were screened independently by two authors (JFG & CM) at the title and abstract level, and then again at the full text level against the pre-specified inclusion criteria outlined in the protocol. Conflicts were resolved through discussion or referral to a third author (SW or RW). All excluded information sources that were reviewed at the full text level are recorded in S4 Appendix, with the primary reason for exclusion. All potentially eligible information sources identified by JFG during handsearching were screened by a second author (CM) prior to inclusion or exclusion.

### Data charting

A tool was developed in Microsoft Excel to streamline the data charting process (S5 Appendix) [23]. Variables chosen for extraction were aligned with the key research questions from the protocol, and included source characteristics, participant characteristics, research setting, and determinants of health or health status outcomes reported in the publication. The authorship team chose 39 determinants of health for inclusion in the data charting tool, which aligned with the AIHW determinants of health framework [16]. Where information sources provided any quantitative or qualitative information on the presence of diseases in people in police custody facilities, this information was captured and categorized according to the International Classification of Diseases 11th Revision (ICD-11) [24]. Both the individual illness, disease or condition (in free text), and the broader chapter that the illness corresponds to, were recorded for ease of analysis. Where sources contained information on deaths in police custody, and the cause of death could be mapped to ICD-11, the same charting process as for diseases was followed. Prior to commencing charting, the tool was piloted by two authors (JFG and CM) on 12 articles, including both academic and grey literature sources. Data charting was undertaken by the first author (JFG) and checked for accuracy by a second author (CM). Any conflicts were resolved through discussion or referral to a third author (SW). Critical appraisal of included information sources was not completed.

### Analysis

Results from the search strategy were recorded in a PRISMA flow diagram [20]. To answer the research questions, the data from included sources were analyzed according to publication type (academic or grey literature), date of publication, and the jurisdiction where the data was collected. Summary statistics on the characteristics, study design, and included determinants or health conditions were calculated.

To illustrate trends over time, the number of publications is presented in a line graph. The distribution of publications by jurisdiction is presented using stacked bar charts. Information on the determinants of health is summarized in tables and co-occurrence heatmap matrices, while information on health conditions is visualized in tables and in a treemap chart. For transparency and accessibility as a reference tool, all data extracted from included information sources are made available in the appendices.

## Results

**Fig 1:**
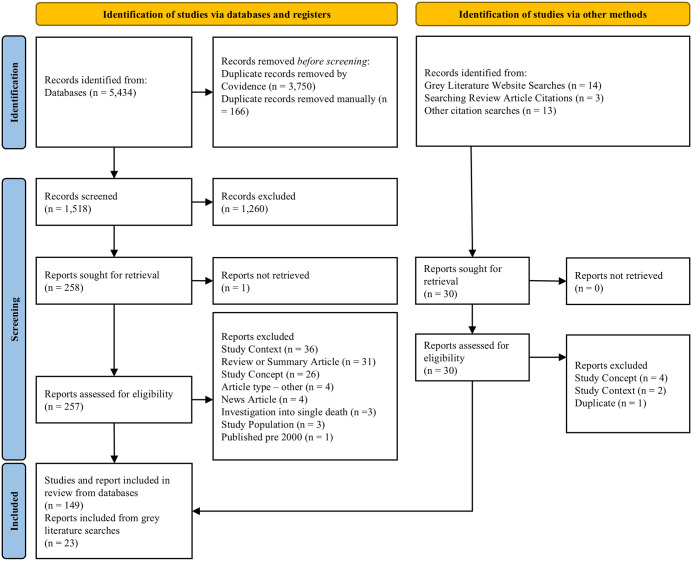
PRISMA Flow Diagram.

A total of 5,434 records were retrieved through database searches (Fig 1). Following duplicate removal, 1,518 titles and abstracts were reviewed against inclusion criteria including 143 new records which were identified when the search was repeated on February 3, 2025. Of 1,518 records, 258 progressed to full text evaluation. Of these, 257 records were retrieved, and 108 of these were excluded based on the eligibility criteria. Primary reasons for exclusion were the study context (n = 36), study concept (n = 26), or the information source being a summary or review of previously published data (n = 31) (S4 Appendix).

A total of 488 searches were executed across 51 webpages as part of the hand searching approach (three additional websites were identified in addition to the 48 pre-specified sites). An additional 163 subpages were browsed in place of or in addition to executing searches on these websites, depending on each site’s capabilities (S2 Appendix). This process captured 14 additional records, which were included in the review. During screening we flagged articles that included narrative reviews or summaries on the health or determinants of health of people in police custody facilities (S3 Appendix). Through searching the references of these articles, we found three additional information sources that met inclusion criteria, that had not been identified in the database or website searches. Searching the citations of included sources, we identified 13 further information sources, for a total of 30 identified through hand searching. Altogether, 172 information sources were included in this review (S6 Appendix). The number of information sources at each stage of screening is presented in Fig 1, and all extracted data are available in S7 Appendix where it can be searched or filtered by variable of interest.

### Characteristics of included information sources

**Fig 2:**
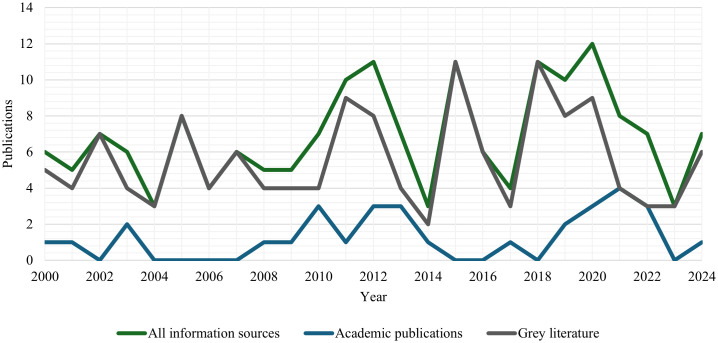
Information sources published per year. Each line represents the number of publications identified per year between 2000 and 2024 for all information sources, academic publications, and grey literature publications.

Of the 172 information sources, 141 were grey literature, and 31 were academic publications. Grey literature publications were overwhelmingly produced by government agencies (140 publications), with only one report independent of government meeting inclusion criteria [25]. Information sources were published between 2000 and 2024 (as per inclusion criteria). The number of academic publications ranged from zero to four per year, and the number of grey literature publications from two to 12 per year (Fig 2). The number of publications peaked in 2020 (n = 12) and were at their lowest in 2004, 2014, and 2023 (n = 3).

**Fig 3:**
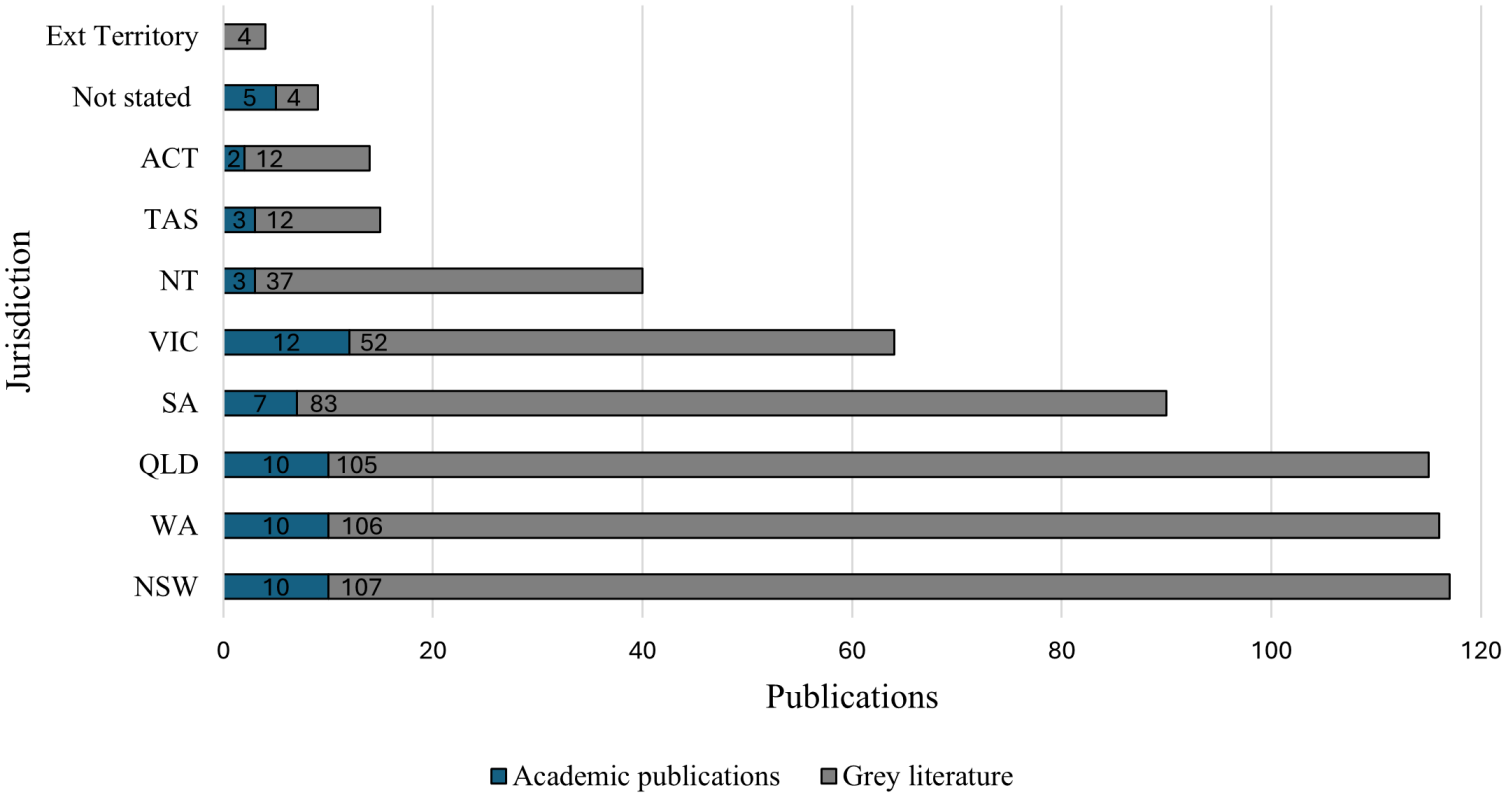
Academic and grey literature publications by jurisdiction of data collection. Each bar represents the number of information sources that collected data from that jurisdiction. Of the 172 included information sources, 115 included data from multiple jurisdictions. Abbreviations: ACT: Australian Capital Territory. Ext: External. NSW: New South Wales. NT: Northern Territory. QLD: Queensland. SA: South Australia. TAS: Tasmania. VIC: Victoria. WA: Western Australia.

Grey and academic literature were from every state and territory of Australia (Fig 3). Four reports were also identified that included data from External Australian Territories [26–29]. Most information sources (n = 115) included data from multiple states or territories. Information from New South Wales (NSW), Western Australia (WA), and Queensland were observed most frequently, with data from each of these jurisdictions observed in 117, 116, and 115 information sources respectively. More populous jurisdictions generally had the most published information sources, except for Victoria, which is the second most populous jurisdiction in Australia, but which had fewer information sources than WA, Queensland, and South Australia (SA). Data from Tasmania and the Australian Capital Territory (ACT) were observed in 15 or fewer information sources each. In nine information sources the state or territory of data collection was not specified. No clear trends were observed in the number of information sources published per year in individual jurisdictions (S8 Appendix).

Most information sources were grey literature and were most frequently government agency reports (n = 75), yearly or multi-year surveillance reports (n = 46), or government investigations (n = 19). Academic publications were most commonly retrospective analyses of routinely collected data that were not publicly available (n = 13) including sources such as the Victorian Institute of Forensic Medicine database, Clinical Forensics ACT, police records, historical DUMA data, and the Mental Health Disorders and Cognitive Disability dataset at UNSW. Sample sizes ranged from zero (where reports only included facility inspections) to 45,567 in one DUMA publication [30]. Most sources (n = 150) included data on both males and females and approximately one third of sources included data on juveniles (n = 58). More than half of the total information sources (57%) utilized data from the DUMA program, including almost 70% of information sources from NSW, WA, Queensland, and SA (Table 3).

**Table 3:** Number and proportion of information sources that used data from the Drug Use Monitoring in Australia (DUMA) program.

Jurisdiction	Total information sources	Number of information sources that used DUMA data (%)
New South Wales	117	84 (71.79%)
Western Australia	116	85 (73.28%)
Queensland	115	80 (69.57%)
South Australia	90	66 (73.33%)
Victoria	64	23 (35.94%)
Northern Territory	40	21 (52.50%)
Tasmania	15	0
Australian Capital Territory	14	0
Not Stated	9	4 (44.4%)
External Australian Territory	4	0

### Information on the determinants of health

Information on specific health determinants varied considerably (Table 4). Each information source included details of between one and 22 determinants of interest. Most sources provided information on individual characteristics of detainees such as gender, age, and Indigenous status, but very few provided information on the country of birth (n = 8) or ethnicity of detainees (n = 8). No information sources were identified that included information on the refugee or migration status of detainees. Some socioeconomic determinants were reported frequently, such as employment status (n = 60), educational attainment (n = 48), housing (n = 51), and previous history of imprisonment (n = 47). No sources reported information related to food security, and very few reported income (n = 2) or the residential neighborhood of people in police custody facilities (n = 3).

**Table 4:** Reported determinants of health.

Determinants of health: Individual characteristics	Number of information sources (%)
Gender	148 (86.05%)
Age	140 (81.40%)
Indigenous status	104 (60.47%)
Ethnicity	8 (4.65%)
Country of birth	8 (4.65%)
Migration status	0
Refugee status	0
**Determinants of health: Structural**	
Discrimination or racism	9 (5.23%)
**Determinants of health: Socioeconomic**	
Employment status	60 (34.88%)
Secure housing	51 (29.65%)
Education level	48 (27.91%)
Previous imprisonment	47 (27.33%)
Neighborhood, suburb, or local government area	3 (1.74%)
Income	2 (1.16%)
Food security	0
**Determinants of health: Health knowledge**	
Health literacy	0
**Determinants of health: Health behaviors**	
Illicit drug use	126 (73.26%)
Alcohol use	81 (46.51%)
Sexual practices	22 (12.79%)
Dietary habits	19 (11.05%)
Level of physical activity	15 (8.72%)
Tobacco use	7 (4.07%)
Sleep patterns	5 (2.91%)
**Determinants of health: Psychosocial**	
Stress or psychological distress	27 (15.70%)
Isolation or loneliness	12 (6.98%)
**Determinants of health: Safety**	
Level of risk taking	35 (20.35%)
Exposure to violence	28 (16.28%)
Exposure to occupational risks	0
**Determinants of health: Biological**	
Body weight	1 (0.58%)
Blood pressure	0
Blood cholesterol level	0
Blood glucose	0
**Determinants of health: Environmental**	
Access to healthcare in police custody	27 (15.70%)
Duration in police custody	22 (12.79%)
Sanitation	19 (11.05%)
Level of occupancy or overcrowding	15 (8.72%)
Air quality or access to fresh air	14 (8.14%)
Exposure to heat or cold	11 (6.40%)
Water quality	0

Health behaviors commonly associated with criminal behavior (the focus of most DUMA-derived literature) were reported most frequently: 126 information sources reported illicit substance use and 80 reported alcohol use. Sexual practices were reported in 22 information sources, almost exclusively (n = 21) in the context of reporting the number of people in police custody facilities engaged in sex work or exchanging sex for drugs. Sources reporting on diet (n = 19) and exercise patterns (n = 15) did so in the context of discussing the quality of food and access or lack of access to exercise spaces in police custody facilities. Very few (n = 7) information sources reported tobacco use. Relatively few information sources reported on psychological distress (n = 27) or isolation (n = 12) of people in police custody facilities. Of the environmental determinants reported, most were about access to health services while in police custody facilities (n = 27), followed by the duration of time spent in these facilities (n = 22).

When assessing the co-occurrence of health determinants (S9 Fig), clusters of determinants were aligned with the key areas of the DUMA program, including individual characteristics (age, gender, Indigenous status), socioeconomic factors (level of education, employment status, housing and previous imprisonment), and alcohol and other drug use. When comparing the co-occurrence of determinants between information sources that utilized DUMA data (S10 Fig) and sources that used other data (S11 Fig) differences were observed. First, information sources that utilized DUMA data did not report on the environmental conditions in police custody facilities. Second, the environmental conditions of police custody facilities were a common key focus in non-DUMA information sources. Third, non-DUMA sources had more commonly reported psychosocial and structural safety determinants of health, such as the presence of psychological distress or exposure to violence.

### Information on health conditions

**Fig 4:**
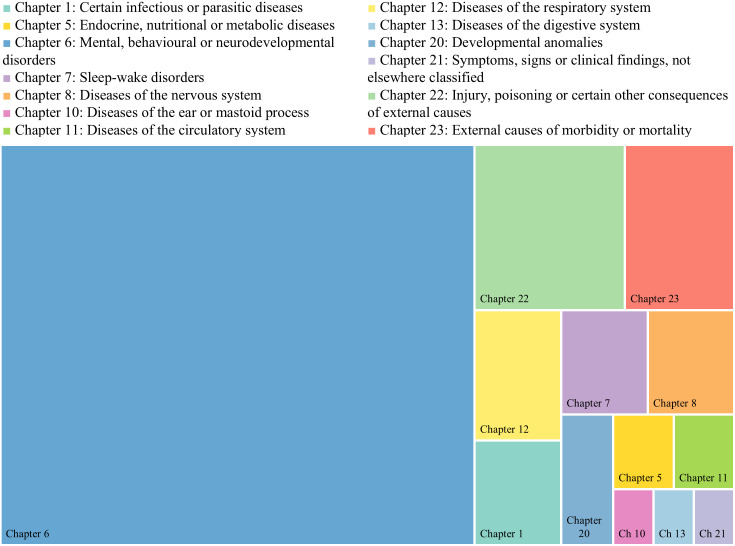
Treemap chart: Information sources reporting diseases by ICD-11 chapter. This treemap chart illustrates the distribution of health conditions reported in information sources, categorized according to ICD-11 chapters. Each ICD-11 chapter is represented with a different color. The size of each block represents the relative frequency with which conditions from that chapter appeared.

In total, 85 information sources included quantitative or qualitative descriptions of illnesses or diseases in people in police custody (65 grey literature and 20 academic publications). Information sources that included data on health conditions most frequently reported those belonging to Chapter 6 of the ICD-11, which includes mental health, behavioral or neurodevelopmental disorders (Fig 4), including drug dependence. This finding was consistent across both academic and grey literature information sources (see respective tabs of S7 Appendix). Nearly all information sources (n = 84) that contained health condition data, included information on at least one mental health, behavioral or neurodevelopmental condition. Other frequently described conditions were from Chapter 22 “Injury, poisoning or certain other consequences of external causes” and Chapter 23 “External causes of morbidity or mortality”. No information sources described people in police custody facilities with conditions from 10 chapters of ICD-11 (Table 5).

**Table 5:** Reported health conditions by ICD-11 chapter.

Chapter of ICD-11	Number of information sources
Chapter 1: Certain infectious or parasitic diseases	4 (2.33%)
Chapter 2: Neoplasms	0
Chapter 3: Diseases of the blood or blood-forming organs	0
Chapter 4: Diseases of the immune system	0
Chapter 5: Endocrine, nutritional or metabolic diseases	2 (1.16%)
Chapter 6: Mental, behavioural or neurodevelopmental disorders	84 (48.84%)
Chapter 7: Sleep-wake disorders	4 (2.33%)
Chapter 8: Diseases of the nervous system	4 (2.33%)
Chapter 9: Diseases of the visual system	0
Chapter 10: Diseases of the ear or mastoid process	1 (0.58%)
Chapter 11: Diseases of the circulatory system	2 (1.16%)
Chapter 12: Diseases of the respiratory system	5 (2.91%)
Chapter 13: Diseases of the digestive system	1 (0.58%)
Chapter 14: Diseases of the skin	0
Chapter 15: Diseases of the musculoskeletal system or connective tissue	0
Chapter 16: Diseases of the genitourinary system	0
Chapter 17: Conditions related to sexual health	0
Chapter 18: Pregnancy, childbirth or the puerperium	0
Chapter 19: Certain conditions originating in the perinatal period	0
Chapter 20: Developmental anomalies	3 (1.74%)
Chapter 21: Symptoms, signs or clinical findings, not elsewhere classified	1 (0.58%)
Chapter 22: Injury, poisoning or certain other consequences of external causes	11 (6.40%)
Chapter 23: External causes of morbidity or mortality	8 (4.65%)

### Information on causes of death

In total, 41 information sources discussed deaths in police custody. Of these, 39 included specific causes of death which could be mapped to ICD-11. Of the sources about deaths in police custody, 30 used data directly from the NDICP. Very few sources (n = 8) reported on deaths in police custody where specific causes of death were mentioned and where the deaths or injuries occurred in police facilities [6,17,31–36]. This was in part due to most sources reporting data from NDICP, which includes in their definition of police custody those being pursued, apprehended, or transported to custodial facilities, and does not disaggregate cause of death data by those held in police custodial facilities. Therefore, information on causes of death for individuals in police custody frequently included deaths due to gunshot wounds or motor vehicle accidents during pursuit or apprehension of suspects. Aside from the yearly NDICP reports, some sources did not differentiate causes of death that occurred while in police custody compared to after recent contact with police [37,38], or causes of death of people in police custody facilities rather than other custodial settings [18,39]. For sources that discussed deaths occurring in police custody facilities, causes of death were wide ranging and included death from injuries (either self-inflicted, or due to assault), ischemic heart disease, respiratory failure, hanging, drug withdrawal or overdose, pneumonia, unexpected death in epilepsy, sepsis, and brain hemorrhage [6,17,31–36].

## Discussion

The aim of this scoping review was to identify and describe the available information on the health and determinants of health of individuals in police custody in Australia. We reviewed 172 information sources that met our inclusion criteria and identified four key findings.

First, the available information is dominated by grey literature publications and more than half of all publications used data from a single source – the DUMA program. Having more than half of all information sources on this topic from this single source raises several issues. The DUMA program is not intended to be a representative sample of people in police custody facilities in Australia, especially given it does not collect data from every state and territory. In the latest yearly report published in 2022, data was collected from five metropolitan sites (Adelaide, Bankstown, Brisbane, Perth and Surry Hills) and, on average, 20% of potentially eligible individuals were deemed unfit for participation in the study [10]. This determination is made by the police officer in charge based on the “level of risk” a detainee may pose to an interviewer [10]. In addition, in more recent years, the DUMA program has excluded juvenile detainees. Importantly, except where a specific health addendum questionnaire was added, the DUMA program only provided indirect evidence on the health of people in police custody facilities related to select socioeconomic determinants of health, substance use, and self-reported substance dependence. The lack of peer-reviewed, academic literature further highlights a lack of rigorous and targeted information on the health of people in police custody facilities in Australia and raises the possibility that researchers face barriers accessing participants in this setting [40]. We were only able to identify 31 academic sources published over a 25-year period.

Second, there is limited breadth of information available on the determinants of health and health status of people in police custody facilities. While many information sources included basic demographics, select socioeconomic determinants, and alcohol and illicit drug use data, there was sparse reporting of other important determinants of health such as tobacco use, diet, exercise, stress, and exposure to violence. Information sources including data on health conditions most commonly focused on a single aspect of health – mental health. We identified almost twice as many sources that included information on mental health conditions than for all other health conditions combined. In addition, we identified no general health needs analyses or health surveys that aimed to capture the burden of disease among police detainees. One source from the Australian Capital Territory audited the activities and duties of police custody forensic physicians, which may have provided an indirect indication on the most common health conditions in this population, but unfortunately did not disclose the diagnoses of patients seen by the service [41]. The information gaps identified reflect priorities on data collection and reporting rather than an absence of health issues. Surveillance systems have historically prioritized substance use and demographic profiling over comprehensive health assessments, resulting in a literature base that disproportionately reports on mental health while neglecting physical health, psychosocial factors, and environmental conditions.

Third is the limited systematic reporting of patterns of deaths in custody. While individual investigations into deaths and coroners’ reports were not included in our review, we identified 41 information sources that discussed deaths in police custody facilities. However, 30 of these only used data from the NDICP, where the specific causes of deaths for those that died while in police facility cells could not be disaggregated from those that died while being pursued or arrested by police. Many more people die in Australia each year while being pursued or arrested by police than those that die in police custody facilities [11]. Where deaths in custodial facilities rather than during pursuit or arrest is the primary interest, the utility of the yearly aggregate data reported through the NDICP becomes extremely limited.

Our findings contrast with the literature and information available from other high-income, Western countries, where large studies have assessed the health needs of police detainees. For example, in the Netherlands, multiple studies have compared the medical complaints and presentations of police detainees with the general population [42,43]. These studies have identified that compared to the general population, police detainees are more likely to be clinically underweight, have chronic pulmonary problems, chronic joint inflammation, hypertension, and serious heart conditions [42]. In the UK, police detainees were found to have high rates of existing physical illnesses, undiagnosed and untreated acute conditions, in addition to higher rates of psychiatric illness than the general population [44,45]. In France, one study reported that somatic diagnoses were even higher than psychiatric diagnoses in their police custody population [46].

### Implications

This review has implications for guiding further research and advocacy. First, we recommend the expansion of academic research into the health and determinants of health of people in police custody, beyond its current scope of mental health and substance dependence. General health needs analyses of this population, like those completed in the UK, Netherlands and France would provide important data for evaluating the services responsible for detainee health and informing quality improvements. Second, we recommend the adoption of a systematic approach for data collection and public reporting on the health of police detainees. The establishment of a minimum data set for health screening, and implementation of health surveillance would increase transparency, and enable evidence-based policy development to improve health outcomes among detainees. Third, we recommend the further operationalization of Australia’s Optional Protocol to the Convention Against Torture and Other Cruel, Inhuman or Degrading Treatment or Punishment (OPCAT) obligations. Although Australia ratified OPCAT in 2017, it remains in breach of its obligations, with three jurisdictions (Victoria, New South Wales, and Queensland) not yet represented by the National Preventative Mechanism (NPM) as of 2025 [47]. OPCAT related inspections and reports have already started to improve transparency into the conditions in police custody in the Australian Capital Territory and external Australian Territories [28,29,48,49]. We recommend the expansion of these inspections to all police custody facilities, and the consistent publication of inspection reports.

### Limitations

This review has several limitations. First, due to resource limitations, data charting was completed by a single author and double checked by a second author, as opposed to the preferred method of double blinded data extraction with reconciliation. This may have increased the risk of data charting errors. Second, the scope of this review was limited to exclude coroner reports and investigations into single deaths in custody. Accordingly, the findings related to deaths in police custody facilities and their cause of death should be interpreted cautiously. This is especially important given many information sources we identified did not disaggregate deaths that occurred in police custody facilities from deaths during other interactions with police. Instead, other sources which exclusively focus on summarizing the findings from coroner reports on deaths in custody in Australia, should be consulted for further information on this topic [17,18,38]. Third, we chose to investigate a discrete list of determinants of health, based on the AIHW framework, to limit this scope of work to an achievable size. For example, determinants relevant to detainees’ families (such as a history of childhood trauma, family history of incarceration, marital status, or having dependent children) and environmental determinants (such as access to natural light, or privacy in showering and bathroom facilities) were not included in this review. Fourth, we acknowledge that the exclusion of information sources that did not include primary data means some important perspectives and insights into the health of people in police custody may have been missed. For example, during screening we encountered several articles from the criminology, sociology, and legal fields, that explored systemic racism and structural issues within the criminal legal system. Fifth, we limited our analysis to information sources published from 2000. While this decision improved the feasibility of the review, the shortened timeframe may have meant that our study missed important insights from earlier information sources.

## Conclusion

This comprehensive review mapped the currently available information on the health and determinants of health of people in police custody in Australia, and found that it is fragmented, predominantly derived from a single program, and focused on mental health and substance use. To better direct health service design, monitoring, and evaluation, we call for more transparent publication of health data of people in police custody facilities, particularly where key information is lacking, such as for physical health conditions. Increased transparency and information generation will be critical for providing an environment conducive to promoting rather than undermining the health of people in police custody facilities.

## Supporting information

S1 Appendix Database searches. (DOCX)

S2 Appendix Grey literature website log. (DOCX)

S3 Appendix Reference search of relevant review articles. (DOCX)

S4 Appendix Excluded studies. (DOCX)

S5 Appendix Data charting tool. (XLSM)

S6 Appendix Included information sources. This appendix includes two tables which contain the characteristics of all included academic and grey literature information sources. This includes each information source’s jurisdiction of data collection, study design, sample size, aim, methods and conclusions.
(DOCX)

S7 Appendix Spreadsheet including all charted data. This spreadsheet contains all data charted from included information sources. The first tab provides a table of contents and explanation of the contents of the following eight tabs.
(XLSM)

S8 Appendix Publications per year by jurisdiction. A figure is provided for each jurisdiction in Australia showing the number of identified publications each year between 2000 and 2024.
(DOCX)

S9 Fig Co-occurrence heat map: All information sources. This heatmap visualizes the frequency with which pairs of health determinants co-occurred across all included sources. Each cell represents a unique determinant pairing, with deeper shades of red indicating higher co-occurrence. The diagonal from top left to bottom right represents the frequency of each determinant appearing independently. Blank or pale cells indicate little to no co-occurrence.
(TIF)

S10 Fig Co-occurrence heat map: Sources utilizing DUMA data. This heatmap visualizes the frequency with which pairs of health determinants co-occurred across sources that utilized data from the Drug Use Monitoring in Australia (DUMA) program. Each cell represents a unique determinant pairing, with deeper shades of red indicating higher co-occurrence. The diagonal from top left to bottom right represents the frequency of each determinant appearing independently. Blank or pale cells indicate little to no co-occurrence. This figure shows that sources utilizing DUMA program data only reported on selected determinants, and did not include any information on others, such as the environmental conditions in police custody.
(TIF)

S11 Fig Co-occurrence heat map: Sources not utilizing DUMA data. This heatmap visualizes the frequency with which pairs of health determinants co-occurred across sources that did not utilize data from the Drug Use Monitoring Australia (DUMA) program. Each cell represents a unique determinant pairing, with deeper shades of red indicating higher co-occurrence. The diagonal from top left to bottom right represents the frequency of each determinant appearing independently. Blank or pale cells indicate little to no co-occurrence. This figure shows that, outside of the DUMA program, there is some information available on health behavior, safety, and environmental determinants, which were largely absent from DUMA program publications (see S10 Fig).
(TIF)

S12 Appendix Preferred Reporting Items for Systematic reviews and Meta-Analyses extension for Scoping Reviews (PRISMA-ScR) Checklist. (DOCX)
