# Supplementary material for: The determinants of health and health status of individuals in police custody in Australia: A scoping review
Source: PLoS One. 2025 Dec 30;20(12):e0338957. doi: 10.1371/journal.pone.0338957 (PMC12753082; doi:10.1371/journal.pone.0338957)
Supplement: S1 Appendix — (DOCX) [file pone.0338957.s001.docx]

# **S1 Appendix: Database Searches**

## **MEDLINE (Ovid) Search: 2nd July 2024 & 3^rd^ February 2025**

**Database:** Ovid MEDLINE(R) and Epub Ahead of Print, In-Process, In-Data-Review & Other Non-Indexed Citations, Daily and Versions 1946 to June 28, 2024 (and then, to January 31, 2025)

| **#** | **Query** | **Results 02.07.2024** | **Results 03.02.2025** |
| --- | --- | --- | --- |
| 1 | (Prisoner/ or Criminals/) and Police/ | 423 | 428 |
| 2 | ((police adj5 custody) or (police adj3 detainee*) or (police adj3 detention) or (police adj3 cell*) or Detainee* or Watch house* or Watchhouse* or Custody suite* or Short term custody or (police adj5 lockup*) or  Police lock-up* or (police adj5 watch house)).mp. | 1468 | 1492 |
| 3 | police custodial.mp. | 6 | 6 |
| 4 | ((law enforcement officer* or police force* or police officer* or shortterm or short term) adj3 custod*).mp. | 14 | 14 |
| 5 | police station*.mp. | 352 | 363 |
| 6 | 1 or 2 or 3 or 4 or 5 | 2056 | 2095 |
| 7 | Australia/ or Northern Territory/ or Victoria/ or New South Wales/ or Queensland/ or Tasmania/ or South Australia/ or Western Australia/ or Australian Capital Territory/ | 176,571 | 181,049 |
| 8 | (Australia* or Northern Territory or Victoria* or New South Wales or Queensland* or Tasmania* or Sydney or Melbourne or Brisbane or Perth or Adelaide or Canberra or Hobart or Gold Coast or Newcastle or Sunshine Coast or Central Coast or Wollongong or Geelong or Townsville or Cairns or Toowoomba or Darwin or Ballarat or Bendigo or Albury-Wodonga or Torres Strait Island* or Alice Springs).mp,cp,in. | 1,306,031 | 1,356,389 |
| 9 | 7 or 8 | 1,306,031 | 1,356,389 |
| 10 | 6 and 9 | 192 | 198 |
| 11 | limit 10 to yr="2000 - 2025" | 172 | 178 |

## **Embase (Ovid) Search: 2nd July 2024 & 3^rd^ February 2025**

**Database:** Embase 1974 to 2024 June 28 & to 2025 January 31 respectively.

| **#** | **Query** | **Results**  **02.07.2024** | **Results**  **03.02.2025** |
| --- | --- | --- | --- |
| 1 | (offender/ or prisoner/) and police/ | 1,149 | 1185 |
| 2 | pretrial detention/ or detention center/ | 213 | 234 |
| 3 | 1 or 2 | 1,361 | 1,418 |
| 4 | (police station* or police custodial).mp. | 515 | 522 |
| 5 | ((law enforcement officer* or police force* or police officer* or shortterm or short term) adj3 custod*).mp. | 17 | 17 |
| 6 | ((police adj5 custody) or (police adj3 detainee*) or (police adj3 detention) or (police adj3 cell*) or Detainee* or Watch house* or Watchhouse* or Custody suite* or Short term custody or (police adj5 lockup*) or Police lock-up* or (police adj5 watch house)).mp. | 1,802 | 1,821 |
| 7 | 3 or 4 or 5 or 6 | 3,408 | 3,479 |
| 8 | australia/ or australian capital territory/ or new south wales/ or northern territory/ or queensland/ or south australia/ or tasmania/ or victoria/ or western australia/ | 205,291 | 208,995 |
| 9 | (Australia* or Northern Territory or Victoria* or New South Wales or Queensland* or Tasmania* or Sydney or Melbourne or Brisbane or Perth or Adelaide or Canberra or Hobart or Gold Coast or Newcastle or Sunshine Coast or Central Coast or Wollongong or Geelong or Townsville or Cairns or Toowoomba or Darwin or Ballarat or Bendigo or Albury-Wodonga or Torres Strait Island* or Alice Springs).mp,cp,in. | 1,684,073 | 1,721,679 |
| 10 | 8 or 9 | 1,684,073 | 1,721,679 |
| 11 | 7 and 10 | 307 | 319 |
| 12 | limit 11 to yr="2000 - 2025" | 280 | 293 |

## **PsychINFO (Ovid) Search: 2nd July 2024 & 3^rd^ February 2025**

**Database: APA PsychInfo 1806 to June Week 3 2024 & Week 4 January 2025 respectively**

| **#** | **Query** | **Results 02.07.2024** | **Results 03.02.2025** |
| --- | --- | --- | --- |
| 1 | (incarcerated/ or criminal offenders/ or female criminal offenders/ or male criminal offenders/ or mentally ill offenders/) and police personnel/ | 492 | 506 |
| 2 | ((police adj5 custody) or (police adj3 detainee*) or (police adj3 detention) or (police adj3 cell*) or Detainee* or Watch house* or Watchhouse* or Custody suite* or Short term custody or (police adj5 lockup*) or Police lock-up* or (police adj5 watch house)).mp. | 1400 | 1436 |
| 3 | (police custodial or police station*).mp. | 314 | 319 |
| 4 | ((law enforcement officer* or police force* or police officer* or shortterm or short term) adj3 custod*).mp. | 9 | 9 |
| 5 | 1 or 2 or 3 or 4 | 2120 | 2174 |
| 6 | (Australia* or Northern Territory or Victoria* or New South Wales or Queensland* or Tasmania* or Sydney or Melbourne or Brisbane or Perth or Adelaide or Canberra or Hobart or Gold Coast or Newcastle or Sunshine Coast or Central Coast or Wollongong or Geelong or Townsville or Cairns or Toowoomba or Darwin or Ballarat or Bendigo or Albury-Wodonga or Torres Strait Island* or Alice Springs).mp,cp,in. | 267,446 | 275,510 |
| 7 | 5 and 6 | 200 | 205 |
| 8 | limit 7 to yr="2000 - 2024" | 188 | 193 |

## **CINAHL Complete (EBSCOhost) Search: 2nd July 2024 & 3^rd^ February 2025**

**Database: CINAHL Complete**

| **#** | **Query** | **Results 02.07.2024** | **Results 03.02.2025** |
| --- | --- | --- | --- |
| S10 | S5 AND S8  **Limiters** - Publication Date: 2000-01-01-2025-12-31 | 80 | 82 |
| S9 | S5 AND S8 | 85 | 87 |
| S8 | S6 OR S7 | 171,399 | 176,269 |
| S7 | (Australia* or Northern Territory or Victoria* or New South Wales or Queensland* or Tasmania* or Sydney or Melbourne or Brisbane or Perth or Adelaide or Canberra or Hobart or Gold Coast or Newcastle or Sunshine Coast or Central Coast or Wollongong or Geelong or Townsville or Cairns or Toowoomba or Darwin or Ballarat or Bendigo or Albury-Wodonga or Torres Strait Island* or Alice Springs) | 171,399 | 176,269 |
| S6 | (MH "Australia") OR (MH "Australian Capital Territory") OR (MH "New South Wales") OR (MH "Northern Territory") OR (MH "Queensland") OR (MH "South Australia") OR (MH "Tasmania") OR (MH "Victoria") OR (MH "Western Australia") | 131,532 | 134,463 |
| S5 | S1 OR S2 OR S3 OR S4 | 1,545 | 1,572 |
| S4 | (law enforcement officer* or police force* or police officer* or shortterm or short term) n3 custod* | 13 | 14 |
| S3 | police custodial OR police station* | 213 | 201 |
| S2 | (police n5 custody) or (police n3 detainee*) or (police n3 detention) or (police n3 cell*) or Detainee* or Watch house* or Watchhouse* or Custody suite* or Short term custody or (police n5 lockup*) or Police lock-up* or (police n5 watch house) | 851 | 866 |
| S1 | ((MH "Prisoners") or (MH "Public Offenders") or (MH "Mentally Ill Offenders")) AND (MH "Police") | 601 | 609 |

## **Criminal Justice Abstracts (EBSCOhost) Search: 2nd July 2024 & 3^rd^ February 2025**

**Database: Criminal Justice Abstracts**

| **#** | **Query** | **Results 02.07.2024** | **Results 03.02.2025** |
| --- | --- | --- | --- |
| S6 | S4 AND S5   **Limiters** - Publication Date: 2000-01-01-2025-12-31 | 214 | 218 |
| S6 | S4 AND S5 | 249 | 253 |
| S5 | (Australia* or Northern Territory or Victoria* or New South Wales or Queensland* or Tasmania* or Sydney or Melbourne or Brisbane or Perth or Adelaide or Canberra or Hobart or Gold Coast or Newcastle or Sunshine Coast or Central Coast or Wollongong or Geelong or Townsville or Cairns or Toowoomba or Darwin or Ballarat or Bendigo or Albury-Wodonga or Torres Strait Island* or Alice Springs) | 37,059 | 38,121 |
| S4 | S1 OR S2 OR S3 | 2,637 | 2,742 |
| S3 | (law enforcement officer* or police force* or police officer* or shortterm or short term) n3 custod* | 42 | 43 |
| S2 | police custodial OR police station* | 565 | 566 |
| S1 | (police n5 custody) or (police n3 detainee*) or (police n3 detention) or (police n3 cell*) or Detainee* or Watch house* or Watchhouse* or Custody suite* or Short term custody or (police n5 lockup*) or Police lock-up* or (police n5 watch house) | 2,141 | 2,246 |

## **Scopus Search: 2nd July 2024 & 3^rd^ February 2025**

**Database: Scopus**

| **Query** | **Results 02.07.2024** | **Results 03.02.2025** |
| --- | --- | --- |
| ( ( TITLE-ABS-KEY ( ( police W/3 custodial ) OR ( police W/3 station* ) ) ) OR ( TITLE-ABS-KEY ( ( police W/5 custody ) OR ( police W/3 detainee* ) OR ( police W/3 detention ) OR ( police W/3 cell* ) OR ( watch W/4 house* ) OR ( watchhouse* ) OR ( custody W/4 suite* ) OR ( shortterm W/4 custody ) OR ( short-term W/4 custody ) OR ( police W/5 lockup* ) OR ( police W/5 lock-up* ) OR ( police W/5 watchhouse ) ) ) ) AND ( TITLE-ABS-KEY ( australia* OR {Northern Territory} OR victoria* OR {New South Wales} OR queensland* OR tasmania* OR sydney OR melbourne OR brisbane OR perth OR adelaide OR canberra OR hobart OR {Gold Coast} OR newcastle OR {Sunshine Coast} OR {Central Coast} OR wollongong OR geelong OR townsville OR cairns OR toowoomba OR darwin OR ballarat OR bendigo OR albury-wodonga OR {Torres Strait Island*} OR {Alice Springs} ) ) | 163 | 169 |

## **Web of Science Search: 2nd July 2024 & 3^rd^ February 2025**

**Databases:**Science Citation Index Expanded 1900 – Present
Social Sciences Citation Index 1900 – Present
Conference Proceedings Citation Index – Science 1990 – Present
Conference Proceedings Citation Index – Social Science & Humanities 1990 – Present

| **#** | **Query** | **Results 02.07.2024** | **Results 03.02.2025** |
| --- | --- | --- | --- |
| 7 | #5 and #4   Timespan: 2000-01-01 to 2025-12-31 (Publication Date) | 122 | 129 |
| 6 | #5 and #4 | 134 | 141 |
| 5 | TS=((Australia* or “Northern Territory” or Victoria* or “New South Wales” or Queensland* or Tasmania* or Sydney or Melbourne or Brisbane or Perth or Adelaide or Canberra or Hobart or “Gold Coast” or Newcastle or “Sunshine Coast” or “Central Coast” or Wollongong or Geelong or Townsville or Cairns or Toowoomba or Darwin or Ballarat or Bendigo or Albury-Wodonga or “Torres Strait Island*” or Alice Springs)) | 536,257 | 551,217 |
| 4 | #3 OR #2 OR #1 | 3286 | 3379 |
| 3 | TS=(((“law enforcement officer*” or “police force*” or “police officer*” or shortterm or “short term”) near/3 custod*)) | 27 | 29 |
| 2 | TS= (“police custodial” or “police station*”) | 773 | 804 |
| 1 | TS=(((police near/5 custody) or (police near/3 detainee*) or (police near/3 detention) or (police near/3 cell*) or Detainee* or “watch house*” or Watchhouse* or Custody suite* or “Short term custody” or (police near/5 lockup*) or “Police lock-up*” or (police near/5 watch house))) | 2,577 | 2,641 |

## **Criminal Justice Database (ProQuest): 2^nd^ July 2024 & 3^rd^ February 2025**

**Databases: Criminal Justice Database (ProQuest) 1981 - Present**

| **#** | **Query** | **Results 02.07.2024** | **Results 03.02.2025** |
| --- | --- | --- | --- |
| S7 | [S4] AND [S5] limit 2000-01-01 - 2024-12-31 | 123 | 126 |
| S6 | [S4] AND [S5] | 133 | 137 |
| S5 | noft(((Australia* or “Northern Territory” or Victoria* or “New South Wales” or Queensland* or Tasmania* or Sydney or Melbourne or Brisbane or Perth or Adelaide or Canberra or Hobart or “Gold Coast” or Newcastle or “Sunshine Coast” or “Central Coast” or Wollongong or Geelong or Townsville or Cairns or Toowoomba or Darwin or Ballarat or Bendigo or Albury-Wodonga or “Torres Strait Island*” or “Alice Springs”)) ) | 25,145 | 26,088 |
| S4 | [[S1] OR [S2] OR [S3]](https://www.proquest.com/recentsearches.recentsearchtabview.recentsearchesgridview.scrolledrecentsearchlist.checkdbssearchlink:rerunsearch/A4226A32997A40CBPQ/None/$N?site=criminaljusticeperiodicals&t:ac=RecentSearches) | 4054 | 4132 |
| S3 | [noft(((“law enforcement officer*” or “police force*” or “police officer*” or shortterm or “short term”) near/3 custod*))](https://www.proquest.com/recentsearches.recentsearchtabview.recentsearchesgridview.scrolledrecentsearchlist.checkdbssearchlink:rerunsearch/F6B71C472538413BPQ/None/$N?site=criminaljusticeperiodicals&t:ac=RecentSearches) | 25 | 25 |
| S2 | [noft(“police custodial” or “police station*” )](https://www.proquest.com/recentsearches.recentsearchtabview.recentsearchesgridview.scrolledrecentsearchlist.checkdbssearchlink:rerunsearch/BF277C1DDA2542C1PQ/None/$N?site=criminaljusticeperiodicals&t:ac=RecentSearches) | 1048 | 1075 |
| S1 | [noft(((police near/5 custody) or (police near/3 detainee*) or (police near/3 detention) or (police near/3 cell*) or Detainee* or “watch house*” or Watchhouse* or “Custody suite*” or “Short term custody” or (police near/5 lockup*) or “Police lock-up*” or (police near/5 “watch house*”)))](https://www.proquest.com/recentsearches.recentsearchtabview.recentsearchesgridview.scrolledrecentsearchlist.checkdbssearchlink:rerunsearch/27F2C78920F8479DPQ/None/$N?site=criminaljusticeperiodicals&t:ac=RecentSearches) | 3055 | 3107 |

## **Grey Literature Database (Informit) Search 4th July 2024**

- Database 1: POLICY from the Analysis and Policy Observatory
- Database 2: Australian Public Affairs Full Text
- Database 3: Aboriginal and Torres Strait Islander Health Bibliography
- Database 4: Australian Criminology Database
- Database 5: AGIS Plus Text

No MeSH terms or proximity operators are available through Informit. Individual key words were tested, and then whole keyword searches from the Medline search were adapted for use on Informit. The same search was repeated individually on databases 1-4 and the results for each search line are shown below. For database 5, AGIS Plus Text, additional terms were needed to localize results to Australia.

**Databases 1-4**

|  |  | **Database** | | | | |
| --- | --- | --- | --- | --- | --- | --- |
| **#** | **Query** | **1** | **2** | **3** | **4** |  |
| 1 | (Police custody) OR (Police detainee*) OR (police detention) OR (police cell*) OR (Detainee*) OR (Watch house*) OR (Watchhouse*) OR (Custody suite*) OR (Short term custody) OR (police lockup*) OR (Police lock-up*) OR (police watch house) | 124 | 997 | 44 | 1,490 | **Results 02.07.2024** |
|  |  | 125 | 1,001 | 44 | 1,490 | **Results 03.02.2025** |
| 2 | (Stroke*) OR (Asthma) OR (Type 2 Diabetes mellitus ) OR (Diabetes mellitus) OR (T2DM) OR (COPD) OR (Chronic obstructive pulmonary disease) OR (Cardiovascular disease*) OR (Arthriti*) OR (Neoplasm*) OR (Asphyxia*) OR (Respiratory aspiration*) OR (Suicid*) OR (intoxication) OR (Psychiatric diagnos*) OR (Psychiatric symptom*) OR (Mental disorder*) OR (Mental illness*) OR (Schizophren*) OR (psychotic) OR (Psychosis) OR (Mental health) OR (Anxiety) OR (Anxiety disorder) OR (Mood disorder) OR (Depression) OR (Injecting drug) OR (Substance abuse) OR (Alcohol related disorder) OR (Drug dependenc*) OR (Alcohol dependenc*) OR (Wound*) OR (Injur*) OR (Concussion) OR (Fracture*) OR (Haemorrhage*) OR (Hemorrhage*) OR (Contusion*) OR (Bruis*) OR (Needs assessment) OR (Emergency service*) OR (Forensic medicine) OR (Screening) OR (Nursing care) OR (Medical care) OR (Intervention) OR (Treatment strateg*) OR (Treatment*) OR (Medication*) OR (Mental health service) OR (Medical history) OR (Prescrib*) OR (Diagnos*) OR (Resuscitation) OR (Opioid substitution therapy) OR (Suicide prevention) OR (morbidity) OR (Mortality) OR (Complex Needs) OR (Health care) OR (Health) OR (Wellbeing) OR (Disease*) OR (Illness*) OR (Diagnos*) OR (Death*) OR (Disabilit*) OR (Burden of disease) OR (Prevalence) | 10,557 | 207,877 | 32,116 | 21,865 | **Results 02.07.2024** |
|  |  | 10,774 | 209,287 | 32116 | 21,865 | **Results 03.02.2025** |
| 3 | Combining 1 and 2 through advanced search function | 77 | 511 | 44 | 1,007 | **Results 02.07.2024** |
|  |  | 78 | 514 | 44 | 1,007 | **Results 03.02.2025** |
| 4 | Limiting to 2000-2025 | 76 | 378 | 28 | 563 | **Results 02.07.2024** |
|  |  | 77 | 380 | 28 | 563 | **Results 03.02.2025** |

**Database 5: AGIS Plus Text**

| **#** | **Query** | **Results 02.07.2024** | **Results 03.02.2025** |
| --- | --- | --- | --- |
| 1 | (Police custody) OR (Police detainee*) OR (police detention) OR (police cell*) OR (Detainee*) OR (Watch house*) OR (Watchhouse*) OR (Custody suite*) OR (Short term custody) OR (police lockup*) OR (Police lock-up*) OR (police watch house) | 1,203 | 1,233 |
| 2 | (Stroke*) OR (Asthma) OR (Type 2 Diabetes mellitus ) OR (Diabetes mellitus) OR (T2DM) OR (COPD) OR (Chronic obstructive pulmonary disease) OR (Cardiovascular disease*) OR (Arthriti*) OR (Neoplasm*) OR (Asphyxia*) OR (Respiratory aspiration*) OR (Suicid*) OR (intoxication) OR (Psychiatric diagnos*) OR (Psychiatric symptom*) OR (Mental disorder*) OR (Mental illness*) OR (Schizophren*) OR (psychotic) OR (Psychosis) OR (Mental health) OR (Anxiety) OR (Anxiety disorder) OR (Mood disorder) OR (Depression) OR (Injecting drug) OR (Substance abuse) OR (Alcohol related disorder) OR (Drug dependenc*) OR (Alcohol dependenc*) OR (Wound*) OR (Injur*) OR (Concussion) OR (Fracture*) OR (Haemorrhage*) OR (Hemorrhage*) OR (Contusion*) OR (Bruis*) OR (Needs assessment) OR (Emergency service*) OR (Forensic medicine) OR (Screening) OR (Nursing care) OR (Medical care) OR (Intervention) OR (Treatment strateg*) OR (Treatment*) OR (Medication*) OR (Mental health service) OR (Medical history) OR (Prescrib*) OR (Diagnos*) OR (Resuscitation) OR (Opiate substitution therapy) OR (Suicide prevention) OR (morbidity) OR (Mortality) OR (Complex Needs) OR (Health care) OR (Health) OR (Wellbeing) OR (Disease*) OR (Illness*) OR (Diagnos*) OR (Death*) OR (Disabilit*) OR (Burden of disease) OR (Prevalence) | 61,039 | 62,936 |
| 3 | (Australia*) OR (Northern Territory) OR (Victoria*) OR (New South Wales) OR (Queensland*) OR (Tasmania*) OR (Sydney) OR (Melbourne) OR (Brisbane) OR (Perth) OR (Adelaide) OR (Canberra) OR (Hobart) OR (Gold Coast) OR (Newcastle) OR (Sunshine Coast) OR (Central Coast) OR (Wollongong) OR (Geelong) OR (Townsville) OR (Cairns) OR (Toowoomba) OR (Darwin) OR (Ballarat) OR (Bendigo) OR (Albury-Wodonga) OR (Torres Strait Island*) OR (Alice Springs) | 292,851 | 294,039 |
| 4 | 1 AND 2 | 503 | 516 |
| 5 | 1 AND 2 AND 3 | 353 | 358 |
| 6 | Limiting to 2000-2025 | 295 | 298 |
