# Supplementary material for: The determinants of health and health status of individuals in police custody in Australia: A scoping review
Source: PLoS One. 2025 Dec 30;20(12):e0338957. doi: 10.1371/journal.pone.0338957 (PMC12753082; doi:10.1371/journal.pone.0338957)
Supplement: S2 Appendix — (DOCX) [file pone.0338957.s002.docx]

# **S2 Appendix: Grey Literature Website Log**

**Contents**

[Grey Literature Searches: National Websites 2](#_Toc200636932)

[Grey Literature Searches: Australian Capital Territory Websites 12](#_Toc200636933)

[Grey Literature Searches: New South Wales Websites 17](#_Toc200636934)

[Grey Literature Searches: Northern Territory Websites 26](#_Toc200636935)

[Grey Literature Searches: Queensland Websites 30](#_Toc200636936)

[Grey Literature Searches: South Australian Websites 35](#_Toc200636937)

[Grey Literature Searches: Tasmanian Websites 39](#_Toc200636938)

[Grey Literature Searches: Victorian Websites 43](#_Toc200636939)

[Grey Literature Searches: Western Australian Websites 51](#_Toc200636940)

[Articles Referred for Second Review 55](#_Toc200636941)

## Grey Literature Searches: National Websites

**Commonwealth Ombudsman**

Website: [www.ombudsman.gov.au](http://www.ombudsman.gov.au)

| **Search Date** | **Search Term/s** | **Limits / Filters** | **Number of Results** | **Relevant new studies/reports identified, reviewed at full text and referred for second review** |
| --- | --- | --- | --- | --- |
| 13.01.2025 | Pre-defined keyword:  “Police Custody” | None | 81 | 1. Post Visit Summary: Christmas Island Police Station  2. Post Visit Summary: Cocos Island Police Station  3. Post Visit Summaries: ACT Policing Watch House and ACT Police Stations |
| 13.01.2025 | Iterative follow up search:  “Post Visit Summary” | None | 143 | 4. Post Visit Summary: Jervis Bay |
| 13.01.2025 | Pre-defined keyword:  “Police Cells” | None | 56 | None |
| 13.01.2025 | Pre-defined keyword:  “Custodial Care” | None | 49 | None |
| 13.01.2025 | Pre-defined keyword:  “Watchhouse” | None | 17 | None |

**Commonwealth Director of Public Prosecutions**

Website: [www.cdpp.gov.au](http://www.cdpp.gov.au)

| **Search Date** | **Search Term/s** | **Limits / Filters** | **Number of Results** | **Relevant studies/reports reviewed at full text and referred for second review** |
| --- | --- | --- | --- | --- |
| 13.01.2025 | Pre-defined keyword: “Police Custody” | None | 14 | None |
| 13.01.2025 | Pre-defined keyword: “Police Cells” | None | 0 | None |
| 13.01.2025 | Pre-defined keyword: “Police Detainees” | None | 0 | None |
| 13.01.2025 | Pre-defined keyword: “Custodial Care” | None | 5 | None |
| 13.01.2025 | Pre-defined keyword: “Police Detention” | None | 10 | None |
| 13.01.2025 | Pre-defined keyword: “Policing” | None | 10 | None |
| 13.01.2025 | Pre-defined keyword: “Offenders” | None | 45 | None |
| 13.01.2025 | Pre-defined keyword: “Watchhouse” | None | 1 | None |
| 13.01.2025 | Pre-defined keyword: “Custody Suite” | None | 2 | None |
| 13.01.2025 | Pre-defined keyword:  “Short term custody” | None | 2 | None |
| 13.01.2025 | Pre-defined keyword:  “Police watchhouse” | None | 1 | None |

**Human Rights Law Centre**

Website: <https://www.hrlc.org.au/>

No search function available. Page browsing completed instead.

| **Date** | **Pages Browsed** | **Relevant studies/reports reviewed at full text and referred for second review** |
| --- | --- | --- |
| 17.01.2025 | Our Work: Reports | None |
| 17.01.2025 | Our Work: News and Updates | None |
| 17.01.2025 | Our Work: Media Releases | None |

**Australian Federal Police**

Website: [www.afp.gov.au](http://www.afp.gov.au)

Searches Completed:

| **Search Date** | **Search Term/s** | **Limits / Filters** | **Number of Results** | **Relevant studies/reports reviewed at full text and referred for second review** |
| --- | --- | --- | --- | --- |
| 17.01.2025 | Pre-defined keyword: Health | “Content” selected, not “News Centre” | 26 | None |
| 17.01.2025 | Pre-defined keyword:  Mental health | “Content” selected, not “News Centre” | 5 | None |
| 17.01.2025 | Pre-defined keyword:  Medical care | “Content” selected, not “News Centre” | 4 | None |
| 17.01.2025 | Pre-defined keyword:  Treatment | “Content” selected, not “News Centre” | 14 | None |
| 17.01.2025 | Pre-defined keyword:  Medication | “Content” selected, not “News Centre” | 17 | None |
| 17.01.2025 | Pre-defined keyword:  Disease | “Content” selected, not “News Centre” | 5 | None |
| 17.01.2025 | Pre-defined keyword:  Diagnosis | “Content” selected, not “News Centre” | 5 | None |
| 17.01.2025 | Pre-defined keyword:  Morbidity | “Content” selected, not “News Centre” | 0 | None |
| 17.01.2025 | Pre-defined keyword:  Mortality | “Content” selected, not “News Centre” | 0 | None |
| 17.01.2025 | Pre-defined keyword:  Disability | “Content” selected, not “News Centre” | 13 | None |
| 17.01.2025 | Pre-defined keyword:  Prevalence | “Content” selected, not “News Centre” | 0 | None |
| 17.01.2025 | Pre-defined keyword:  Incidence | “Content” selected, not “News Centre” | 28 | None |
| 17.01.2025 | Pre-defined keyword:  Watchhouse | “Content” selected, not “News Centre” | 0 | None |
| 17.01.2025 | Pre-defined keyword:  Police Cells | “Content” selected, not “News Centre” | 1 | None |

**Australia and New Zealand Policing Advisory Agency**

Website: www.anzpaa.org.au

| **Search Date** | **Search Term/s** | **Limits / Filters** | **Number of Results** | **Relevant studies/reports reviewed at full text and referred for second review** |
| --- | --- | --- | --- | --- |
| 17.01.2025 | Pre-defined keyword: Health | None | 33 | None |
| 17.01.2025 | Pre-defined keyword:  Mental health | None | 32 | None |
| 17.01.2025 | Pre-defined keyword:  Medical care | None | 12 | None |
| 17.01.2025 | Pre-defined keyword:  Treatment | None | 3 | None |
| 17.01.2025 | Pre-defined keyword:  Medication | None | 0 | None |
| 17.01.2025 | Pre-defined keyword:  Disease | None | 2 | None |
| 17.01.2025 | Pre-defined keyword:  Diagnosis | None | 0 | None |
| 17.01.2025 | Pre-defined keyword:  Morbidity | None | 0 | None |
| 17.01.2025 | Pre-defined keyword:  Mortality | None | 0 | None |
| 17.01.2025 | Pre-defined keyword:  Disability | None | 0 | None |
| 17.01.2025 | Pre-defined keyword:  Prevalence | None | 5 | None |
| 17.01.2025 | Pre-defined keyword:  Incidence | None | 2 | None |
| 17.01.2025 | Iterative search:  Deaths in custody | None | 7 | None |
| 17.01.2025 | Iterative search:  Detainee | None | 0 | None |
| 17.01.2025 | Pre-defined keyword:  watchhouse | None | 0 | None |
| 17.01.2025 | Pre-defined keyword:  Custody suite | None | 5 | None |

Additional pages browsed:

| **Date** | **Pages Browsed** | **Relevant studies referred for second screen** |
| --- | --- | --- |
| 17.01.2025 | Publications | None |
| 17.01.2025 | Services | None |
| 17.01.2025 | Services: Research & Innovation | None |

**Australian Human Rights Commission**

Website: [www.humanrights.gov.au](http://www.humanrights.gov.au)

| **Search Date** | **Search Term/s** | **Limits / Filters** | **Number of Results** | **Relevant studies/reports reviewed at full text and referred for second review** |
| --- | --- | --- | --- | --- |
| 17.01.2025 | Pre-defined keyword:  Police custody | None | 272 | None |
| 17.01.2025 | Pre-defined keyword:  Police cells | None | 171 | Search refined, see below |
| 17.01.2025 | Pre-defined keyword:  Police cells | Excluding media releases, opinion pieces, and news stories | 139 | None. Many results repeated from Police Custody search. |
| 17.01.2025 | Pre-defined keyword:  Police detainees | Excluding media releases, opinion pieces, news stories and speeches | 158 | None. Many results repeated from Police Custody and Police Cells search. |
| 17.01.2025 | Pre-defined keyword:  Watchhouse | None | 21 | None |
| 17.01.2025 | Pre-defined keyword:  Police watchhouse | None | 20 | None |

**Australian Commission on Safety and Quality in Health Care**

Website: [www.safetyandquality.gov.au](http://www.safetyandquality.gov.au)

| **Search Date** | **Search Term/s** | **Limits / Filters** | **Number of Results** | **Relevant studies/reports reviewed at full text and referred for second review** |
| --- | --- | --- | --- | --- |
| 17.01.2025 | Pre-defined keyword:  Police custody | None | 0 | None |
| 17.01.2025 | Pre-defined keyword:  Police cells | None | 0 | None |
| 17.01.2025 | Iterative search:  Police | None | 4 | None |
| 17.01.2025 | Pre-defined keyword:  Watchhouse | None | 0 | None |
| 17.01.2025 | Iterative search:  Detainee | None | 0 | None |
| 17.01.2025 | Pre-defined keyword:  Custodial care | None | 2 | None |

**Australian Government Department of Health and Aged Care**

Website: [www.health.gov.au](http://www.health.gov.au)

| **Search Date** | **Search Term/s** | **Limits / Filters** | **Number of Results** | **Relevant studies/reports reviewed at full text and referred for second review** |
| --- | --- | --- | --- | --- |
| 17.01.2025 | Pre-defined keyword:  Police custody | None | 65 | None |
| 17.01.2025 | Pre-defined keyword:  Police cells | None | 201 | None |
| 20.01.2025 | Pre-defined keyword:  Police detainees | None | 59 | None |
| 20.01.2025 | Pre-defined keyword:  Police detention | None | 64 | None |
| 20.01.2025 | Pre-defined keyword:  Watchhouse | None | 0 | None |
| 20.01.2025 | Pre-defined keyword:  Short term custody | None | 308 | None |
| 20.01.2025 | Pre-defined keyword:  Police watchhouse | None | 59 | None |

**Australian Bureau of Statistics**

Website: [www.abs.gov.au](http://www.abs.gov.au)

| **Search Date** | **Search Term/s** | **Limits / Filters** | **Number of Results** | **Relevant studies/reports reviewed at full text and referred for second review** |
| --- | --- | --- | --- | --- |
| 20.01.2025 | Pre-defined keyword: “Police Custody” | None | 985 | Notes: reviewed first 200 results, all relating to either police or custody, not “police custody” Search refined – see below. |
| 20.01.2025 | Pre-defined keyword: “Police Custody” | Phrase Search | 30 | None  Notes:  Death in Custody data is summarised a re-published in ABS “Year Book Australia” publications.  ABS “Prisoners in Australia” reports specific exclude people in Police Custody. |
| 20.01.2025 | Pre-defined keyword: “Police Cells” | None | 26,486 | Search repeated using phrase instead, see below. |
| 20.01.2025 | Pre-defined keyword: “Police Cells” | Phrase search | 1 | None  Notes: The “Illicit Drug Use, Sources of Australian Data” report information from the National Police Custody Survey and Drug Use Monitoring in Australia, but there is nothing new requiring its inclusion. |
| 20.01.2025 | Pre-defined keyword: “Police Detainees” | Phrase search | 2 | None |
| 20.01.2025 | Pre-defined keyword: “Custodial Care” | Phrase search | 0 | None |
| 20.01.2025 | Pre-defined keyword: “Police Detention” | Phrase search | 0 | None |
| 20.01.2025 | Pre-defined keyword: “Policing” | Phrase search | 76 | None |
| 20.01.2025 | Pre-defined keyword: “Offenders” | Phrase search | 294 | None  Notes: Individuals in police custody are also excluded from the “Corrective Services, Australia” reports. |
| 22/01/2025 | Pre-defined keyword: “Watchhouse” | Phrase search | 1 | None |
| 22/01/2025 | Pre-defined keyword: “Custody Suite” | Phrase search | 0 | None |
| 22/01/2025 | Pre-defined keyword:  “Short term custody” | Phrase search | 0 | None |
| 22/01/2025 | Pre-defined keyword:  “Police watchhouse” | Phrase search | 0 | None |

**Australian Institute of Health and Welfare**

Website: [www.aihw.gov.au](http://www.aihw.gov.au)

| **Search Date** | **Search Term/s** | **Limits / Filters** | **Number of Results** | **Relevant studies/reports reviewed at full text and referred for second review** |
| --- | --- | --- | --- | --- |
| 22/01/2025 | Pre-defined keyword:  Police custody | Content type: “Everything” | 33 | None |
| 22/01/2025 | Pre-defined keyword:  Police cells | Content type: “Everything” | 19 | None |
| 22/01/2025 | Pre-defined keyword:  Police detainees | Content type: “Everything” | 16 | None |
| 22/01/2025 | Pre-defined keyword:  Custodial care | Content type: “Everything” | 68 | None |
| 22/01/2025 | Pre-defined keyword:  Police detention | Content type: “Everything” | 30 | None |
| 22/01/2025 | Pre-defined keyword:  Policing | Content type: “Releases and Reports” | 19 | None |
| 22/01/2025 | Pre-defined keyword:  Offenders | Content type: “Releases and Reports” | 14 | None |
| 22/01/2025 | Pre-defined keyword:  Watchhouse | Content type: “Everything” | 0 | None |
| 22/01/2025 | Pre-defined keyword:  Custody suite | Content type: “Everything” | 7 | None |
| 22/01/2025 | Pre-defined keyword:  Short term custody | Content type: “Everything” | 94 | None |
| 22/01/2025 | Pre-defined keyword:  Police watchhouse | Content type: “Everything” | 0 | None |

**Australian Institute of Criminology**

Website: [www.aic.gov.au](http://www.aic.gov.au)

Documents on the AIC website can be viewed by publication series. All documents organized into publication series were reviewed:

| **Date** | **Pages Browsed** | **Relevant studies/reports reviewed at full text and referred for second review** |
| --- | --- | --- |
| 22.01.2025 | Publications: Special Reports | 21 reviewed, 0 referred |
| 22.01.2025 | Publications: Trends & issues in crime and criminal justice, result pages 1-57 (until year 2000) | 563 reviewed, 0 referred, all that appeared eligible were already captured by systematic searches. |
| 22.01.2025 | Publications by series: Violence Today | 9 results, 0 referred |
| 22.01.2025 | Publications by series: Violence Prevention Today | 2 results, 0 referred |
| 22.01.2025 | Publications by series: Transnational Crime Brief | 6 results, 0 referred |
| 22.01.2025 | Publications by series: Training Project Reports | 14 results, 0 referred |
| 22.01.2025 | Publications by series: Training Project Proceedings | 21 results, 0 referred |
| 22.01.2025 | Publications by series: Technical and Background Paper Series | 57 results, 0 referred |
| 22.01.2025 | Publications by series:  Statistical Report | 50 results, 7 referred:  5. Drug use monitoring in Australia: Drug use among police detainees, 2019  6. Drug use monitoring in Australia: Drug use among police detainees, 2020  7. Drug use monitoring in Australia: Drug use among police detainees, 2021 |
| 22.01.2025 | Publications by series:  Statistical Bulletin | 46 results, 0 referred |
| 22.01.2025 | Publications by series: Special Reports | 21 results, 0 referred |
| 22.01.2025 | Publications by series: Seminar Proceedings Series | 27 results, 0 referred |
| 22.01.2025 | Publications by series: Review of Australian Criminology Research | 4 results, 0 referred |
| 22.01.2025 | Publications by series: Research Report | 33 results, 0 referred |
| 22.01.2025 | Publications by series: Research in Practice | 48 results, 2 referred:  8. Alcohol and disorderly conduct on Friday and Saturday nights: Findings from the DUMA program  9. Victimisation and fear of crime among a sample of police detainees |
| 22.01.2025 | Publications by series: Research and Public Policy Series | 131 results, 0 referred |
| 22.01.2025 | Publications by series: NDLEARF Research Summary | 29 results, 0 referred |
| 22.01.2025 | Publications by series: NDLEARF Research Bulletin | 7 results, 0 referred |
| 22.01.2025 | Publications by series: NDLEARF Reports and Discussion Papers | 6 results, 0 referred |
| 22.01.2025 | Publications by series: NDLEARF Monograph | 70 results, 0 referred |
| 22.01.2025 | Publications by series: National Committee on Violence Monograph Series | 4 results, 0 referred |
| 22.01.2025 | Publications by series: Monitoring Reports | 27 results, 0 referred |
| 22.01.2025 | Publications by series: High tech crime brief | 17 results, 0 referred |
| 22.01.2025 | Publications by series: Event proceedings and reports | 14 results, 0 referred |
| 22.01.2025 | Publications by series: Deaths in Custody Australia | 16 results, 0 referred (all documents in this category were pre-2000) |
| 22.01.2025 | Publications by series: Crime Prevention Series | 11 results, 0 referred |
| 22.01.2025 | Publications by series: Crime Facts Info | 196 results, 0 referred  Note: all documents in this series are fact sheets which reproduce one figure or section from a recent publication. |
| 22.01.2025 | Publications by series: Conference Proceedings Series | 27 results, 0 referred |
| 22.01.2025 | Publications by series: Australian Studies in Law, Crime and Justice | 9 results, 0 referred |
| 22.01.2025 | Publications by series: Australia Crime Facts and Figures | 17 results, 0 referred  Note: selected issues of *Australian Crime: Facts & Figures* included a section summarising the profile of the police custody population, however these figures were reproduced from DUMA and National Police Custody Survey reports. |
| 22.01.2025 | Publications by series: Archival Series | 8 results, 0 referred |
| 22.01.2025 | Publications by series: Archive | 168 results, 0 referred |
| 22.01.2025 | Publications by series: Annual Report | 29 results, 0 referred |
| 22.01.2025 | Publications by series: AI Crime Reduction Matters | 81 results, 0 referred |

## Grey Literature Searches: Australian Capital Territory Websites

**ACT Policing**

Website: [www.police.act.gov.au](http://www.police.act.gov.au)

| **Search Date** | **Search Term/s** | **Limits / Filters** | **Number of Results** | **Relevant studies/reports reviewed at full text and referred for second review** |
| --- | --- | --- | --- | --- |
| 28.01.2025 | Pre-defined keyword:  Health | None | 34 | None |
| 28.01.2025 | Pre-defined keyword:  Mental health | None | 48 | None |
| 28.01.2025 | Pre-defined keyword:  Medical care | None | 46 | None |
| 28.01.2025 | Pre-defined keyword:  Treatment | None | 34 | None |
| 28.01.2025 | Pre-defined keyword:  Medication | None | 5 | None |
| 28.01.2025 | Pre-defined keyword:  Disease | None | 3 | None |
| 28.01.2025 | Pre-defined keyword:  Diagnosis | None | 3 | None |
| 28.01.2025 | Pre-defined keyword:  Morbidity | None | 0 | None |
| 28.01.2025 | Pre-defined keyword:  Mortality | None | 0 | None |
| 28.01.2025 | Pre-defined keyword:  Disability | None | 29 | None |
| 28.01.2025 | Pre-defined keyword:  Prevalence | None | 1 | None |
| 28.01.2025 | Pre-defined keyword:  Incidence | None | 6 | None |
| 28.01.2025 | Pre-defined keyword:  Watchhouse | None | 0 | None |
| 28.01.2025 | Iterative search: health in custody | None | 51 | None |
| 28.01.2025 | Iterative search: health police custody | None | 49 | None |

**ACT Government – Justice and Community Safety Directorate**

Website: <https://www.act.gov.au>

| **Search Date** | **Search Term/s** | **Limits / Filters** | **Number of Results** | **Relevant studies/reports reviewed at full text and referred for second review** |
| --- | --- | --- | --- | --- |
| 28.01.2025 | Pre-defined keyword: police custody | Searching Justice and Community Safety Directorate only | 15 | None |
| 28.01.2025 | Pre-defined keyword:  Health | Searching Justice and Community Safety Directorate only | 12 | None |
| 28.01.2025 | Pre-defined keyword:  Mental health | Searching Justice and Community Safety Directorate only | 13 | None |
| 28.01.2025 | Pre-defined keyword:  Medical care | Searching Justice and Community Safety Directorate only | 15 | None |
| 28.01.2025 | Pre-defined keyword:  Treatment | Searching Justice and Community Safety Directorate only | 7 | None |
| 28.01.2025 | Pre-defined keyword:  Medication | Searching Justice and Community Safety Directorate only | 1 | None |
| 28.01.2025 | Pre-defined keyword:  Disease | Searching Justice and Community Safety Directorate only | 0 | None |
| 28.01.2025 | Pre-defined keyword:  Diagnosis | Searching Justice and Community Safety Directorate only | 0 | None |
| 28.01.2025 | Pre-defined keyword:  Morbidity | Searching Justice and Community Safety Directorate only | 0 | None |
| 28.01.2025 | Pre-defined keyword:  Mortality | Searching Justice and Community Safety Directorate only | 0 | None |
| 28.01.2025 | Pre-defined keyword:  Disability | Searching Justice and Community Safety Directorate only | 10 | None |
| 28.01.2025 | Pre-defined keyword:  Prevalence | Searching Justice and Community Safety Directorate only | 0 | None |
| 28.01.2025 | Pre-defined keyword:  Incidence | Searching Justice and Community Safety Directorate only | 0 | None |

**ACT Government – ACT Health**

Website: <https://www.act.gov.au>

| **Search Date** | **Search Term/s** | **Limits / Filters** | **Number of Results** | **Relevant studies/reports reviewed at full text and referred for second review** |
| --- | --- | --- | --- | --- |
| 28.01.2025 | Pre-defined keyword: police custody | Searching ACT Health directorate only | 24 | None |
| 29.01.2025 | Pre-defined keyword:  Police cells | Searching ACT Health directorate only | 25 | None |
| 29.01.2025 | Pre-defined keyword:  Police detainees | Searching ACT Health directorate only | 26 | None |
| 29.01.2025 | Pre-defined keyword:  Custodial care | Searching ACT Health directorate only | 247 | None |
| 29.01.2025 | Pre-defined keyword:  Police detention | Searching ACT Health directorate only | 30 | None |
| 29.01.2025 | Pre-defined keyword:  Policing | Searching ACT Health directorate only | 5 | None |
| 29.01.2025 | Pre-defined keyword:  Offenders | Searching ACT Health directorate only | 0 | None |
| 29.01.2025 | Pre-defined keyword:  Watchhouse | Searching ACT Health directorate only | 0 | None |
| 29.01.2025 | Pre-defined keyword:  Custody suite | Searching ACT Health directorate only | 11 | None |
| 29.01.2025 | Pre-defined keyword:  Short term custody | Searching ACT Health directorate only | 20 | None |
| 29.01.2025 | Pre-defined keyword:  Police watchhouse | Searching ACT Health directorate only | 21 | None |

Other pages browsed:

| **Date** | **Pages Browsed** | **Relevant studies referred for second screen** |
| --- | --- | --- |
| 29.01.2025 | Home > Directorates and agencies > ACT Health > Data, statistics and surveys >  HealthStats ACT >  Epidemiology > publications | None |
| 29.01.2025 | Home > Directorates and agencies > ACT Health > Data, statistics and surveys >  HealthStats ACT >  Data Collections | None |

**ACT Ombudsman**

Website: [www.ombudsman.act.gov.au](http://www.ombudsman.act.gov.au)

The search function has four types of broad outputs to filter by: All, Reports, Resources, Media Releases, Translated Information. Different combinations of searching either “All” documents or “reports” were used in combination with other filters.

| **Search Date** | **Search Term/s** | **Limits / Filters** | **Number of Results** | **Relevant studies/reports reviewed at full text and referred for second review** |
| --- | --- | --- | --- | --- |
| 28.01.2025 | Pre-defined keyword:  Police custody | Searching Reports | 33 | None |
| 28.01.2025 | Pre-defined keyword:  Police cells | Searching Reports | 32 | None |
| 28.01.2025 | Pre-defined keyword:  Police detainees | Searching Reports | 38 | None |
|  | Pre-defined keyword:  Police detainees | Searching All, filtered to Investigation Reports | 33 | None |
| 28.01.2025 | Pre-defined keyword:  Custodial care | Searching Reports | 32 | None |
| 28.01.2025 | Pre-defined keyword:  Custodial care | Searching All, filtered to Investigation Reports | 24 | None |
| 28.01.2025 | Pre-defined keyword:  Police detention | Searching All, filtered to Investigation Reports | 32 | None |
| 28.01.2025 | Pre-defined keyword:  Watchhouse | Searching All, no filters | 26 | None |

Additional Browsing completed on ACT Ombudsman website:

| **Date** | **Pages Browsed** | **Relevant studies/reports reviewed at full text and referred for second review** |
| --- | --- | --- |
| 28.01.2025 | Publications, refined by “Investigation Reports” | None. Multiple Ombudsman reports identified through systematic searches also appeared here. |
| 28.01.2025 | Publications, refined by “Reports” or “Report” | None |

## Grey Literature Searches: New South Wales Websites

**NSW Police**

Website: [www.police.nsw.gov.au](http://www.police.nsw.gov.au)

Notes:

- The search function on NSW Police Website does not allow the use of filters
- For searches that return more than 100 results, only the first 100 are viewable. For these searches, all viewable results were reviewed.

| **Search Date** | **Search Term/s** | **Limits / Filters** | **Number of Results** | **Relevant studies/reports reviewed at full text and referred for second review** |
| --- | --- | --- | --- | --- |
| 29.01.2025 | Pre-defined keyword:  Health | None | 860 | None |
| 29.01.2025 | Pre-defined keyword:  Mental health | None | 294 | Results broad, including pages that only included “Health”. Search revised, see below row. |
| 29.01.2025 | Iterative Search:  “Mental health” | None | 213 | None |
| 29.01.2025 | Pre-defined keyword:  “Medical care” | None | 8 | None |
| 29.01.2025 | Pre-defined keyword:  Treatment | None | 269 | None |
| 4.02.2025 | Pre-defined keyword:  Medication | None | 107 | None |
| 4.02.2025 | Pre-defined keyword:  Disease | None | 71 | None |
| 4.02.2025 | Pre-defined keyword:  Diagnosis | None | 32 | None |
| 4.02.2025 | Pre-defined keyword:  Morbidity | None | 0 | None |
| 4.02.2025 | Pre-defined keyword:  Mortality | None | 1 | None |

Additional pages browsed:

| **Date** | **Pages Browsed** | **Relevant studies/reports reviewed at full text and referred for second review** |
| --- | --- | --- |
| 4.02.2025 | Home > About us >  Publications and Corporate Information | None – nil in annual reports on the details of police detainees. |

**Department of Communities and Justice - NSW Government**

Website: [www.justice.nsw.gov.au](http://www.justice.nsw.gov.au)

Notes:

- The Department of Justice, NSW Government website does not have the ability to use filters to narrow results. In the event that searches returned more than 200 results, only the first 200 were reviewed.

| **Search Date** | **Search Term/s** | **Limits / Filters** | **Number of Results** | **Relevant studies/reports reviewed at full text and referred for second review** |
| --- | --- | --- | --- | --- |
| 29.01.2025 | Pre-defined keyword:  Health | None | 2624 | None in the first 200. |
| 29.01.2025 | Pre-defined keyword:  Mental health | None | 2359 | None in the first 200. |
| 29.01.2025 | Pre-defined keyword:  Medical care | None | 1987 | None in the first 200. |
| 29.01.2025 | Pre-defined keyword:  Mortality | None | 73 | None |
| 29.01.2025 | Pre-defined keyword:  Prevalence | None | 257 | None in the first 200. |
| 29.01.2025 | Pre-defined keyword:  Incidence | None | 212 | None in the first 200. |
| 29.01.2025 | Pre-defined keyword:  Police cells | None | 1087 | None in the first 200. |
| 29.01.2025 | Pre-defined keyword:  Watchhouse | None | 0 | None |
| 29.01.2025 | Pre-defined keyword:  Police detainees | None | 1069 | None in the first 200. |

Additional pages browsed:

| **Date** | **Pages Browsed** | **Relevant studies/reports reviewed at full text and referred for second review** |
| --- | --- | --- |
| 29.01.2025 | Home > About us > Family and Community Services Insights, Analysis and Research (FACSIAR) > Research partnerships > **Our research partners and projects** | None |
| 29.01.2025 | Home > About us > Family and Community Services Insights, Analysis and Research (FACSIAR) > Research partnerships > **Our publications and resources** | None |
| 29.01.2025 | Home > About us > Family and Community Services Insights, Analysis and Research (FACSIAR) > Evidence hub > **Evidence reviews and summaries** | None |
| 29.01.2025 | Home > About us > Family and Community Services Insights, Analysis and Research (FACSIAR) > Evidence hub > **Evidence 'how to' guides** | None |
| 29.01.2025 | Home > About us > Family and Community Services Insights, Analysis and Research (FACSIAR) > Evidence hub > **Evaluation repository** | None |
| 29.01.2025 | Home > Legal and justice | None |

**NSW Bureau of Crime Statistics and Research**

Website: [www.bocsar.nsw.gov.au](http://www.bocsar.nsw.gov.au)

Notes:

- When using the general search function, no limits or filters are able to be used. When using the general search function, if more than 200 results were generated, only the first 200 were reviewed.
- The Publications & Evaluations page also contained a separate search function. This was used for some searches.

| **Search Date** | **Search Term/s** | **Limits / Filters** | **Number of Results** | **Relevant studies/reports reviewed at full text and referred for second review** |
| --- | --- | --- | --- | --- |
| 29.01.2025 | Pre-defined keyword:  Police custody | General Search Function, no filters or limitations | 1709 | None in the first 200. |
| 29.01.2025 | Pre-defined keyword:  Police detainees | General Search Function, no filters or limitations | 1466 | None in the first 200. |
| 29.01.2025 | Pre-defined keyword:  Health | General Search Function, no filters or limitations | 699 | None in the first 200. |
| 29.01.2025 | Pre-defined keyword:  Mental health | General Search Function, no filters or limitations | 715 | None in the first 200. |
| 29.01.2025 | Pre-defined keyword:  Medical care | General Search Function, no filters or limitations | 302 | None in the first 200. |
| 29.01.2025 | Pre-defined keyword:  Morbidity | General Search Function, no filters or limitations | 24 | None |
| 29.01.2025 | Pre-defined keyword:  Mortality | General Search Function, no filters or limitations | 34 | None |
| 29.01.2025 | Pre-defined keyword:  Police custody | Publications & Evaluations search function, no limitations or filters | 21 | None |
| 29.01.2025 | Pre-defined keyword:  Police detainees | Publications & Evaluations search function, no limitations or filters | 6 | None |
| 29.01.2025 | Pre-defined keyword:  Police cells | Publications & Evaluations search function, no limitations or filters | 0 | None |
| 29.01.2025 | Pre-defined keyword:  Police detention | Publications & Evaluations search function, no limitations or filters | 7 | None |

Additional pages browsed:

| **Date** | **Pages Browsed** | **Relevant studies/reports reviewed at full text and referred for second review** |
| --- | --- | --- |
| 29.01.2025 | Home > Statistics & dashboards > Custody > **Custody receptions and discharges** | None |
| 29.01.2025 | Home > Statistics & dashboards > Custody > **Custody Dashboard** | None |

**NSW Ombudsman**

Website: [www.ombo.nsw.gov.au](http://www.ombo.nsw.gov.au)

Notes:

- Searches were completed both using the general search function and using the Reports page search function.

| **Search Date** | **Search Term/s** | **Limits / Filters** | **Number of Results** | **Relevant studies/reports reviewed at full text and referred for second review** |
| --- | --- | --- | --- | --- |
| 29.01.2025 | Pre-defined keyword:  Police custody | None | 1 | None |
| 29.01.2025 | Pre-defined keyword:  Police cells | None | 0 | None |
| 29.01.2025 | Pre-defined keyword:  Police detainees | None | 1 | None |
| 29.01.2025 | Pre-defined keyword:  Custodial care | None | 0 | None |
| 29.01.2025 | Pre-defined keyword:  Police detention | None | 13 | None |
| 29.01.2025 | Pre-defined keyword:  Policing | None | 10 | None |
| 29.01.2025 | Pre-defined keyword:  Offenders | None | 26 | None |
| 29.01.2025 | Pre-defined keyword:  Watchhouse | None | 0 | None |
| 29.01.2025 | Pre-defined keyword:  Custody suite | None | 0 | None |
| 29.01.2025 | Pre-defined keyword:  Short term custody | None | 0 | None |
| 29.01.2025 | Pre-defined keyword:  Police watchhouse | None | 0 | None |

Additional pages browsed:

| **Date** | **Pages Browsed** | **Relevant studies/reports reviewed at full text and referred for second review** |
| --- | --- | --- |
| 29.01.2025 | Home > Reports > Research reports (14 results) | None |
| 29.01.2025 | Home > Reports > Reports to Parliament - Filtered by selecting “Police” option (68 results) | None |
| 29.01.2025 | Home > Reports > Reports to Parliament - Filtered by selecting “Juvenile Justice” option (1 result) | None |
| 29.01.2025 | Home > Reports > Reports to Parliament - Filtered by selecting “Law Enforcement” option (79 result) | None |
| 29.01.2025 | Home > Reports > Reports to Parliament - Filtered by selecting “Correctional centre” option (3 result) | None |
| 29.01.2025 | Home > Reports > Reports to Parliament - Filtered by selecting “Aboriginal” option (11 result) | None |
| 29.01.2025 | Home > Reports (all) – Filtered by searching “police custody” (1 result) | None |

**Law Enforcement Conduct Commission**

Website: [www.lecc.nsw.gov.au](http://www.lecc.nsw.gov.au)

| **Search Date** | **Search Term/s** | **Limits / Filters** | **Number of Results** | **Relevant studies/reports reviewed at full text and referred for second review** |
| --- | --- | --- | --- | --- |
| 04.02.2025 | Pre-defined keyword:  Health | None | 61 | None |
| 04.02.2025 | Pre-defined keyword:  Mental health | None | 73 | None |
| 04.02.2025 | Pre-defined keyword:  Medical care | None | 89 | None |
| 04.02.2025 | Pre-defined keyword:  Treatment | None | 52 | None |
| 04.02.2025 | Pre-defined keyword:  Medication | None | 6 | None |
| 04.02.2025 | Pre-defined keyword:  Disease | None | 11 | None |
| 04.02.2025 | Pre-defined keyword:  Diagnosis | None | 2 | None |
| 04.02.2025 | Pre-defined keyword:  Morbidity | None | 1 | None |
| 04.02.2025 | Pre-defined keyword:  Mortality | None | 1 | None |
| 04.02.2025 | Pre-defined keyword:  Disability | None | 25 | None |
| 04.02.2025 | Pre-defined keyword:  Prevalence | None | 11 | None |
| 04.02.2025 | Pre-defined keyword:  Incidence | None | 10 | None |

Additional pages browsed:

| **Date** | **Pages Browsed** | **Relevant studies/reports reviewed at full text and referred for second review** |
| --- | --- | --- |
| 05.02.2025 | <https://www.lecc.nsw.gov.au/index-of-operations-and-published-reports> | Reviewed all published reports from 2018 – 2015. One report referred for 2^nd^ review:  10. Operation Kimbla - Final Report |

**NSW Health**

Website: [www.health.nsw.gov.au](http://www.health.nsw.gov.au)

- When using the general search function on the NSW health website, no limits or filters are able to be used and only the first 100 results are able to be viewed.

| **Search Date** | **Search Term/s** | **Limits / Filters** | **Number of Results** | **Relevant studies/reports reviewed at full text and referred for second review** |
| --- | --- | --- | --- | --- |
| 05.02.2025 | Pre-defined keyword:  Police custody | None | 1,370 | None in the first 100 results |
| 05.02.2025 | Pre-defined keyword:  Police cells | None | 538 | None in the first 100 results |
| 05.02.2025 | Pre-defined keyword:  Police detainees | None | 63 | None |
| 05.02.2025 | Pre-defined keyword:  Custodial care | None | 561 | None in the first 100 results |
| 05.02.2025 | Pre-defined keyword:  Police detention | None | 1,300 | None in the first 100 results |
| 05.02.2025 | Pre-defined keyword:  Policing | None | 131 | None in the first 100 results |
| 05.02.2025 | Pre-defined keyword:  Offenders | None | 632 | None in the first 100 results |
| 05.02.2025 | Pre-defined keyword:  Watchhouse | None | 1 | None |
| 05.02.2025 | Pre-defined keyword:  Custody suite | None | 1,150 | None in the first 100 results |

Additional pages browsed:

| **Date** | **Pages Browsed** | **Relevant studies/reports reviewed at full text and referred for second review** |
| --- | --- | --- |
| 06.02.2025 | Home > Publications and resources > Reports | None |
| 06.02.2025 | Home > Research | None, but identified Justice Health NSW, and Health Stat NSW as other potential website to search. Added to the list. |
| 06.02.2025 | Home > Open data | None |
| 06.02.2025 | Home > Open data > Aboriginal health data | None |
| 06.02.2025 | Home > Population health research and evaluation | None |
| 06.02.2025 | Home > Population health research and evaluation > Evaluation Reports | None |
| 06.02.2025 | Home > Population health research and evaluation > Economic Evaluation | None |
| 06.02.2025 | Home > Population health research and evaluation > Program Evaluation | None |
| 06.02.2025 | Home > Population health research and evaluation > Online Presentations | None |
| 06.02.2025 | Home > Population health research and evaluation > Peer-reviewed publications | None |
| 06.02.2025 | Home > Epidemiology and Evidence | None |

**HealthStats NSW**

Website: <https://www.healthstats.nsw.gov.au/home>

| **Search Date** | **Search Term/s** | **Limits / Filters** | **Number of Results** | **Relevant studies/reports reviewed at full text and referred for second review** |
| --- | --- | --- | --- | --- |
| 06.02.2025 | Pre-defined keyword:  Police custody | None | 0 | None |
| 06.02.2025 | Pre-defined keyword:  Police cells | None | 1 | None |
| 06.02.2025 | Pre-defined keyword:  Police detainees | None | 0 | None |
| 06.02.2025 | Iterative search: Police | None | 0 | None |
| 06.02.2025 | Iterative search: Custody | None | 0 | None |
| 06.02.2025 | Pre-defined keyword:  Policing | None | 0 | None |
| 06.02.2025 | Pre-defined keyword:  Offenders | None | 0 | None |
| 06.02.2025 | Pre-defined keyword:  Watchhouse | None | 0 | None |
| 06.02.2025 | Pre-defined keyword:  Custody suite | None | 1 | None |

Additional pages browsed:

| **Date** | **Pages Browsed** | **Relevant studies/reports reviewed at full text and referred for second review** |
| --- | --- | --- |
| 06.02.2025 | Topics | None |
| 06.02.2025 | HealthStats PLUS | None |
| 06.02.2025 | HealthStats PLUS > Data Reports | None |
| 06.02.2025 | HealthStats PLUS > Archived Data Reports | None |

**Justice Health and Forensic Mental Health Network NSW**

Website: [www.nsw.gov.au/health/justicehealth](http://www.nsw.gov.au/health/justicehealth)

No discrete search function. All searches are completed for all of NSW Government. Instead page was browsed for useful documents or reports.

| **Date** | **Pages Browsed** | **Relevant studies/reports reviewed at full text and referred for second review** |
| --- | --- | --- |
| 06.02.2025 | Home > Health > Justice Health NSW > Our Research | None |
| 06.02.2025 | Home > Health > Justice Health NSW > Our Research > Research Projects | None |

## Grey Literature Searches: Northern Territory Websites

**NT Police Force**

Website: [www.pfes.nt.gov.au/police](http://www.pfes.nt.gov.au/police)

- The search function on the NT Police website searches the entire PFES website – covering police, fire and emergency services websites.

| **Search Date** | **Search Term/s** | **Limits / Filters** | **Number of Results** | **Relevant studies/reports reviewed at full text and referred for second review** |
| --- | --- | --- | --- | --- |
| 06.02.2025 | Pre-defined keyword:  Health | None | 73 | None |
| 06.02.2025 | Pre-defined keyword:  Mental health | None | 8 | None |
| 06.02.2025 | Pre-defined keyword:  Medical care | None | 21 | None |
| 06.02.2025 | Pre-defined keyword:  Treatment | None | 2 | None |
| 06.02.2025 | Pre-defined keyword:  Medication | None | 36 | None |
| 06.02.2025 | Pre-defined keyword:  Disease | None | 1 | None |
| 06.02.2025 | Pre-defined keyword:  Diagnosis | None | 0 | None |
| 06.02.2025 | Pre-defined keyword:  Morbidity | None | 0 | None |
| 06.02.2025 | Pre-defined keyword:  Mortality | None | 0 | None |
| 06.02.2025 | Pre-defined keyword:  Disability | None | 9 | None |
| 06.02.2025 | Pre-defined keyword:  Prevalence | None | 3 | None |
| 06.02.2025 | Pre-defined keyword:  Incidence | None | 38 | None |

Additional pages browsed:

| **Date** | **Pages Browsed** | **Relevant studies/reports reviewed at full text and referred for second review** |
| --- | --- | --- |
| 06.02.2025 | Home > Choose a service area > Police > Publications | None |

**NT Attorney-General’s Department**

Website: [www.justice.nt.gov.au](http://www.justice.nt.gov.au)

- Searches completed on the NT Department of the Attorney-General and Justice website search all of the NT government website and there are no filter options. As a result, the pre-defined search terms returned extremely broad search results, that were not specific to the website of interest. After a few initial searches, iterative searches were completed.

| **Search Date** | **Search Term/s** | **Limits / Filters** | **Number of Results** | **Relevant studies/reports reviewed at full text and referred for second review** |
| --- | --- | --- | --- | --- |
| 06.02.2025 | Pre-defined keyword:  Health | None | 1,795 | None in the first 100 |
| 06.02.2025 | Iterative search: Health in police custody | None | 197 | None in the first 100 |
| 06.02.2025 | Iterative search: Health in police cells | None | 210 | None in the first 100 |
| 06.02.2025 | Iterative search: police detainee health | None | 177 | None in the first 100. |
| 06.02.2025 | Pre-defined keyword:  Police watchhouse | None | 529 | None in the first 100. |

Additional pages browsed:

| **Date** | **Pages Browsed** | **Relevant studies/reports reviewed at full text and referred for second review** |
| --- | --- | --- |
| 06.02.2025 | Home > Our divisions > Financial Management Group (FMG) >  Program Evaluation Unit | Reviewed all published evaluations – nil eligible. |
| 06.02.2025 | Home > Attorney-General and Justice > Justice publications | None |
| 06.02.2025 | Home > Attorney-General and Justice > Justice publications > Registrar-General’s Directions | None |
| 06.02.2025 | Home > Attorney-General and Justice > Justice publications > other publications | None |
| 06.02.2025 | Home > Attorney-General and Justice > Justice publications > Hamburger Report | None |
| 06.02.2025 | Home > Attorney-General and Justice > Justice publications > Annual Reports | None |

**Ombudsman Northern Territory**

Website: [www.ombudsman.nt.gov.au](http://www.ombudsman.nt.gov.au)

| **Search Date** | **Search Term/s** | **Limits / Filters** | **Number of Results** | **Relevant studies/reports reviewed at full text and referred for second review** |
| --- | --- | --- | --- | --- |
| 06.02.2025 | Pre-defined keyword:  Police custody | None | 19 | 11. Extraordinary Restraint: Spit Hood & Emergency Restraint Chair Use on Children in Police Custody |
| 06.02.2025 | Pre-defined keyword:  Police cells | None | 19 | None |
| 06.02.2025 | Pre-defined keyword:  Police detainees | None | 19 | None |
| 06.02.2025 | Pre-defined keyword:  Custodial care | None | 0 | None |
| 06.02.2025 | Pre-defined keyword:  Police detention | None | 21 | None |
| 06.02.2025 | Pre-defined keyword:  Policing | None | 0 | None |
| 06.02.2025 | Pre-defined keyword:  Offenders | None | 0 | None |
| 06.02.2025 | Pre-defined keyword:  Watchhouse | None | 0 | None |
| 06.02.2025 | Pre-defined keyword:  Custody suite | None | 2 | None |
| 06.02.2025 | Pre-defined keyword:  Short term custody | None | 4 | None |
| 06.02.2025 | Pre-defined keyword:  Police watchhouse | None | 19 | None |

Additional pages browsed:

| **Date** | **Pages Browsed** | **Relevant studies/reports reviewed at full text and referred for second review** |
| --- | --- | --- |
| 06.02.2025 | Home > News and Publications | Refined page by looking at “publications” only, and excluding “news”  Nil further documents identified. |
| 06.02.2025 | Home > OPCAT | None. |

**NT Health**

Website: [www.health.nt.gov.au](http://www.health.nt.gov.au)

- Searches completed on the NT Health website search all of the NT government website and there are no filter options. As a result, the pre-defined search terms returned extremely broad search results, that were not specific to the website of interest. A few iterative searches were completed, but these returned the same results as documented above for the Department of the Attorney-General and Justice website.

| **Search Date** | **Search Term/s** | **Limits / Filters** | **Number of Results** | **Relevant studies/reports reviewed at full text and referred for second review** |
| --- | --- | --- | --- | --- |
| 06.02.2025 | Pre-defined keyword:  Police custody | None | 560 | None in the first 100 |
| 06.02.2025 | Pre-defined keyword:  Watchhouse | None | 1 | None |

Additional pages browsed:

| **Date** | **Pages Browsed** | **Relevant studies/reports reviewed at full text and referred for second review** |
| --- | --- | --- |
| 06.02.2025 | Home > Research and Health data > Health Statistics and Informatics | Reviewed all 101 publications, nil referred for 2^nd^ review. |
| 06.02.2025 | Home > Research and Health data > Health research | None |
| 06.02.2025 | Home > Research and Health data > Health Data | None |

## Grey Literature Searches: Queensland Websites

**Crime and Corruption Commission Queensland**

Website: [www.ccc.qld.gov.au](http://www.ccc.qld.gov.au)

| **Search Date** | **Search Term/s** | **Limits / Filters** | **Number of Results** | **Relevant studies/reports reviewed at full text and referred for second review** |
| --- | --- | --- | --- | --- |
| 06.02.2025 | Pre-defined keyword:  Health | Excluded News Articles | 12 | None |
| 06.02.2025 | Pre-defined keyword:  Mental health | Excluded News Articles | 0 | None |
| 06.02.2025 | Pre-defined keyword:  Medical care | Excluded News Articles | 0 | None |
| 06.02.2025 | Pre-defined keyword:  Treatment | Excluded News Articles | 4 | None |
| 06.02.2025 | Pre-defined keyword:  Medication | Excluded News Articles | 0 | None |
| 06.02.2025 | Pre-defined keyword:  Disease | Excluded News Articles | 0 | None |
| 06.02.2025 | Pre-defined keyword:  Diagnosis | Excluded News Articles | 0 | None |
| 06.02.2025 | Pre-defined keyword:  Morbidity | Excluded News Articles | 0 | None |
| 06.02.2025 | Pre-defined keyword:  Mortality | Excluded News Articles | 0 | None |
| 06.02.2025 | Pre-defined keyword:  Disability | Excluded News Articles | 0 | None |
| 06.02.2025 | Pre-defined keyword:  Prevalence | Excluded News Articles | 5 | None |
| 06.02.2025 | Pre-defined keyword:  Incidence | Excluded News Articles | 6 | None |
| 06.02.2025 | Pre-defined keyword:  Police Custody | Excluded News Articles | 0 | None |

Additional pages browsed:

| **Date** | **Pages Browsed** | **Relevant studies/reports reviewed at full text and referred for second review** |
| --- | --- | --- |
| 06.02.2025 | CCC - Crime and Corruption Commission Queensland > Publications and News > Publications Filtered by tag: Police | Reviewed 69 results. None referred for 2^nd^ review. |

**Queensland Police Service**

Website: [www.police.qld.gov.au](http://www.police.qld.gov.au)

- When using the general search function on the Queensland Police website, no limits or filters can be used so only the first 100 results were reviewed.

| **Search Date** | **Search Term/s** | **Limits / Filters** | **Number of Results** | **Relevant studies/reports reviewed at full text and referred for second review** |
| --- | --- | --- | --- | --- |
| 06.02.2025 | Pre-defined keyword:  Health | None | 303 | None in the first 100 results |
| 06.02.2025 | Pre-defined keyword:  Mental health | None | 312 | None in the first 100 results |
| 06.02.2025 | Pre-defined keyword:  Medical care | None | 282 | None in the first 100 results |
| 06.02.2025 | Pre-defined keyword:  Treatment | None | 148 | None in the first 100 results |
| 06.02.2025 | Pre-defined keyword:  Medication | None | 38 | None |
| 06.02.2025 | Pre-defined keyword:  Disease | None | 46 | None |
| 06.02.2025 | Pre-defined keyword:  Diagnosis | None | 19 | None |
| 06.02.2025 | Pre-defined keyword:  Morbidity | None | 3 | None |
| 06.02.2025 | Pre-defined keyword:  Mortality | None | 3 | None |
| 06.02.2025 | Pre-defined keyword:  Disability | None | 134 | None in the first 100 |
| 06.02.2025 | Pre-defined keyword:  Prevalence | None | 26 | None |
| 06.02.2025 | Pre-defined keyword:  Incidence | None | 58 | None |

Additional pages browsed:

| **Date** | **Pages Browsed** | **Relevant studies/reports reviewed at full text and referred for second review** |
| --- | --- | --- |
| 06.02.2025 | Home > QPS corporate documents > Reports and publications | None |
| 06.02.2025 | Home > QPS corporate documents > Reports and publications > Watchhouse Data | Note: Queensland Police publish a daily report called “Persons in Queensland Police Watch-house Custody”. It contains the total number of adult and juvenile detainees in police watchhouse detention, the proportion that are male or female, and the proportion that identify as First Nations. It does not contain any specific health information, and you can only download that days report. |

**Department of Justice and Attorney-General**

Website: [www.justice.qld.gov.au](http://www.justice.qld.gov.au)

- When using the general search function on the Queensland Department of Justice and Attorney-General website, no limits or filters can be used so only the first 100 results were reviewed.

| **Search Date** | **Search Term/s** | **Limits / Filters** | **Number of Results** | **Relevant studies/reports reviewed at full text and referred for second review** |
| --- | --- | --- | --- | --- |
| 06.02.2025 | Pre-defined keyword:  Police custody | None | 380 | 1 study identified in the first 100:  12. Independent review into investigations of police-related deaths, and domestic and family violence deaths in Queensland . |
| 06.02.2025 | Iterative search:  Health in police custody | None | 126 | None in the first 100 |
| 06.02.2025 | Pre-defined keyword:  Police cells | None | 372 | None in the first 100 |
| 07.02.2025 | Pre-defined keyword:  Police detainees | None | 368 | None in the first 100 |
| 07.02.2025 | Pre-defined keyword:  Custodial care | None | 243 | None in the first 100 |
| 07.02.2025 | Pre-defined keyword:  Police detention | None | 398 | None in the first 100 |
| 07.02.2025 | Iterative search: health in police custody | None | 126 | None in the first 100 |
| 07.02.2025 | Pre-defined keyword:  Watchhouse | None | 1 | None |

Additional pages browsed:

| **Date** | **Pages Browsed** | **Relevant studies/reports reviewed at full text and referred for second review** |
| --- | --- | --- |
| 06.02.2025 | Home > Publications and policies > Reports | None |
| 06.02.2025 | Home > Projects and Initiatives | None |

**Queensland Ombudsman**

Website: [www.ombudsman.qld.gov.au](http://www.ombudsman.qld.gov.au)

| **Search Date** | **Search Term/s** | **Limits / Filters** | **Number of Results** | **Relevant studies/reports reviewed at full text and referred for second review** |
| --- | --- | --- | --- | --- |
| 07.02.2025 | Pre-defined keyword:  Police custody | None | 25 | 1 study identified:  13. Cairns and Murgon watch‑houses inspection report: Focus on detention of children |
| 07.02.2025 | Pre-defined keyword:  Police cells | None | 26 | None |
| 07.02.2025 | Pre-defined keyword:  Police detainees | None | 34 | None |
| 07.02.2025 | Pre-defined keyword:  Custodial care | None | 24 | None |
| 07.02.2025 | Pre-defined keyword:  Police detention | None | 66 | None |
| 07.02.2025 | Pre-defined keyword:  Watchhouse | None | 2 | None |
| 07.02.2025 | Pre-defined keyword:  Custody suite | None | 8 | None |

Additional pages browsed:

| **Date** | **Pages Browsed** | **Relevant studies/reports reviewed at full text and referred for second review** |
| --- | --- | --- |
| 07.02.2025 | Home > Publications > Detention inspection reports | None |
| 07.02.2025 | Home > Publications > Ombudsman investigative reports | None |
| 07.02.2025 | Home > Publications > Casebooks | None |
| 07.02.2025 | Home > Publications > Annual Reports | None |
| 07.02.2025 | Home > Detention inspection | None |

**Queensland Health**

Website: [www.health.qld.gov.au](http://www.health.qld.gov.au)

- When using the general search function on the Queensland Health website, no limits or filters can be used so only the first 100 results were reviewed.

| **Search Date** | **Search Term/s** | **Limits / Filters** | **Number of Results** | **Relevant studies/reports reviewed at full text and referred for second review** |
| --- | --- | --- | --- | --- |
| 07.02.2025 | Pre-defined keyword:  Police custody | None | 1191 | None in the first 100 |
| 07.02.2025 | Pre-defined keyword:  Police cells | None | 1109 | None in the first 100 |
| 07.02.2025 | Pre-defined keyword:  Police detainees | None | 954 | None in the first 100 |
| 07.02.2025 | Iterative search:  Custody | None | 449 | None in the first 100 |
| 07.02.2025 | Pre-defined keyword:  Watchhouse | None | 14 | None |

Additional pages browsed:

| **Date** | **Pages Browsed** | **Relevant studies/reports reviewed at full text and referred for second review** |
| --- | --- | --- |
| 07.02.2025 | Home > Research & reports > Reports > Public Health | None |
| 07.02.2025 | Home > Research & reports > Reviews and investigations | None |
| 07.02.2025 | Home > Research & reports > Reviews and investigations > Past reviews and investigations | None |
| 07.02.2025 | Home > Statistical services branch | None |

## Grey Literature Searches: South Australian Websites

**South Australia Police**

[www.police.sa.gov.au](http://www.police.sa.gov.au)

| **Search Date** | **Search Term/s** | **Limits / Filters** | **Number of Results** | **Relevant studies/reports reviewed at full text and referred for second review** |
| --- | --- | --- | --- | --- |
| 07.02.2025 | Pre-defined keyword:  Health | Excluded News and Careers pages | 51 | None |
| 07.02.2025 | Pre-defined keyword:  Mental health | Excluded News and Careers pages | 59 | None |
| 07.02.2025 | Pre-defined keyword:  Medical care | Excluded News and Careers pages | 57 | None |
| 07.02.2025 | Pre-defined keyword:  Treatment | Excluded News and Careers pages | 7 | None |
| 07.02.2025 | Pre-defined keyword:  Medication | Excluded News pages | 6 | None |
| 07.02.2025 | Pre-defined keyword:  Disease | None | 22 | None |
| 07.02.2025 | Iterative search: health police detainees | Only results including all three terms | 5 | None |
| 07.02.2025 | Pre-defined keyword:  Diagnosis | None | 8 | None |
| 07.02.2025 | Pre-defined keyword:  Morbidity | None | 2 | None |
| 07.02.2025 | Pre-defined keyword:  Mortality | None | 7 | None |
| 07.02.2025 | Pre-defined keyword:  Disability | Excluded News and Careers pages | 30 | None |

Additional pages browsed:

| **Date** | **Pages Browsed** | **Relevant studies/reports reviewed at full text and referred for second review** |
| --- | --- | --- |
| 07.02.2025 | Home > About us > Crime statistics | None |

**Attorney General’s Department**

[www.agd.sa.gov.au](http://www.agd.sa.gov.au)

- When using the general search function on the SA Attorney General Department website, no limits or filters can be used so only the first 100 results were reviewed.

| **Search Date** | **Search Term/s** | **Limits / Filters** | **Number of Results** | **Relevant studies/reports reviewed at full text and referred for second review** |
| --- | --- | --- | --- | --- |
| 07.02.2025 | Pre-defined keyword:  Health | None | 188 | None in the first 100 |
| 07.02.2025 | Iterative search: Health in police custody | Only results including all words | 41 | None |
| 07.02.2025 | Pre-defined keyword:  Mental health | Only results including all words | 55 | None |
| 07.02.2025 | Pre-defined keyword:  Medical care | Only results including all words | 57 | None |
| 07.02.2025 | Pre-defined keyword:  Watchhouse | None | 0 | None |
| 07.02.2025 | Pre-defined keyword:  Police cells | Only results including all words | 6 | None |
| 07.02.2025 | Pre-defined keyword:  Police detainees | Only results including all words | 2 | None |

Additional pages browsed:

| **Date** | **Pages Browsed** | **Relevant studies/reports reviewed at full text and referred for second review** |
| --- | --- | --- |
| 07.02.2025 | Home > Aboriginal Affairs and reconciliation | None |
| 07.02.2025 | Home > Aboriginal Affairs and reconciliation > Resources and Publications | None |
| 07.02.2025 | Home > Aboriginal affairs > Closing the Gap | None |

**Office of Public Integrity**

[www.publicintegrity.sa.gov.au](http://www.publicintegrity.sa.gov.au)

| **Search Date** | **Search Term/s** | **Limits / Filters** | **Number of Results** | **Relevant studies/reports reviewed at full text and referred for second review** |
| --- | --- | --- | --- | --- |
| 07.02.2025 | Pre-defined keyword:  Health | None | 7 | None |
| 07.02.2025 | Pre-defined keyword:  Mental health | None | 7 | None |
| 07.02.2025 | Pre-defined keyword:  Medical care | None | 3 | None |
| 07.02.2025 | Pre-defined keyword:  Treatment | None | 0 | None |
| 07.02.2025 | Pre-defined keyword:  Medication | None | 0 | None |
| 07.02.2025 | Pre-defined keyword:  Disease | None | 0 | None |
| 07.02.2025 | Pre-defined keyword:  Diagnosis | None | 0 | None |
| 07.02.2025 | Pre-defined keyword:  Morbidity | None | 0 | None |
| 07.02.2025 | Pre-defined keyword:  Mortality | None | 0 | None |
| 07.02.2025 | Pre-defined keyword:  Disability | None | 1 | None |
| 07.02.2025 | Iterative search: health in police custody | None | 4 | None |
| 07.02.2025 | Pre-defined keyword:  Police custody | None | 21 | None |

Additional pages browsed:

| **Date** | **Pages Browsed** | **Relevant studies/reports reviewed at full text and referred for second review** |
| --- | --- | --- |
| 07.02.2025 | Home > Education and Resources | None |
| 07.02.2025 | Home > Education and Resources > Resources | None |
| 07.02.2025 | Home > About the OPI > Reports and Publications | None |

**SA Health**

[www.sahealth.sa.gov.au](http://www.sahealth.sa.gov.au)

| **Search Date** | **Search Term/s** | **Limits / Filters** | **Number of Results** | **Relevant studies/reports reviewed at full text and referred for second review** |
| --- | --- | --- | --- | --- |
| 07.02.2025 | Pre-defined keyword:  Police custody | None | 17 | None |
| 07.02.2025 | Pre-defined keyword:  Police cells | None | 111 | None in the first 100 |
| 07.02.2025 | Pre-defined keyword:  Police detainees | None | 2 | None |
| 07.02.2025 | Pre-defined keyword:  Custodial care | None | 17 | None |
| 07.02.2025 | Pre-defined keyword:  Police detention | None | 7 | None |
| 07.02.2025 | Pre-defined keyword:  Policing | None | 3994 | None in the first 100 |
| 07.02.2025 | Pre-defined keyword: detainees | None | 2 | None |
| 07.02.2025 | Pre-defined keyword:  Offenders | None | 7 | None |
| 07.02.2025 | Pre-defined keyword:  Watchhouse | None | 0 | None |
| 07.02.2025 | Pre-defined keyword:  Custody suite | None | 2 | None |
| 07.02.2025 | Pre-defined keyword:  Short term custody | None | 1 | None |
| 07.02.2025 | Pre-defined keyword:  Police watchhouse | None | 0 | None |

Additional pages browsed:

| **Date** | **Pages Browsed** | **Relevant studies/reports reviewed at full text and referred for second review** |
| --- | --- | --- |
| 07.02.2025 | Home > Public Health | None |
| 07.02.2025 | Home > Services | None |
| 07.02.2025 | Home > About us > Health statistics | None |
| 07.02.2025 | Home > About us > Health statistics > Alcohol and drug statistics | None |
| 07.02.2025 | Home > About us > Health statistics > Aboriginal health outcome statistics | None |
| 07.02.2025 | Home > About us > Health statistics > Public health statistics | None |
| 07.02.2025 | Home > About us > Health and medical research | None |
| 07.02.2025 | Home > About us > Publications > Reports | None |

## Grey Literature Searches: Tasmanian Websites

**Tasmania Police**

[www.police.tas.gov.au](http://www.police.tas.gov.au)

- When using the general search function on the Tasmania Police website, no limits or filters can be used so only the first 100 results were reviewed.

| **Search Date** | **Search Term/s** | **Limits / Filters** | **Number of Results** | **Relevant studies/reports reviewed at full text and referred for second review** |
| --- | --- | --- | --- | --- |
| 07.02.2025 | Pre-defined keyword:  Health | None | 177 | None in first 100 |
| 07.02.2025 | Pre-defined keyword:  Mental health | None | 32 | None |
| 07.02.2025 | Pre-defined keyword:  Medical care | None | 27 | None |
| 07.02.2025 | Pre-defined keyword:  Treatment | None | 313 | None in the first 100 |
| 07.02.2025 | Pre-defined keyword:  Medication | None | 47 | None |
| 07.02.2025 | Pre-defined keyword:  Disease | None | 4 | None |
| 07.02.2025 | Pre-defined keyword:  Diagnosis | None | 0 | None |
| 07.02.2025 | Pre-defined keyword:  Morbidity | None | 0 | None |
| 07.02.2025 | Pre-defined keyword:  Mortality | None | 0 | None |
| 07.02.2025 | Pre-defined keyword:  Disability | None | 11 | None |
| 07.02.2025 | Pre-defined keyword:  Prevalence | None | 2 | None |
| 07.02.2025 | Pre-defined keyword:  Incidence | None | 18 | None |
| 07.02.2025 | Pre-defined keyword:  Detainees | None | 2 | None |

Additional pages browsed:

| **Date** | **Pages Browsed** | **Relevant studies/reports reviewed at full text and referred for second review** |
| --- | --- | --- |
| 07.02.2025 | Home > Information Disclosure | None |
| 07.02.2025 | Home > About us | None |

**Department of Justice**

[www.justice.tas.gov.au](http://www.justice.tas.gov.au)

| **Search Date** | **Search Term/s** | **Limits / Filters** | **Number of Results** | **Relevant studies/reports reviewed at full text and referred for second review** |
| --- | --- | --- | --- | --- |
| 07.02.2025 | Pre-defined keyword:  Health | None | 112 | None |
| 07.02.2025 | Pre-defined keyword:  Mental health | None | 117 | None |
| 07.02.2025 | Pre-defined keyword:  Medical care | None | 119 | None |
| 07.02.2025 | Pre-defined keyword:  Treatment | None | 47 | None |
| 07.02.2025 | Pre-defined keyword:  Police custody | None | 104 | None |
| 07.02.2025 | Pre-defined keyword:  Medication | None | 5 | None |
| 07.02.2025 | Pre-defined keyword:  Disease | None | 19 | None |
| 07.02.2025 | Pre-defined keyword:  Diagnosis | None | 3 | None |
| 07.02.2025 | Pre-defined keyword:  Morbidity | None | 6 | None |
| 07.02.2025 | Pre-defined keyword:  Mortality | None | 7 | None |
| 07.02.2025 | Pre-defined keyword:  Disability | None | 51 | None |
| 07.02.2025 | Pre-defined keyword:  Prevalence | None | 3 | None |
| 07.02.2025 | Pre-defined keyword:  Incidence | None | 9 | None |
| 07.02.2025 | Iterative search: health in police custody | None | 157 | None |

Additional pages browsed:

| **Date** | **Pages Browsed** | **Relevant studies/reports reviewed at full text and referred for second review** |
| --- | --- | --- |
| 07.02.2025 | Home > About us > Reports and publications | None |
| 07.02.2025 | Home > About us > Access to information | None |

**Ombudsman Tasmania**

[www.ombudsman.tas.gov.au](http://www.ombudsman.tas.gov.au)

- Searches results on the Ombudsman Tasmania website are listed only by their title, which usually consists only of a Surname and a report number. It provides no information or description of what the document contains. As a result, using search terms was not effective at identifying useful documents. Only a few searches were run before switching to a browsing strategy.

| **Search Date** | **Search Term/s** | **Limits / Filters** | **Number of Results** | **Relevant studies/reports reviewed at full text and referred for second review** |
| --- | --- | --- | --- | --- |
| 07.02.2025 | Pre-defined keyword:  Police custody | None | 90 | None |
| 07.02.2025 | Pre-defined keyword:  Police cells | None | 88 | None |
| 07.02.2025 | Pre-defined keyword:  detainees | None | 4 | None |
| 07.02.2025 | Pre-defined keyword:  Watchhouse | None | 0 | None |

Additional pages browsed:

| **Date** | **Pages Browsed** | **Relevant studies/reports reviewed at full text and referred for second review** |
| --- | --- | --- |
| 07.02.2025 | Home > Publications  Reviewed: annual reports, case summaries, fact sheets, investigation reports, public interest disclosure materials, and media releases | None |

**Tasmanian Department of Health**

[www.health.tas.gov.au](http://www.health.tas.gov.au)

| **Search Date** | **Search Term/s** | **Limits / Filters** | **Number of Results** | **Relevant studies/reports reviewed at full text and referred for second review** |
| --- | --- | --- | --- | --- |
| 07.02.2025 | Pre-defined keyword:  Police custody | None | 2 | None |
| 07.02.2025 | Pre-defined keyword:  Police cells | None | 1 | None |
| 07.02.2025 | Pre-defined keyword:  Police detainees | None | 0 | None |
| 07.02.2025 | Pre-defined keyword:  Custodial care | None | 1 | None |
| 07.02.2025 | Pre-defined keyword:  Police detention | None | 4 | None |
| 07.02.2025 | Pre-defined keyword:  Policing | None | 3 | None |
| 07.02.2025 | Pre-defined keyword:  Offenders | None | 2 | None |
| 07.02.2025 | Pre-defined keyword:  Watchhouse | None | 0 | None |
| 07.02.2025 | Pre-defined keyword:  Custody suite | None | 1 | None |
| 07.02.2025 | Pre-defined keyword:  Short term custody | None | 1 | None |
| 07.02.2025 | Pre-defined keyword:  Police watchhouse | None | 0 | None |

Additional pages browsed:

| **Date** | **Pages Browsed** | **Relevant studies/reports reviewed at full text and referred for second review** |
| --- | --- | --- |
| 07.02.2025 | Home | None |
| 07.02.2025 | Home > Health topics | None |
| 07.02.2025 | Home > Hospital and health services | None |
| 07.02.2025 | Home > for patients | None |
| 07.02.2025 | Home > for professionals | None |

## Grey Literature Searches: Victorian Websites

**Victoria Police**

Website: [www.police.vic.gov.au](http://www.police.vic.gov.au)

| **Search Date** | **Search Term/s** | **Limits / Filters** | **Number of Results** | **Relevant studies/reports reviewed at full text and referred for second review** |
| --- | --- | --- | --- | --- |
| 07.02.2025 | Pre-defined keyword:  Health | None | 78 | None |
| 07.02.2025 | Pre-defined keyword:  Mental health | None | 88 | None |
| 07.02.2025 | Pre-defined keyword:  Medical care | None | 98 | None |
| 07.02.2025 | Pre-defined keyword:  Treatment | None | 24 | None |
| 07.02.2025 | Pre-defined keyword:  Medication | None | 6 | None |
| 07.02.2025 | Pre-defined keyword:  Disease | None | 6 | None |
| 07.02.2025 | Pre-defined keyword:  Diagnosis | None | 0 | None |
| 07.02.2025 | Pre-defined keyword:  Morbidity | None | 0 | None |
| 07.02.2025 | Pre-defined keyword:  Mortality | None | 0 | None |
| 07.02.2025 | Pre-defined keyword:  Disability | None | 42 | None |
| 07.02.2025 | Pre-defined keyword:  Prevalence | None | 4 | None |
| 07.02.2025 | Pre-defined keyword:  Incidence | None | 7 | None |
| 07.02.2025 | Pre-defined keyword:  detainees | None | 0 | None |

Additional pages browsed:

| **Date** | **Pages Browsed** | **Relevant studies/reports reviewed at full text and referred for second review** |
| --- | --- | --- |
| 07.02.2025 | Home > About Victoria Police | None |
| 07.02.2025 | Home > Statistics | None |
| 07.02.2025 | Home > Statistics > Crime Statistics | None, but additional website to search identified: <https://www.crimestatistics.vic.gov.au/> - added below. |

**Crime Statistics Agency**

Website: <https://www.crimestatistics.vic.gov.au/>

| **Search Date** | **Search Term/s** | **Limits / Filters** | **Number of Results** | **Relevant studies/reports reviewed at full text and referred for second review** |
| --- | --- | --- | --- | --- |
| 07.02.2025 | Pre-defined keyword:  Health | None | 75 | None |
| 07.02.2025 | Pre-defined keyword:  Mental health | None | 75 | None |
| 07.02.2025 | Pre-defined keyword:  Medical care | None | 6 | None |
| 07.02.2025 | Pre-defined keyword:  Treatment | None | 2 | None |
| 07.02.2025 | Pre-defined keyword:  Medication | None | 0 | None |
| 07.02.2025 | Pre-defined keyword:  Disease | None | 0 | None |
| 07.02.2025 | Pre-defined keyword:  Diagnosis | None | 0 | None |
| 07.02.2025 | Pre-defined keyword:  Morbidity | None | 0 | None |
| 07.02.2025 | Pre-defined keyword:  Mortality | None | 0 | None |
| 07.02.2025 | Pre-defined keyword:  Disability | None | 0 | None |
| 07.02.2025 | Pre-defined keyword:  Prevalence | None | 9 | None |
| 07.02.2025 | Pre-defined keyword:  Incidence | None | 1 | None |
| 07.02.2025 | Iterative search: health in police custody | None | 258 | None in the first 100 |

Additional pages browsed:

| **Date** | **Pages Browsed** | **Relevant studies/reports reviewed at full text and referred for second review** |
| --- | --- | --- |
| 07.02.2025 | Home > Crime statistics > Latest Victorian crime data > Alleged Offender Incidents | None |
| 07.02.2025 | Home > Research and evaluation | None |
| 07.02.2025 | Home > Research and evaluation > Publications | None |
| 07.02.2025 | Home > Research and evaluation > Publications > COVID-19 | None |
| 07.02.2025 | Home > Research and evaluation > Publications > Criminal Justice Pathways | None |
| 07.02.2025 | Home > Research and evaluation > Publications > Family Violence | None |
| 07.02.2025 | Home > Research and evaluation > Publications > Drug and alcohol use and crime | None |
| 07.02.2025 | Home > Research and evaluation > Publications > female offenders | None |
| 07.02.2025 | Home > Research and evaluation > Publications > Men's violence against women | None |
| 07.02.2025 | Home > Research and evaluation > Publications > Reoffending | None |
| 07.02.2025 | Home > Research and evaluation > Publications > Sexual offences | None |
| 07.02.2025 | Home > Research and evaluation > Publications > Victims | None |
| 07.02.2025 | Home > Research and evaluation > Publications > Stalking | None |
| 07.02.2025 | Home > Research and evaluation > Publications > Youth crime | None |

**Independent Broad-based Anti-Corruption Commission**

Website: [www.ibac.vic.gov.au](http://www.ibac.vic.gov.au)

| **Search Date** | **Search Term/s** | **Limits / Filters** | **Number of Results** | **Relevant studies/reports reviewed at full text and referred for second review** |
| --- | --- | --- | --- | --- |
| 07.02.2025 | Pre-defined keyword:  Health | None | 9 | None |
| 07.02.2025 | Pre-defined keyword:  Mental health | None | 9 | None |
| 07.02.2025 | Pre-defined keyword:  Medical care | None | 0 | None |
| 07.02.2025 | Pre-defined keyword:  Treatment | None | 0 | None |
| 07.02.2025 | Pre-defined keyword:  Medication | None | 0 | None |
| 07.02.2025 | Pre-defined keyword:  Disease | None | 0 | None |
| 07.02.2025 | Pre-defined keyword:  Diagnosis | None | 0 | None |
| 07.02.2025 | Pre-defined keyword:  Morbidity | None | 0 | None |
| 07.02.2025 | Pre-defined keyword:  Mortality | None | 0 | None |
| 07.02.2025 | Pre-defined keyword:  Disability | None | 0 | None |
| 07.02.2025 | Pre-defined keyword:  Prevalence | None | 0 | None |
| 07.02.2025 | Pre-defined keyword:  Incidence | None | 12 | None |
| 07.02.2025 | Pre-defined keyword:  Detainees | None | 0 | None |
| 07.02.2025 | Iterative Search:  Custody | None | 0 | None |
| 07.02.2025 | Pre-defined keyword:  Police cells | None | 141 | 1 report identified:  14. Operation Ross: An investigation into police conduct in the Ballarat Police Service Area |

Additional pages browsed:

| **Date** | **Pages Browsed** | **Relevant studies/reports reviewed at full text and referred for second review** |
| --- | --- | --- |
| 07.02.2025 | Resource Centre > Police | None |
| 07.02.2025 | Resources: filtered by Victoria Police and types of content –investigation summaries, special reports | None |

**Victorian Ombudsman**

Website: [www.ombudsman.vic.gov.au](http://www.ombudsman.vic.gov.au)

| **Search Date** | **Search Term/s** | **Limits / Filters** | **Number of Results** | **Relevant studies/reports reviewed at full text and referred for second review** |
| --- | --- | --- | --- | --- |
| 10.02.2025 | Pre-defined keyword:  Police custody | None | 6 | None |
| 10.02.2025 | Pre-defined keyword:  Police cells | None | 8 | None |
| 10.02.2025 | Pre-defined keyword:  Police detainees | None | 0 | None |
| 10.02.2025 | Pre-defined keyword:  Custodial care | None | 4 | None |
| 10.02.2025 | Pre-defined keyword:  Police detention | None | 12 | None |
| 10.02.2025 | Pre-defined keyword:  Policing | None | 44 | None |
| 10.02.2025 | Pre-defined keyword:  Offenders | None | 14 | None |
| 10.02.2025 | Pre-defined keyword:  Watchhouse | None | 0 | None |
| 10.02.2025 | Pre-defined keyword:  Custody suite | None | 1 | None |
| 10.02.2025 | Pre-defined keyword:  Short term custody | None | 0 | None |
| 10.02.2025 | Pre-defined keyword:  Police watchhouse | None | 0 | None |

Additional pages browsed:

| **Date** | **Pages Browsed** | **Relevant studies/reports reviewed at full text and referred for second review** |
| --- | --- | --- |
| 10.02.2025 | Home > Our impact > Investigation reports | Nil new reports identified. All relevant reports already identified in database searches. |
| 10.02.2025 | Home > Our impact > Investigation reports > Investigation reports before 2014 | Nil new reports identified. All relevant reports already identified in database searches. |

**Victorian Equal Opportunity and Human Rights Commission**

Website: [www.humanrights.vic.gov.au](http://www.humanrights.vic.gov.au)

| **Search Date** | **Search Term/s** | **Limits / Filters** | **Number of Results** | **Relevant studies/reports reviewed at full text and referred for second review** |
| --- | --- | --- | --- | --- |
| 10.02.2025 | Pre-defined keyword:  Police custody | None | 7 | None |
| 10.02.2025 | Pre-defined keyword:  Police cells | None | 8 | None |
| 10.02.2025 | Pre-defined keyword:  Police detainees | None | 6 | None |
| 10.02.2025 | Pre-defined keyword:  Custodial care | None | 2 | None |
| 10.02.2025 | Pre-defined keyword:  Police detention | None | 17 | None |
| 10.02.2025 | Pre-defined keyword:  Policing | None | 89 | None |
| 10.02.2025 | Pre-defined keyword:  Offenders | None | 20 | None |
| 10.02.2025 | Pre-defined keyword:  Watchhouse | None | 0 | None |
| 10.02.2025 | Pre-defined keyword:  Custody suite | None | 0 | None |
| 10.02.2025 | Pre-defined keyword:  Short term custody | None | 0 | None |
| 10.02.2025 | Pre-defined keyword:  Police watchhouse | None | 0 | None |

Additional pages browsed:

| **Date** | **Pages Browsed** | **Relevant studies/reports reviewed at full text and referred for second review** |
| --- | --- | --- |
| 10.02.2025 | About us> Resources > Reviewed all 114 resources listed, nil filters | None |

**Department of Health Victoria**

Website: [www.health.vic.gov.au](http://www.health.vic.gov.au)

| **Search Date** | **Search Term/s** | **Limits / Filters** | **Number of Results** | **Relevant studies/reports reviewed at full text and referred for second review** |
| --- | --- | --- | --- | --- |
| 10.02.2025 | Pre-defined keyword:  Police custody | None | 104 | None |
| 10.02.2025 | Pre-defined keyword:  Police cells | None | 125 | None |
| 10.02.2025 | Pre-defined keyword:  Police detainees | None | 95 | None |
| 10.02.2025 | Pre-defined keyword:  Custodial care | None | 1682 | None in the first 100 |
| 10.02.2025 | Pre-defined keyword:  Police detention | None | 121 | None |
| 10.02.2025 | Pre-defined keyword:  Policing | None | 0 | None |
| 10.02.2025 | Pre-defined keyword:  Offenders | None | 9 | None |
| 10.02.2025 | Pre-defined keyword:  Watchhouse | None | 0 | None |
| 10.02.2025 | Pre-defined keyword:  Custody suite | None | 91 | None |
| 10.02.2025 | Pre-defined keyword:  Police watchhouse | None | 95 | None |

Additional pages browsed:

| **Date** | **Pages Browsed** | **Relevant studies/reports reviewed at full text and referred for second review** |
| --- | --- | --- |
| 10.02.2025 | Home > Alcohol & other drugs | None |
| 10.02.2025 | Home > Alcohol & other drugs > Alcohol and other drug treatment services > Forensic services | None |
| 10.02.2025 | Home > Public Health | None |
| 10.02.2025 | Home > Mental Health | None |

**Department of Justice and Community Safety Victoria**

Website: [www.justice.vic.gov.au](http://www.justice.vic.gov.au)

| **Search Date** | **Search Term/s** | **Limits / Filters** | **Number of Results** | **Relevant studies/reports reviewed at full text and referred for second review** |
| --- | --- | --- | --- | --- |
| 10.02.2025 | Pre-defined keyword:  Health | None | 187 | None in the first 100 |
| 10.02.2025 | Pre-defined keyword:  Mental health | None | 89 | None |
| 10.02.2025 | Pre-defined keyword:  Medical care | None | 44 | None |
| 10.02.2025 | Pre-defined keyword:  Treatment | None | 61 | None |
| 10.02.2025 | Pre-defined keyword:  Medication | None | 3 | None |
| 10.02.2025 | Pre-defined keyword:  Disease | None | 22 | None |
| 10.02.2025 | Pre-defined keyword:  Diagnosis | None | 2 | None |
| 10.02.2025 | Pre-defined keyword:  Morbidity | None | 1 | None |
| 10.02.2025 | Pre-defined keyword:  Mortality | None | 1 | None |
| 10.02.2025 | Pre-defined keyword:  Disability | None | 49 | None |
| 10.02.2025 | Pre-defined keyword:  Prevalence | None | 1 | None |
| 10.02.2025 | Pre-defined keyword:  Incidence | None | 2 | None |
| 10.02.2025 | Pre-defined keyword:  Police custody | None | 56 | None |

Additional pages browsed:

| **Date** | **Pages Browsed** | **Relevant studies/reports reviewed at full text and referred for second review** |
| --- | --- | --- |
| 10.02.2025 | Home > Resources > Publications and reports | Reviewed all 9 pages of publications. Nil sent for second review. |

## Grey Literature Searches: Western Australian Websites

**WA Police Force**

Website: [www.police.wa.gov.au](http://www.police.wa.gov.au)

- General searches without filters on the WA Police website searches all WA government sites.

| **Search Date** | **Search Term/s** | **Limits / Filters** | **Number of Results** | **Relevant studies/reports reviewed at full text and referred for second review** |
| --- | --- | --- | --- | --- |
| 10.02.2025 | Pre-defined keyword:  Health | Results from Western Australia Police Force only | 29 | None |
| 10.02.2025 | Pre-defined keyword:  Mental health | Results from Western Australia Police Force only | 32 | None |
| 10.02.2025 | Pre-defined keyword:  Medical care | Results from Western Australia Police Force only | 36 | None |
| 10.02.2025 | Pre-defined keyword:  Treatment | Results from Western Australia Police Force only | 31 | None |
| 10.02.2025 | Pre-defined keyword:  Medication | Results from Western Australia Police Force only | 1 | None |
| 10.02.2025 | Pre-defined keyword:  Disease | Results from Western Australia Police Force only | 0 | None |
| 10.02.2025 | Pre-defined keyword:  Diagnosis | Results from Western Australia Police Force only | 0 | None |
| 10.02.2025 | Pre-defined keyword:  Morbidity | Results from Western Australia Police Force only | 0 | None |
| 10.02.2025 | Pre-defined keyword:  Mortality | Results from Western Australia Police Force only | 0 | None |
| 10.02.2025 | Pre-defined keyword:  Disability | Results from Western Australia Police Force only | 5 | None |
| 10.02.2025 | Pre-defined keyword:  Prevalence | Results from Western Australia Police Force only | 1 | None |
| 10.02.2025 | Pre-defined keyword:  Incidence | Results from Western Australia Police Force only | 4 | None |

Additional pages browsed:

| **Date** | **Pages Browsed** | **Relevant studies/reports reviewed at full text and referred for second review** |
| --- | --- | --- |
| 10.02.2025 | Home > Western Australia Police Force > About WA Police Force | None |
| 10.02.2025 | Home > Western Australia Police Force > Juvenile justice | None |

**Department of Justice**

Website: [www.wa.gov.au/organisation/department-of-justice](http://www.wa.gov.au/organisation/department-of-justice)

- General searches without filters on the WA Department of Justice website searches all WA government sites.

| **Search Date** | **Search Term/s** | **Limits / Filters** | **Number of Results** | **Relevant studies/reports reviewed at full text and referred for second review** |
| --- | --- | --- | --- | --- |
| 10.02.2025 | Pre-defined keyword:  Police Custody | Results from Department of Justice only | 130 | When excluding media or announcements, there were only 41 results remaining. None referred for 2^nd^ review. |
| 10.02.2025 | Pre-defined keyword:  Health | Results from Department of Justice only | 120 | When excluding media or announcements, there were only 31 results remaining. None referred for 2^nd^ review. |
| 10.02.2025 | Pre-defined keyword:  Mental health | Results from Department of Justice only | 126 | When excluding media or announcements, there were only 36 results remaining. None referred for 2^nd^ review. |
| 10.02.2025 | Pre-defined keyword:  Medical care | Results from Department of Justice only | 156 | When excluding media or announcements, there were only 41 results remaining. None referred for 2^nd^ review. |
| 10.02.2025 | Pre-defined keyword:  Treatment | Results from Department of Justice only | 41 | When excluding media or announcements, there were only 11 results remaining. None referred for 2^nd^ review. |
| 10.02.2025 | Pre-defined keyword:  Medication | Results from Department of Justice only | 5 | None |
| 10.02.2025 | Pre-defined keyword:  Disease | Results from Department of Justice only | 8 | None |
| 10.02.2025 | Pre-defined keyword:  Diagnosis | Results from Department of Justice only | 0 | None |
| 10.02.2025 | Pre-defined keyword:  Morbidity | Results from Department of Justice only | 0 | None |
| 10.02.2025 | Pre-defined keyword:  Mortality | Results from Department of Justice only | 1 | None |
| 10.02.2025 | Pre-defined keyword:  Disability | Results from Department of Justice only | 18 | None |
| 10.02.2025 | Pre-defined keyword:  Prevalence | Results from Department of Justice only | 3 | None |
| 10.02.2025 | Pre-defined keyword:  Incidence | Results from Department of Justice only | 0 | None |

Additional pages browsed:

| **Date** | **Pages Browsed** | **Relevant studies/reports reviewed at full text and referred for second review** |
| --- | --- | --- |
| 10.02.2025 | Home > Department of Justice > Statistics | None |
| 10.02.2025 | Home > Department of Justice > Publications | None |

**Ombudsman Western Australia**

Website: [www.ombudsman.wa.gov.au](http://www.ombudsman.wa.gov.au)

- There is no search function on the Ombudsman WA website. Only browsing could be completed.

Additional pages browsed:

| **Date** | **Pages Browsed** | **Relevant studies/reports reviewed at full text and referred for second review** |
| --- | --- | --- |
| 10.02.2025 | Home > Publications > Ombudsman reports | Reviewed all publications on webpage from 2000 onwards, none referred for second review. |

**WA Health**

Website: [www.health.wa.gov.au](http://www.health.wa.gov.au)

- When searching the WA Department of Health website, no more than 50 results are displayed per search.

| **Search Date** | **Search Term/s** | **Limits / Filters** | **Number of Results** | **Relevant studies/reports reviewed at full text and referred for second review** |
| --- | --- | --- | --- | --- |
| 10.02.2025 | Pre-defined keyword:  Police custody | None | 50 | None |
| 10.02.2025 | Pre-defined keyword:  Police cells | None | 50 | None |
| 10.02.2025 | Pre-defined keyword:  Police detainees | None | 50 | None |
| 10.02.2025 | Pre-defined keyword:  Custodial care | None | 50 | None |
| 10.02.2025 | Pre-defined keyword:  Police detention | None | 50 | None |
| 10.02.2025 | Pre-defined keyword:  Policing | None | 0 | None |
| 10.02.2025 | Pre-defined keyword:  Offenders | None | 9 | None |
| 10.02.2025 | Pre-defined keyword:  Watchhouse | None | 0 | None |
| 10.02.2025 | Pre-defined keyword:  Custody suite | None | 37 | None |
| 10.02.2025 | Pre-defined keyword:  Short term custody | None | 50 | None |
| 10.02.2025 | Pre-defined keyword:  Police watchhouse | None | 50 | None |

Additional pages browsed:

| **Date** | **Pages Browsed** | **Relevant studies/reports reviewed at full text and referred for second review** |
| --- | --- | --- |
| 10.02.2025 | Home > Reports & publications | None |
| 10.02.2025 | Home > Our performance | None |

## Articles Referred for Second Review

| **Article** | **Source** | **Second reviewer decision** | **Reason if excluded** |
| --- | --- | --- | --- |
| 1. Post Visit Summary: Christmas Island Police Station | Commonwealth Ombudsman Website | Include | N/A |
| 2. Post Visit Summary: Cocos Island Police Station | Commonwealth Ombudsman Website | Include | N/A |
| 3. Post Visit Summaries: ACT Policing Watch House and ACT Police Stations | Commonwealth Ombudsman Website | Include | N/A |
| 4. Post Visit Summary: Jervis Bay | Commonwealth Ombudsman Website | Include | N/A |
| 5. Drug use monitoring in Australia: Drug use among police detainees, 2019 | Australian Institute of Criminology Website | Include | N/A |
| 6. Drug use monitoring in Australia: Drug use among police detainees, 2020 | Australian Institute of Criminology Website | Include | N/A |
| 7. Drug use monitoring in Australia: Drug use among police detainees, 2021 | Australian Institute of Criminology Website | Include | N/A |
| 8. Alcohol and disorderly conduct on Friday and Saturday nights: Findings from the DUMA program | Australian Institute of Criminology Website | Include | N/A |
| 9. Victimisation and fear of crime among a sample of police detainees | Australian Institute of Criminology Website | Include | N/A |
| 10. Operation Kimbla - Final Report | Law Enforcement Conduct Commission Website | Include | N/A |
| 11. Extraordinary Restraint: Spit Hood & Emergency Restraint Chair Use on Children in Police Custody | Ombudsman Northern Territory Website | Include | N/A |
| 12. Independent review into investigations of police-related deaths, and domestic and family violence deaths in Queensland. | Department of Justice and Attorney-General Website (Queensland) | Include | N/A |
| 13. Cairns and Murgon watch houses inspection report: Focus on detention of children | Queensland Ombudsman Website | Include | N/A |
| 14. Operation Ross: An investigation into police conduct in the Ballarat Police Service Area | Independent Broad-based Anti-Corruption Commission Website | Include | N/A |
