# Supplementary material for: The determinants of health and health status of individuals in police custody in Australia: A scoping review
Source: PLoS One. 2025 Dec 30;20(12):e0338957. doi: 10.1371/journal.pone.0338957 (PMC12753082; doi:10.1371/journal.pone.0338957)
Supplement: S3 Appendix — (DOCX) [file pone.0338957.s003.docx]

# **S3 Appendix: Reference Search of Relevant Review Articles**

When review articles, or articles that contained summaries of previously published data of interest were identified during the screening process they were added to this list. Later, after all database and grey literature website screening had been completed, we reviewed all the references listed in the below articles to try and find additional articles that may have been missed in our database and website searches. Any new articles identified that appeared to meet inclusion criteria at the full text level, were referred to a second reviewer for consideration of inclusion.

| **Study** | **Title** | **Additional Studies referred for 2^nd^ review** |
| --- | --- | --- |
| Wardrop et al., 2021 [1] | Structures, processes and outcomes of health care for people detained in short-term police custody settings: A scoping review | None. |
| Crilly et al., 2019 [2] | Clinical characteristics and outcomes of patient presentations to the emergency department via police: A scoping review | None. |
| Sabrinskas et al., 2022 [3] | Suicide by hanging: A scoping review | None. |
| McKinnon et al., 2016 [4] | Police custody health care: a review of health morbidity, models of care and innovations within police custody in the UK, with international comparisons | None. |
| McCausland et al., 2017 [5] | Indigenous People, Mental Health, Cognitive Disability and the Criminal Justice System | None. |
| Freckelton & List 2009 [6] | Asperger's Disorder, Criminal Responsibility and Criminal Culpability | None. |
| Anthony 2021 [7] | Biopower of Colonialism in Carceral Contexts: Implications for Aboriginal Deaths in Custody | None. |
| National Indigenous Drug and Alcohol Committee 2009 [8] | Bridges and barriers: addressing Indigenous incarceration and health | None. |
| Sweeney & Payne 2012 [9] | Drug use among police detainees: a comparative analysis of DUMA and the US Arrestee Drug Abuse Monitoring program | None. |
| Makkai 2001 [10] | Drug Use Amongst Police Detainees: Some Comparative Data | None. |
| Milner 2004 [11] | Drug Use Monitoring in Australia | No references to review. |
| Everuss 2023 [12] | Everyday sovereign exclusion: conceptualising police violence and deaths in custody as a racial production of homo sacer | None. |
| Crilly et al., 2022 [13] | Health care in police watch-houses: a challenge and an opportunity | None. |
| Behrendt 2013 [14] | Indigenous people and the criminal justice system | None. |
| Perera 2023 [15] | “Never Settler Enough”: The Double Economy of Terror and Deaths in Custody in Australia | None. |
| Walker et al., 2023 [16] | Police custody in Australia: A call for transparency and accountability | Two relevant studies identified and referred for 2^nd^ review:   1. Queensland Crime and Corruption Commission 2017: Police use of force in Queensland watch-houses 2. Community Development and Justice Standing Committee 2013. In Safe Custody: Inquiry into Custodial Arrangements in Police Lock-Ups |
| Ogloff 2013 [17] | Policing services with mentally ill people: developing greater understanding and best practice | None. |
| McKinnon et al., 2023 [18] | Screening for mental disorders in police custody settings | None. |
| Blue 2017 [19] | Seeing Ms. Dhu: inquest, conquest, and (in)visibility in black women’s deaths in custody | None. |
| Grant 2008 [20] | The case for single cells and alternative ways of viewing custodial accommodation for Australian Aboriginal peoples | None. |
| Ogloff 2007 [21] | The identification of mental disorders in the criminal justice system | None. |
| Lin et al., 2020 [22] | Youth justice in Australia: Themes from recent inquiries | One relevant study identified and referred for 2^nd^ review:   1. Royal Commission into the Protection & Detention of Children in the Northern Territory, Volume 2B |
| Cunneen 2006 [23] | Aboriginal Deaths in Custody: A Continuing Systematic Abuse | None. |
| Australian Institute of Criminology 2008 [24] | Deaths in custody: overview of trends, 1980-2006 | None. |
| Australian Institute of Criminology 2006 [25] | Deaths in prison custody: sentenced and remand prisoners | None. |
| Mouzos & Smith 2006 [26] | Drug use among police detainees, 2005 | None. |
| Bradford & Payne 2012 [27] | Illicit Drug Use and Property Offending among Police Detainees | None. |
| Australian Institute of Criminology 2003 [28] | Indigenous deaths in custody in Australia | None. |
| Fitzgerald & Chilvers 2002 [29] | Multiple drug use among police detainees | None. |

## **Articles assessed by second reviewer**

| **Article** | **Source** | **Second reviewer decision** | **Reason if excluded** |
| --- | --- | --- | --- |
| Queensland Crime and Corruption Commission 2017: Police use of force in Queensland watch-houses [30] | References of Walker et al., 2023 | Include | N/A |
| Community Development and Justice Standing Committee 2013. In Safe Custody: Inquiry into Custodial Arrangements in Police Lock-Ups [31] | References of Walker et al., 2023 | Include | N/A |
| Report of the Royal Commission and Board Inquiry into the Protection & Detention of Children in the Northern Territory, Volume 2B [32] | References of Lin et al., 2020 | Include | N/A |

**References**

1. Wardrop R, Ranse J, Chaboyer W, Crilly J. Structures, processes and outcomes of health care for people detained in short-term police custody settings: A scoping review. J Forensic Leg Med. 2021;81:102198. doi: <https://doi.org/10.1016/j.jflm.2021.102198>.

2. Crilly J, Johnston AN, Wallis M, Polong-Brown J, Heffernan E, Fitzgerald G, et al. Review article: Clinical characteristics and outcomes of patient presentations to the emergency department via police: A scoping review. Emerg Med Australas. 2019;31(4):506-15. Epub 20190521. doi: 10.1111/1742-6723.13300. PubMed PMID: 31115191.

3. Sabrinskas R, Hamilton B, Daniel C, Oliffe J. Suicide by hanging: A scoping review. Int J Ment Health Nurs. 2022;31(2):278-94. Epub 20211125. doi: 10.1111/inm.12956. PubMed PMID: 34825469.

4. McKinnon IG, Thomas SD, Noga HL, Senior J. Police custody health care: a review of health morbidity, models of care and innovations within police custody in the UK, with international comparisons. Risk Manag Health Policy. 2016;9:213-26. Epub 20160915. doi: 10.2147/rmhp.S61536. PubMed PMID: 27695373; PubMed Central PMCID: PMCPMC5028165.

5. McCausland R, McEntyre E, Baldry E. Indigenous people, mental health, cognitive disability and the criminal justice system. Indigenous Justice Clearinghouse. 2017;22.

6. Freckelton I, List D. Asperger's Disorder, Criminal Responsibility and Criminal Culpability. Psychiatry, Psychology & Law. 2009;16(1):16-40. doi: 10.1080/13218710902887483.

7. Anthony T, Blagg H. Biopower of Colonialism in Carceral Contexts: Implications for Aboriginal Deaths in Custody. J Bioeth Inq. 2021;18(1):71-82. doi: <https://dx.doi.org/10.1007/s11673-020-10076-x>.

8. National Indigenous Alcohol and Drug Committee. Bridges and barriers: addressing Indigenous incarceration and health. 2009.

9. Sweeney J, Payne J. Drug use among police detainees: a comparative analysis of DUMA and the US Arrestee Drug Abuse Monitoring program. Research in Practice. 2012;27.

10. Makkai T. Drug Use Amongst Police Detainees: Some Comparative Data. Trends & Issues in Crime and Criminal Justice. 2001;191:1-6.

11. Milner L. Drug Use Monitoring in Australia. Of Substance. 2004;2(4):23-4.

12. Everuss L. Everyday sovereign exclusion: conceptualising police violence and deaths in custody as a racial production of homo sacer. Distinktion: Journal of Social Theory. 2023;24(3):383-404. doi: 10.1080/1600910X.2023.2220933.

13. Crilly JL, Brandenburg C, Kinner SA, Heffernan E, Byrnes J, Lincoln C, et al. Health care in police watch-houses: a challenge and an opportunity. Med J Aust. 2022. doi: <https://dx.doi.org/10.5694/mja2.51688>.

14. Behrendt L. Indigenous people and the criminal justice system. Hot Topics: Legal Issues in Plain Language. 2013;(86):7-10.

15. Perera S, Pugliese J. “Never Settler Enough”: The Double Economy of Terror and Deaths in Custody in Australia. Filoz Vestn. 2023;44(2):307-28. doi: 10.3986/fv.44.2.14.

16. Walker S, Wilson M, Seear K, Doyle M, Saich F, Stoove M, et al. Police custody in Australia: A call for transparency and accountability. Australian and New Zealand journal of public health. 2023;47(2):100040. doi: <https://dx.doi.org/10.1016/j.anzjph.2023.100040>.

17. Ogloff JRP. Policing services with mentally ill people : developing greater understanding and best practice. Australian psychologist. 2013;48(1):57-68. PubMed PMID: cinch.293078.

18. McKinnon I, Moore J, Lyall A, Forrester A. Screening for mental disorders in police custody settings. BJPsych Advances. 2023;29(6):407-16. doi: <https://dx.doi.org/10.1192/bja.2022.25>. PubMed PMID: 2028578122.

19. Blue E. Seeing Ms. Dhu: inquest, conquest, and (in)visibility in black women’s deaths in custody. Settler Colon Stud. 2017;7(3):299-320. doi: 10.1080/2201473X.2016.1229294.

20. Grant E. The case for single cells and alternative ways of viewing custodial accommodation for Australian Aboriginal peoples. Flinders journal of law reform. 2008;10(3):631-47. PubMed PMID: cinch.280138.

21. Ogloff JRP, Davis MR, Rivers G, Ross S. The identification of mental disorders in the criminal justice system. Trends & Issues in Crime & Criminal Justice. 2007;(334):1-6.

22. Lin B, Clancey G, Wang S. Youth justice in Australia: Themes from recent inquiries. Trends and Issues in Crime and Criminal Justice [electronic resource]. 2020;(605):1-19. PubMed PMID: agispt.20201215041190.

23. Cunneen C. Aboriginal Deaths in Custody: A Continuing Systematic Abuse. Social Justice. 2006;33(4):37-51. PubMed PMID: 231923235.

24. Australian Institute of Criminology. Deaths in custody: overview of trends, 1980-2006. Crime Facts Info. 2008;166.

25. Australian Institute of Criminology. Deaths in prison custody: sentenced and remand prisoners. Crime Facts Info. 2006;114.

26. Mouzos J, Smith L. Drug use among police detainees, 2005. Trends & Issues in Crime & Criminal Justice. 2006;(319):1-6.

27. Bradford D, Payne J. Illicit Drug Use and Property Offending among Police Detainees. Contemporary Issues in Crime and Justice. 2012;(157):1-12.

28. Australian Institute of Criminology. Indigenous deaths in custody in Australia. Crime Facts Info. 2003.

29. Fitzgerald J, Chilvers M. Multiple drug use among police detainees. Contemporary Issues in Crime and Justice. 2002;65.

30. The State of Queensland (Crime and Corruption Commission). Police use of force in Queensland watch-houses. Brisbane: Crime and Corruption Commission, 2017.

31. Community Development and Justice Standing Committee (Parliament of Western Australia). In Safe Custody: Inquiry into Custodial Arrangements in Police Lock-ups. Perth: Legislative Assembly Parliament of Western Australia, 2013.

32. Royal Commission into the Protection and Detention of Children in the Northern Territory, Volume 2B. Canberra: Commonwealth of Australia, 2017.
