# Supplementary material for: The determinants of health and health status of individuals in police custody in Australia: A scoping review
Source: PLoS One. 2025 Dec 30;20(12):e0338957. doi: 10.1371/journal.pone.0338957 (PMC12753082; doi:10.1371/journal.pone.0338957)
Supplement: S4 Appendix — (DOCX) [file pone.0338957.s004.docx]

# **S4 Appendix: Excluded Studies**

The following tables contains lists of studies that were excluded during full text screening. These studies might appear to meet the inclusion criteria when reviewing only the title or abstract. The reason for exclusion is also recorded (in some cases both a primary and secondary reason for exclusion is listed).

**Table 1: Studies from database searches excluded at full text stage**

| **Study** | **Title** | **Primary Reason for Exclusion** |
| --- | --- | --- |
| Crilly 2019 [1] | A structure and process evaluation of a police Watch House Emergency Nurse (WHEN) model of care. | Concept: doesn’t report on health, or determinants of health |
| Lilburn 2001 [2] | Arresting moments: identifying risks for women and their children from the time of police arrest | Concept: doesn’t report on health, or determinants of health |
| Freckelton 2019 [3] | Attention Deficit Hyperactivity Disorder (ADHD) and the criminal law | Concept: doesn’t report on health, or determinants of health |
| Gray 2000 [4] | Beating the grog: an evaluation of the Tennant Creek liquor licensing restrictions | Concept: doesn’t report on health, or determinants of health |
| Cockram 2005 [5] | Careers of offenders with an intellectual disability: the probabilities of rearrest | Concept: doesn’t report on health, or determinants of health |
| Hollander 2012 [6] | Challenges relating to the interface between crisis mental health clinicians and police when engaging with people with a mental illness | Concept: doesn’t report on health, or determinants of health |
| Toepfer 2008 [7] | City Watch House Community Nursing Service: the Clinical Practice Consultants’ response to homelessness in police holding cells | Concept: doesn’t report on health, or determinants of health |
| Baldry & Dowse 2013 [8] | Compounding mental and cognitive disability and disadvantage: Police as care managers | Concept: doesn’t report on health, or determinants of health |
| Johnson et al., 2019 [9] | Creating communication accessibility for people with communication disabilities at police stations | Concept: doesn’t report on health, or determinants of health |
| McCarthy et al., 2021 [10] | Defendants with intellectual disability and autism spectrum conditions: The perspective of clinicians working across three jurisdictions | Concept: doesn’t report on health, or determinants of health |
| Crilly et al., 2020 [11] | Effect of a 24/7 nursing presence in a police watch house on police presentations to the emergency department. | Concept: doesn’t report on health, or determinants of health |
| Victorian Law Reform Commission 2005 [12] | Family violence police holding powers: interim report | Concept: doesn’t report on health, or determinants of health |
| Goldsmid 2015 [13] | Findings from the DUMA program: Internet access, and frequency and nature of use among police detainees | Concept: doesn’t report on health, or determinants of health |
| Tasmania Law Reform Institute 2006 [14] | Intoxication and criminal responsibility | Concept: doesn’t report on health, or determinants of health |
| Bartels 2011[15] | Knife crime: Recent data on carriage and use | Concept: doesn’t report on health, or determinants of health |
| Payne & Wong 2018 [16] | Measuring drug dependence in police custody: An item response theory and differential item function analysis of UNCOPE in Australia | Concept: doesn’t report on health, or determinants of health |
| Fogarty 2016 [17] | Mental health: Police detention of people who appear mentally ill or mentally disturbed | Concept: doesn’t report on health, or determinants of health |
| Baldry et al., 2012 [18] | People with intellectual and other cognitive disability in the criminal justice system: report for NSW Family and Community Services, Ageing, Disability and Home Care | Concept: doesn’t report on health, or determinants of health |
| Brandenburg et al., 2024 [19] | Police perspectives on the economic considerations of providing healthcare in short-term custodial settings in Australia | Concept: doesn’t report on health, or determinants of health |
| Australian Institute of Criminology 2007 [20] | Reason for not buying drugs | Concept: doesn’t report on health, or determinants of health |
| Northern Territory Department of Health 2014 [21] | Report to the Minister for Alcohol Rehabilitation on the review of the Alcohol Mandatory Treatment Act (2013) | Concept: doesn’t report on health, or determinants of health |
| Skinns 2023 [22] | Researching inside police custody in four jurisdictions: 'Getting in', 'getting on', 'getting your hands dirty' and 'getting through it' | Concept: doesn’t report on health, or determinants of health |
| Government of Western Australia 2009 [23] | Response to the recommendations made by the State Coroner following the investigation into the death of Mr Ward | Concept: doesn’t report on health, or determinants of health |
| Office of Police Integrity 2011 [24] | Review of the investigative process following a death associated with a police contact | Concept: doesn’t report on health, or determinants of health |
| Payne & Langfield 2023 [25] | Screening for mental health and its correlates in criminal justice settings: is the inclusion of general personality and antisocial traits leading to biased estimation? | Concept: doesn’t report on health, or determinants of health |
| Gately et al., 2014 [26] | The 'oldest tricks in the book' don't work! Reports of burglary by DUMA detainees in Western Australia | Concept: doesn’t report on health, or determinants of health |
| Indig et al., 2011 [27] | 2009 NSW Young People in Custody Health Survey: Full Report | Context: not set in police custody in Australia |
| Jarvis et al., 2000 [28] | A client-centered model: discharge planning in Juvenile Justice Centres in New South Wales, Australia | Context: not set in police custody in Australia |
| Hicks 2003 [29] | A collaborative approach to the delivery of mental health services to juvenile offenders | Context: not set in police custody in Australia |
| Burns 2015 [30] | A longitudinal comparison of retention in buprenorphine and methadone treatment for opioid dependence in New South Wales, Australia | Context: not set in police custody in Australia |
| Baldry 2015 [31] | A predictable and preventable path: Aboriginal people with mental and cognitive disabilities in the criminal justice system | Context: not set in police custody in Australia |
| Templeton 2010 [32] | Aboriginal health worker screening for sexually transmissible infections and blood-borne viruses in a rural Australian juvenile correctional facility. | Context: not set in police custody in Australia |
| Standing Committee on Social Issues, Legislative Council NSW 2004 [33] | Alcohol and the justice system, in Report on the Inebriates Act 1912 | Context: not set in police custody in Australia |
| Eriksson et al., 2024 [34] | Committing Homicide After Drinking: The Characteristics of Self-Reported Alcohol-Involved Homicide Offending | Context: not set in police custody in Australia |
| McGovern et al., 2020 [35] | Containment and care? A qualitative interview study exploring police custody staff views about delivering brief alcohol interventions to heavy drinking arrestees | Context: not set in police custody in Australia |
| Morgan 2013 [36] | Directed review into an incident at Banksia Hill Detention Centre on 20 January 2013 | Context: not set in police custody in Australia |
| Office of the Inspector of Custodial Services 2007 [37] | Directed review into an incident at Rangeview Juvenile Remand Centre and its implications for management and reporting | Context: not set in police custody in Australia |
| Dawes 2002 [38] | Dying with dignity: Prisoners and terminal illness | Context: not set in police custody in Australia |
| Kesic et al., 2013 [39] | Estimated rates of mental disorders in, and situational characteristics of, incidents of nonfatal use of force by police | Context: not set in police custody in Australia |
| Anstis & Thomas 2022 [40] | Exploring the victim offender overlap among people with an intellectual disability | Context: not set in police custody in Australia |
| Laugalis & Connolly 2024 [41] | Head Injury and Antisocial Personality Features in a Sample of Juvenile Detainees | Context: not set in police custody in Australia |
| ACT Community Coalition on Corrections 2008 [42] | Healthy or harmful? Mental health and the operational regime of the new ACT prison | Context: not set in police custody in Australia |
| Watchirs et al., 2014 [43] | Human rights audit on the conditions of detention of women at the Alexander Maconochie Centre: a report by the ACT | Context: not set in police custody in Australia |
| Lansdell et al., 2018 [44] | 'I am not drunk, I have an ABI': findings from a qualitative study into systematic challenges in responding to people with acquired brain injuries in the justice system | Context: not set in police custody in Australia |
| Whitton & Indig 2012 [45] | Improving aboriginal Juvenile detainees’ access to community health services | Context: not set in police custody in Australia |
| Prichard & Payne 2005 [46] | Key findings from the Drug Use Careers of Juvenile Offenders study | Context: not set in police custody in Australia |
| Copeland et al., 2003 [47] | Patterns and correlates of substance use amongst juvenile detainees in New South Wales 1989 - 99 | Context: not set in police custody in Australia |
| Every-Palmer et al., 2023 [48] | Police, ambulance and psychiatric co-response versus usual care for mental health and suicide emergency callouts: A quasi-experimental study | Context: not set in police custody in Australia |
| Department of Health & Department of Justice Tasmania 2019 [49] | Prisoner Mental Health Care Taskforce: Final Report | Context: not set in police custody in Australia |
| Chappell 2004 [50] | Protecting the human rights of the mentally ill: Contemporary challenges for the Australian criminal justice system | Context: not set in police custody in Australia |
| Sarre et al., 2006 [51] | Remand in custody: Critical factors and key issues. | Context: not set in police custody in Australia |
| Office of the Inspector of Custodial Services, Western Australia 2003 [52] | Report of an announced inspection of Acacia Prison | Context: not set in police custody in Australia |
| Office of the Inspector of Custodial Services, Western Australia 2008 [53] | Report of an announced inspection of Rangeview Juvenile Remand Centre | Context: not set in police custody in Australia |
| Sentencing Advisory Council (Vic) 2010 [54] | Sentencing in the Koori Court Division of the Magistrates’ Court: A Statistical Report | Context: not set in police custody in Australia |
| Singh 2012 [55] | Substance use in a Victorian youth justice population | Context: not set in police custody in Australia |
| Lennings et al., 2003 [56] | Substance Use Patterns of Young Offenders and Violent Crime | Context: not set in police custody in Australia |
| D'Antoine et al., 2022 [57] | Suicidal behaviour in Aboriginal and non-Aboriginal young men under custodial youth justice supervision: understanding the role of adverse childhood experiences | Context: not set in police custody in Australia |
| O’Brien 2003 [58] | Surviving risk: juvenile justice workers’ perspectives on managing blood borne viruses and other health risks | Context: not set in police custody in Australia |
| Shepherd et al., 2017 [59] | The impact of indigenous cultural identity and cultural engagement on violent offending. | Context: not set in police custody in Australia |
| Passey et al., 2006 [60] | The Magistrates Early Referral into Treatment (MERIT) Pilot Program: A descriptive analysis of a court diversion program in rural Australia | Context: not set in police custody in Australia |
| Kenny & Lennings 2007 [61] | The relationship between head injury and violent offending in juvenile detainees | Context: not set in police custody in Australia |
| Richards et al., 2011 [62] | Trends in juvenile detention in Australia | Context: not set in police custody in Australia |
| Cockram 2005 [63] | People with an intellectual disability in the prisons | Population: the study did not report on police detainees |
| Dilip et al., 2001 [64] | Using Police as Custodial Officers: An Analysis From the Perspective of International Human Rights Standards | Population: the study did not report on police detainees |
| Buckman 2003 [65] | Youth justice: Criminal trajectories | Population: the study did not report on police detainees |
| Ferrante1999 [66] | Aboriginal involvement in the Western Australian criminal justice system: a statistical review | Publication date: pre-2000 |
| Crime and Misconduct Commission Queensland 2010 [67] | CMC review of the Queensland Police Service’s Palm Island review | Type of source: An investigation into a single death |
| Holcombe 2016 [68] | Human Rights, Colonial Criminality, and the Death of Kwementyaye Briscoe in Custody: A Central Australian Case Study | Type of source: An investigation into a single death |
| Klippmark & Crawley 2018 [69] | Justice for Ms Dhu: Accounting for Indigenous Deaths in Custody in Australia | Type of source: An investigation into a single death |
| Collins 2001 [70] | Australian Deaths in Custody and Custody-related Police Operations, 2000. (cover story) | Type of source: News article |
| Rogalla 2002 [71] | Caring for detainees -- an advocacy role for nurses. | Type of source: News article |
| Department of Justice Victoria 2004 [72] | Independent back up for young people in trouble | Type of source: News article |
| Ogden 2010 [73] | Showing care in custody | Type of source: News article |
| Rowse 2020 [74] | 'Cleared' as mud. Communication between health services and police: Risks to detainees in police custody | Type of source: Other This abstract was a description of a presentation, and did not include the information from the presentation. |
| Gaskin 2012 [75] | Mental health and trauma in young offenders-a New South Wales perspective | Type of source: Other This abstract was a description of a presentation, and did not include the information from the presentation. |
| Brouwer 2024 [76] | An overview of critical incidents deaths | Type of source: Other This abstract was a description of a presentation, and did not include the information from the presentation. |
| Chappell & Ryan 2017 [77] | Interactions between police and people with mental illness | Type of source: Other This article is an introductory piece in a medical journal to introduce one of the articles released in this edition. |
| Perera 2023 [78] | “Never Settler Enough”: The Double Economy of Terror and Deaths in Custody in Australia | Type of source: Summary of previously published data or a review article |
| Cunneen 2006 [79] | Aboriginal Deaths in Custody: A Continuing Systematic Abuse | Type of source: Summary of previously published data or a review article |
| Freckelton & List 2009 [80] | Asperger's Disorder, Criminal Responsibility and Criminal Culpability | Type of source: Summary of previously published data or a review article |
| Australin Institute of Criminology 2001 [81] | Australian heroin drought affects heroin market | Type of source: Summary of previously published data or a review article |
| Anthony 2021 [82] | Biopower of Colonialism in Carceral Contexts: Implications for Aboriginal Deaths in Custody | Type of source: Summary of previously published data or a review article |
| National Indigenous Drug and Alcohol Committee 2009 [83] | Bridges and barriers: addressing Indigenous incarceration and health | Type of source: Summary of previously published data or a review article |
| Williams 2001 [84] | Deaths in Custody: 10 Years on from the Royal Commission. (cover story) | Type of source: Summary of previously published data or a review article |
| Australian Institute of Criminology 2008 [85] | Deaths in custody: overview of trends, 1980-2006 | Type of source: Summary of previously published data or a review article |
| Australian Institute of Criminology 2006 [86] | Deaths in prison custody: sentenced and remand prisoners | Type of source: Summary of previously published data or a review article |
| Australian Institute of Criminology 2007 [87] | Drug use among police detainees across Australia | Type of source: Summary of previously published data or a review article |
| Mouzos & Smith 2006 [88] | Drug use among police detainees, 2005 | Type of source: Summary of previously published data or a review article |
| Sweeney & Payne 2012 [89] | Drug use among police detainees: A comparative analysis of DUMA and the US Arrestee Drug Abuse Monitoring program | Type of source: Summary of previously published data or a review article |
| Makkai 2001 [90] | Drug Use Amongst Police Detainees: Some Comparative Data | Type of source: Summary of previously published data or a review article |
| Milner 2004 [91] | Drug Use Monitoring in Australia | Type of source: Summary of previously published data or a review article |
| Everuss 2023 [92] | Everyday sovereign exclusion: Conceptualising police violence and deaths in custody as a racial production of homo sacer | Type of source: Summary of previously published data or a review article |
| Crilly et al., 2022 [93] | Health care in police watch-houses: a challenge and an opportunity | Type of source: Summary of previously published data or a review article |
| Bradford & Payne 2012 [94] | Illicit Drug Use and Property Offending among Police Detainees | Type of source: Summary of previously published data or a review article |
| Australian Institute of Criminology 2003 [95] | Indigenous deaths in custody in Australia | Type of source: Summary of previously published data or a review article |
| Behrendt 2013 [96] | Indigenous people and the criminal justice system | Type of source: Summary of previously published data or a review article |
| McCausland et al., 2017 [97] | Indigenous people, mental health, cognitive disability and the criminal justice system | Type of source: Summary of previously published data or a review article |
| Goldsmid & Willis 2016 [98] | Methamphetamine use and acquisitive crime: Evidence of a relationship. (cover story) | Type of source: Summary of previously published data or a review article |
| Fitzgerald & Chilvers 2002 [99] | Multiple drug use among police detainees | Type of source: Summary of previously published data or a review article |
| Walker et al., 2023 [100] | Police custody in Australia: A call for transparency and accountability | Type of source: Summary of previously published data or a review article |
| Ogloff 2013 [101] | Policing services with mentally ill people: developing greater understanding and best practice | Type of source: Summary of previously published data or a review article |
| McKinnon et al., 2023 [102] | Screening for mental disorders in police custody settings | Type of source: Summary of previously published data or a review article |
| Blue 2017 [103] | Seeing Ms. Dhu: inquest, conquest, and (in)visibility in black women’s deaths in custody | Type of source: Summary of previously published data or a review article |
| Australian Institute of Criminology 2014 [104] | Chapter 4: Selected offender profiles *In* Australian Crime: Facts & figures 2013 | Type of source: Summary of previously published data or a review article |
| Grant 2008 [105] | The case for single cells and alternative ways of viewing custodial accommodation for Australian Aboriginal peoples | Type of source: Summary of previously published data or a review article |
| Ogloff et al., 2007 [106] | The identification of mental disorders in the criminal justice system | Type of source: Summary of previously published data or a review article |
| Lin et al., 2020 [107] | Youth justice in Australia: Themes from recent inquiries | Type of source: Summary of previously published data or a review article |
| Cunneen 2009 [108] | Indigenous incarceration: The violence of colonial law and justice | Type of source: Summary of previously published data or a review article |
| Australasian Centre for Policing Research 2004 [109] | The apprehension and custodial care of offenders affected by the use of amphetamine type stimulants | Unable to be retrieved |

**Table 2: Studies from hand searches excluded at full text stage**

| **Study** | **Title** | **Primary Reason for Exclusion** |
| --- | --- | --- |
| Ogloff et al., 2007 [110] | The Identification of Mental Disorders in the Criminal Justice System: Criminology Research Council Consultancy | Concept: doesn’t report on health, or determinants of health in police custody |
| Parliament of NSW, 2021 [111] | The high level of First Nations people in custody and oversight and review of deaths in custody | Concept: doesn’t report on health, or determinants of health in police custody |
| Parliament of NSW, 2021 [112] | Select Committee on the High Level of First Nations People in Custody and Oversight and Review of Deaths in Custody (Government Response) | Concept: doesn’t report on health, or determinants of health |
| NSW State Coroner, 2021 [113] | Report by the NSW State Coroner into First Nations People’s Deaths in Custody in NSW 2008-2018 | Context: not set in police custody in Australia (none of the included deaths in custody occurred in police custody facilities, see section “facility where death occurred”. |
| Indig & Vecchiato 2009 [114] | Drug and alcohol use among juvenile detainees: findings from the 2009 NSW young people in custody health survey | Context: not set in police custody in Australia |
| Weatherburn et al., 2001 [115] | The Australian heroin drought and its implications for drug policy | Context: not set in police custody in Australia. In addition, some data was previously published. |

**References**

1. Crilly J, Polong-Brown J, Lincoln C, Timms J, Becker K, Scuffham P, et al. A structure and process evaluation of a police Watch House Emergency Nurse (WHEN) model of care. International emergency nursing. 2019;47(101472191):100790. doi: <https://dx.doi.org/10.1016/j.ienj.2019.100790>.

2. Lilburn S. Arresting moments : identifying risks for women and their children from the time of police arrest. Alternative law journal. 2001;26(3):115-8. PubMed PMID: cinch.251539.

3. Freckelton I. Attention Deficit Hyperactivity Disorder (ADHD) and the criminal law. Psychiatry Psychol Law. 2019;26(6):817-40. doi: 10.1080/13218719.2019.1695266. PubMed PMID: agispt.20200228026253.

4. Gray D, Saggers S, Atkinson D, Sputore B, Bourbon D. Beating the grog: an evaluation of the Tennant Creek liquor licensing restrictions. Australian and New Zealand journal of public health. 2000;24(1):39-44. doi: <https://dx.doi.org/10.1111/j.1467-842x.2000.tb00721.x>.

5. Cockram J. Careers of offenders with an intellectual disability: the probabilities of rearrest. Journal of Intellectual Disability Research. 2005;49(7):525-36. doi: 10.1111/j.1365-2788.2005.00707.x.

6. Hollander Y, Lee SJ, Tahtalian S, Young D, Kulkarni J. Challenges relating to the interface between crisis mental health clinicians and police when engaging with people with a mental illness. Psychiatry, Psychology and Law. 2012;19(3):402-11. doi: <https://dx.doi.org/10.1080/13218719.2011.585131>.

7. Toepfer S. City Watch House Community Nursing Service : the Clinical Practice Consultants’ response to homelessness in police holding cells. Parity. 2008;21(3):46. PubMed PMID: cinch.278934.

8. Baldry E, Dowse L. Compounding mental and cognitive disability and disadvantage: Police as care managers. Taylor and Francis; 2013. p. 219-34.

9. Johnson H, Burn G, Solarsh B, Lyon K, West D. Creating communication accessibility for people with communication disabilities at police stations. Journal of Intellectual Disability Research. 2019;63(7):701. doi: <https://dx.doi.org/10.1111/jir.12669>. PubMed PMID: 628696633.

10. McCarthy J, Chaplin E, Hayes S, Sondenaa E, Chester V, Morrissey C, et al. Defendants with intellectual disability and autism spectrum conditions: the perspective of clinicians working across three jurisdictions. Psychiatry, Psychology and Law. 2022;29(5):698EP-717. doi: <https://dx.doi.org/10.1080/13218719.2021.1976297>. PubMed PMID: 2014126037.

11. Crilly J, Lincoln C, Scuffham P, Byrnes J, Timms J, Becker K, et al. Effect of a 24/7 nursing presence in a police watch house on police presentations to the emergency department. Australian health review : a publication of the Australian Hospital Association. 2020;44(6):924-30. doi: <https://dx.doi.org/10.1071/AH19294>.

12. Victorian Law Reform Commission. Family violence police holding powers: interim report. Melbourne: Victorian Law Reform Commission, 2005.

13. Goldsmid S. Findings from the DUMA program : Internet access, and frequency and nature of use among police detainees2015.

14. Tasmania Law Reform Institute. Intoxication and criminal responsibility. Hobart: Tasmania Law Reform Institute; 2006.

15. Bartels L. Knife crime: Recent data on carriage and use. Woden: Australian Institute of Criminology; 2011. p. 1-6.

16. Payne J, Wong G. Measuring drug dependence in police custody: An item response theory and differential item function analysis of UNCOPE in Australia. Drug and alcohol review. 2018;37(7):856-64. doi: <https://dx.doi.org/10.1111/dar.12854>.

17. Fogarty B. Mental health: Police detention of people who appear mentally ill or mentally disturbed. LSJ: Law Society of NSW Journal. 2016;(20):72-4. PubMed PMID: ielapa.891573171683682.

18. Baldry E. People with intellectual and other cognitive disability in the criminal justice system : report for NSW Family and Community Services, Ageing, Disability and Home Care / Eileen Baldry, Leanne Dowse and Melissa Clarence2012.

19. Brandenburg C, Crilly J, Thomas S, Gardiner P, Kinner SA, Heffernan E, et al. Police perspectives on the economic considerations of providing healthcare in short-term custodial settings in Australia. Medicine, science, and the law. 2024;64(3):217-23. doi: <https://dx.doi.org/10.1177/00258024231198915>.

20. Australian Institute of Criminology. Reason for not buying drugs. Crime Facts Info [Internet]. 2007; 152. Available from: <https://www.aic.gov.au/publications/cfi/cfi152>.

21. Northern Territory Department of Health. Report to the Minister for Alcohol Rehabilitation on the review of the Alcohol Mandatory Treatment Act. Darwin: Northern Territory Department of Health, 2014.

22. Skinns L. Researching inside police custody in four jurisdictions: 'Getting in', 'getting on', 'getting your hands dirty' and 'getting through it'. Criminology & Criminal Justice: An International Journal. 2023;23(2):273-89. doi: <https://dx.doi.org/10.1177/17488958221087491>.

23. Government of Western Australia. Response to the recommendations made by the State Coroner following the investigation into the death of Mr Ward. Perth: Government of Western Australia, 2009.

24. Office of Police Integrity (Vic). Review of the investigative process following a death associated with a police contact. Melbourne: Victorian Government Printer; 2011.

25. Payne JL, Langfield CT. Screening for mental health and its correlates in criminal justice settings: is the inclusion of general personality and antisocial traits leading to biased estimation? J Crime Justice. 2023;46(4):526-42. doi: 10.1080/0735648X.2023.2174577.

26. Gately N, Fleming J, McGinty N, Scott A. The 'oldest tricks in the book' don't work! Reports of burglary by DUMA detainees in Western Australia. Woden: Australian Institute of Criminology; 2014. p. 1-9.

27. Indig D, Vecchiato C, Haysom L, Beilby R, Carter J, Champion U, et al. 2009 NSW Young People in Custody Health Survey: Full Report. Sydney: Justice Health and Juvenile Justice, 2011.

28. Jarvis LA, Beale B, Martin K. A client-centered model: discharge planning in Juvenile Justice Centres in New South Wales, Australia. International nursing review. 2000;47(3):184-90. doi: <https://dx.doi.org/10.1046/j.1466-7657.2000.00026.x>.

29. Hicks S. A collaborative approach to the delivery of mental health services to juvenile offenders. 2003.

30. Burns L, Gisev N, Larney S, Dobbins T, Gibson A, Kimber J, et al. A longitudinal comparison of retention in buprenorphine and methadone treatment for opioid dependence in New South Wales, Australia. Addiction. 2015;110(4):646-55. doi: 10.1111/add.12834.

31. Baldry E. A predictable and preventable path : Aboriginal people with mental and cognitive disabilities in the criminal justice system2015.

32. Templeton DJ, Tyson BA, Meharg JP, Habgood KE, Bullen PM, Malek S, et al. Aboriginal health worker screening for sexually transmissible infections and blood-borne viruses in a rural Australian juvenile correctional facility. Sexual health. 2010;7(1):44-8. doi: <https://dx.doi.org/10.1071/SH09035>.

33. Standing Committee on Social Issues, Legislative Council NSW. Report on the Inebriates Act 1912. 2004.

34. Eriksson L, Mazerolle P, McPhedran S, Wortley R. Committing Homicide After Drinking: The Characteristics of Self-Reported Alcohol-Involved Homicide Offending. J Aggress Maltreat Trauma. 2024;33(12):1437-57.

35. McGovern R, Crowe L, Addison M, Hickman M, Kidger J, McColl E, et al. Containment and care? A qualitative interview study exploring police custody staff views about delivering brief alcohol interventions to heavy drinking arrestees. Drugs Educ Prev Policy. 2020;27(6):436-44.

36. Morgan N. Directed review into an incident at Banksia Hill Detention Centre on 20 January 2013. Perth (Australia): Office of the Inspector of Custodial Services; 2013 Jul.

37. Office of the Inspector of Custodial Services. Directed review into an incident at Rangeview Juvenile Remand Centre and its implications for management and reporting. Perth (Australia): Office of the Inspector of Custodial Services; 2007 Apr.

38. Dawes J. Dying with dignity: Prisoners and terminal illness. Illn Crisis Loss. 2002;10(3):188-203. doi: <https://dx.doi.org/10.1177/1054137302010003002>.

39. Kesic D, Thomas SDM, Ogloff JRP. Estimated rates of mental disorders in, and situational characteristics of, incidents of nonfatal use of force by police. Soc Psychiatry Psychiatr Epidemiol. 2013;48(2):225-32. doi: <https://dx.doi.org/10.1007/s00127-012-0543-4>.

40. Anstis S, Thomas SDM. Exploring the victim offender overlap among people with an intellectual disability. J Appl Res Intellect Disabil. 2022;35(3):789-99. doi: 10.1111/jar.12984.

41. Laugalis V, Connolly EJ. Head Injury and Antisocial Personality Features in a Sample of Juvenile Detainees. Youth Violence Juven Justice. 2024;22(1):46-60. doi: 10.1177/15412040231181349.

42. ACT Community Coalition on Corrections Act. Healthy or harmful?: mental health and the operational regime of the new ACT prison. Canberra (Australia): ACT Community Coalition on Corrections; 2008 Apr.

43. Watchirs H, McKinnon G, Costello S, Thomson J, Hobbs H. Human rights audit on the conditions of detention of women at the Alexander Maconochie Centre: A report by the ACT Human Rights and Discrimination Commissioner. Canberra (Australia): ACT Human Rights Commission; 2014 Apr.

44. Lansdell G, Saunders B, Eriksson A, Bunn R, Baidawi S. 'I am not drunk, I have an ABI': findings from a qualitative study into systematic challenges in responding to people with acquired brain injuries in the justice system. Psychiatry Psychol Law. 2018;25(5):737-58. doi: <https://dx.doi.org/10.1080/13218719.2018.1474818>. PubMed PMID: 622613723.

45. Whitton N, Indig D. Improving aboriginal Juvenile detainees’ access to community health services. Aborig Isl Health Work J. 2012;36(1):13-7. PubMed PMID: ielapa.369350998485050.

46. Prichard J, Payne J. Key findings from the Drug Use Careers of Juvenile Offenders study. Canberra (Australia): Australian Institute of Criminology; 2005 Oct. 1-6 p.

47. Copeland JAN, Howard J, Keogh TIM, Seidler K. Patterns and correlates of substance use amongst juvenile detainees in New South Wales 1989-99. Drug Alcohol Rev. 2003;22(1):15-20. doi: 10.1080/0959523021000059785.

48. Every-Palmer S, Kim AHM, Cloutman L, Kuehl S. Police, ambulance and psychiatric co-response versus usual care for mental health and suicide emergency callouts: A quasi-experimental study. Aust N Z J Psychiatry. 2023;57(4):572-82. doi: <https://dx.doi.org/10.1177/00048674221109131>.

49. Department of Health & Department of Justice. Prisoner Mental Health Care Taskforce. Hobart (Australia): Office of the Chief Psychiatrist; 2019 Mar.

50. Chappell D. Protecting the human rights of the mentally ill: Contemporary challenges for the Australian criminal justice system. Psychiatry Psychol Law. 2004;11(1):13-22. doi: <https://dx.doi.org/10.1375/1321871041335993>. PubMed PMID: 38917408.

51. Sarre R, King S, Bamford D. Remand in custody: Critical factors and key issues. Canberra (Australia): Australian Institute of Criminology; 2006 May. 1-6 p.

52. Office of the Inspector of Custodial Services. Report of an announced inspection of Acacia Prison. Perth (Australia): Office of the Inspector of Custodial Services, Western Australia; 2003 Mar.

53. Office of the Inspector of Custodial Services. Report of an announced inspection of Rangeview Juvenile Remand Centre. Perth (Australia): Office of the Inspector of Custodial Services; 2008 Apr.

54. Sentencing Advisory Council (Vic). Sentencing in the Koori Court division of the Magistrates’ court: A statistical report. Melbourne (Australia): State of Victoria; 2010 Oct.

55. Singh Y. Substance use in a victorian youth justice population. Aust N Z J Psychiatry. 2012;46(SUPPL. 1):55-6. doi: <https://dx.doi.org/10.1177/0004867412445952>. PubMed PMID: 71047105.

56. Lennings CJ, Copeland J, Howard J. Substance Use Patterns of Young Offenders and Violent Crime. Aggress Behav. 2003;29(5):414-22. doi: <https://dx.doi.org/10.1002/ab.10048>.

57. D'Antoine M, Malvaso C, Delfabbro P, O'Connor J. Suicidal behaviour in Aboriginal and non-Aboriginal young men under custodial youth justice supervision: understanding the role of adverse childhood experiences. Psychiatry Psychol Law. 2022;29(6):953-75. doi: <https://dx.doi.org/10.1080/13218719.2021.2003263>. PubMed PMID: 2015074311.

58. O’Brien M. Surviving risk: Juvenile justice workers’ perspectives on managing blood borne viruses and other health risks. Youth Stud Aust. 2003;22(3):25-31. PubMed PMID: cinch.260897.

59. Shepherd SM, Delgado RH, Sherwood J, Paradies Y. The impact of indigenous cultural identity and cultural engagement on violent offending. BMC Public Health. 2017;17:1-7. doi: 10.1186/s12889-017-4603-2.

60. Passey M, Flaherty B, Didcott P. The Magistrates Early Referral into Treatment (MERIT) Pilot Program: A descriptive analysis of a court diversion program in rural Australia. J Psychoactive Drugs. 2006;38(4):521-9. doi: <http://dx.doi.org/10.1080/02791072.2006.10400591>. PubMed PMID: 46340097.

61. Kenny DT, Lennings CJ. The relationship between head injury and violent offending in juvenile detainees. Sydney (Australia): NSW Bureau of Crime Statistics and Research; 2007 Mar.

62. Richards K. Trends in juvenile detention in Australia. Canberra (Australia): Australian Institute of Criminology; 2011. 1-8 p.

63. Cockram J. People with an intellectual disability in the prisons. Psychiatry Psychol Law. 2005;12(1):163-73. PubMed PMID: cinch.269153.

64. Das DK, Light SC, Verma A. Using Police as Custodial Officers: An Analysis From the Perspective of International Human Rights Standards. Police Quarterly. 2001;4(2):215. doi: 10.1177/109861101129197806.

65. Lynch M, Buckman J, Krenske L. Youth Justice: Criminal trajectories. Canberra (Australia): Australian Institute of Criminology; 2003 Sep.

66. Ferrante A. Aboriginal involvement in the Western Australian criminal justice system : A statistical review. Perth (Australia): The University of Western Australia; 1999.

67. Crime and Misconduct Commission. CMC review of the Queensland Police Service’s Palm Island review. Brisbane (Australia): Crime and Misconduct Commission; 2010 Jun.

68. Holcombe S. Human Rights, Colonial Criminality, and the Death of Kwementyaye Briscoe in Custody: A Central Australian Case Study. Polit Leg Anthropol Rev. 2016;39:104-20. doi: 10.1111/plar.12174.

69. Klippmark P, Crawley K. Justice for Ms Dhu: Accounting for Indigenous Deaths in Custody in Australia. Soc Leg Stud. 2018;27(6):695-715. doi: 10.1177/0964663917734415.

70. Collins L, Mouzos J. Australian Deaths in Custody and Custody-related Police Operations, 2000. (cover story). Trends & Issues in Crime & Criminal Justice. 2001;(217):1-6.

71. Rogalla B. Caring for detainees - an advocacy role for nurses. ACCNS Journal for Community Nurses. 2002;7(1):9-.

72. Department of Justice Victoria. Independent back up for young people in trouble. Justice review. 2004;1(5, Sep):6. PubMed PMID: cinch.265866.

73. Ogden E. Showing care in custody. Of substance. 2010;8(1):17. PubMed PMID: cinch.284612.

74. Rowse J. 'Cleared' as mud. Communication between health services and police: Risks to detainees in police custody. Pathology. 2020;52(Supplement 1):S22. doi: <https://dx.doi.org/10.1016/j.pathol.2020.01.102>. PubMed PMID: 2004694061.

75. Gaskin C. Mental health and trauma in young offenders-a New South Wales perspective. Neuropsychiatrie de l'Enfance et de l'Adolescence. 2012;60(5 SUPPL. 1):S138. doi: <https://dx.doi.org/10.1016/j.neurenf.2012.04.104>. PubMed PMID: 71880273.

76. Brouwer I, Maistry S. An overview of critical incidents deaths. Pathology. 2024;56(Supplement 1):S12-S3. doi: <https://dx.doi.org/10.1016/j.pathol.2023.12.050>. PubMed PMID: 2030058558.

77. Chappell D, Ryan CJ. Interactions between police and people with mental illness. Aust N Z J Psychiatry. 2017;51(4):409-10. doi: 10.1177/0004867416682833.

78. Perera S, Pugliese J. “Never Settler Enough”: The Double Economy of Terror and Deaths in Custody in Australia. Filoz Vestn. 2023;44(2):307-28. doi: 10.3986/fv.44.2.14.

79. Cunneen C. Aboriginal Deaths in Custody: A Continuing Systematic Abuse. Social Justice. 2006;33(4):37-51. PubMed PMID: 231923235.

80. Freckelton I, List D. Asperger's Disorder, Criminal Responsibility and Criminal Culpability. Psychiatry Psychol Law. 2009;16(1):16-40. doi: 10.1080/13218710902887483.

81. Australian heroin drought affects heroin market. Canberra (Australia): Australian Institute of Criminology; 2001 Nov.

82. Anthony T, Blagg H. Biopower of Colonialism in Carceral Contexts: Implications for Aboriginal Deaths in Custody. J Bioeth Inq. 2021;18(1):71-82. doi: <https://dx.doi.org/10.1007/s11673-020-10076-x>.

83. National Indigenous Alcohol and Drug Committee. Bridges and barriers: Addressing Indigenous incarceration and health. Canberra (Australia): Australian National Council on Drugs; 2009 Jun.

84. Williams P. Deaths in Custody: 10 Years on from the Royal Commission. (cover story). Trends & Issues in Crime & Criminal Justice. 2001;(203):1-6.

85. Australian Institute of Criminology. Deaths in custody: overview of trends, 1980-2006. Crime Facts Info. 2008;166.

86. Australian Institute of Criminology. Deaths in prison custody: Sentenced and remand prisoners. Crime Facts Info. 2006;114.

87. Australian Institute of Criminology. Drug use among police detainees across Australia. Canberra (Australia): Australian Institute of Criminology; 2007.

88. Mouzos J, Smith L. Drug use among police detainees, 2005. Trends & Issues in Crime & Criminal Justice. 2006;(319):1-6.

89. Sweeney J, Payne J. Drug use among police detainees: a comparative analysis of DUMA and the US Arrestee Drug Abuse Monitoring program. Canberra (Australia): Australian Institute of Criminology; 2012 May.

90. Makkai T. Drug Use Amongst Police Detainees: Some Comparative Data. Trends & Issues in Crime and Criminal Justice. 2001;191:1-6.

91. Milner L. Drug Use Monitoring in Australia. Of Substance. 2004;2(4):23-4.

92. Everuss L. Everyday sovereign exclusion: Conceptualising police violence and deaths in custody as a racial production of homo sacer. Distinktion: Journal of Social Theory. 2023;24(3):383-404. doi: 10.1080/1600910X.2023.2220933.

93. Crilly JL, Brandenburg C, Kinner SA, Heffernan E, Byrnes J, Lincoln C, et al. Health care in police watch-houses: a challenge and an opportunity. Med J Aust. 2022. doi: <https://dx.doi.org/10.5694/mja2.51688>.

94. Bradford D, Payne J. Illicit Drug Use and Property Offending among Police Detainees. Contemporary Issues in Crime and Justice. 2012;(157):1-12.

95. Australian Institute of Criminology. Indigenous deaths in custody in Australia. Canberra (Australia): Australian Institute of Criminology; 2003 Aug.

96. Behrendt L. Indigenous people and the criminal justice system. Hot Topics: Legal Issues in Plain Language. 2013;(86):7-10.

97. McCausland R, McEntyre E, Baldry E. Indigenous people, mental health, cognitive disability and the criminal justice system. Indigenous Justice Clearinghouse. 2017;22.

98. Goldsmid S, Willis M. Methamphetamine use and acquisitive crime: Evidence of a relationship. (cover story). Trends & Issues in Crime & Criminal Justice. 2016;(516):1-14.

99. Fitzgerald J, Chilvers M. Multiple drug use among police detainees. Sydney (Australia): NSW Bureau of Crime Statistics and Research; 2002 Jan.

100. Walker S, Wilson M, Seear K, Doyle M, Saich F, Stoove M, et al. Police custody in Australia: A call for transparency and accountability. Australian and New Zealand journal of public health. 2023;47(2):100040. doi: <https://dx.doi.org/10.1016/j.anzjph.2023.100040>.

101. Ogloff JRP. Policing services with mentally ill people: Developing greater understanding and best practice. Australian Psychologist. 2013;48(1):57-68.

102. McKinnon I, Moore J, Lyall A, Forrester A. Screening for mental disorders in police custody settings. BJPsych Adv. 2023;29(6):407-16. doi: <https://dx.doi.org/10.1192/bja.2022.25>. PubMed PMID: 2028578122.

103. Blue E. Seeing Ms. Dhu: Inquest, conquest, and (in)visibility in black women’s deaths in custody. Settler Colon Stud. 2017;7(3):299-320. doi: 10.1080/2201473X.2016.1229294.

104. Australian Institute of Criminology. Chapter 4: Selected offender profiles. Australian Crime: Facts & Figures 2013. Canberra (Australia): Australian Institute of Criminology; 2013. p. 60-83.

105. Grant E. The case for single cells and alternative ways of viewing custodial accommodation for Australian Aboriginal peoples. Flinders journal of law reform. 2008;10(3):631-47. PubMed PMID: cinch.280138.

106. Ogloff JRP, Davis MR, Rivers G, Ross S. The identification of mental disorders in the criminal justice system. Trends & Issues in Crime & Criminal Justice. 2007;(334):1-6.

107. Lin B, Clancey G, Wang S. Youth justice in Australia: Themes from recent inquiries. Trends and Issues in Crime and Criminal Justice [electronic resource]. 2020;(605):1-19. PubMed PMID: agispt.20201215041190.

108. Cunneen C. Indigenous incarceration: The violence of colonial law and justice. In: Scraton P, McCulloch J, editors. The violence of incarceration. New York, NY, US: Routledge Taylor & Francis Group; 2009. p. 209-24.

109. Australian Centre for Policing Research. The apprehension and custodial care of offenders affected by the use of amphetamine type stimulants2004.

110. Ogloff JRP, Davis MR, Rivers G, Ross S. The Identification of Mental Disorders in the Criminal Justice System: Criminology Research Council Consultancy. Melbourne (Australia): Centre for Forensic Behavioural Science & Monash University; 2007.

111. Parliament of NSW. The high level of First Nations people in custody and oversight and review of deaths in custody. Sydney (Australia): Parliament of New South Wales; 2021 Apr.

112. Parliament of NSW. The high level of First Nations people in custody and oversight and review of deaths in custody (Government Response). Sydney (Australia): Parliament of New South Wales; 2021.

113. NSW State Coroner. Report by the NSW State Coroner into deaths in Custody 2008-2018. Sydney (Australia): NSW Office of the State Coroner; 2021 Mar.

114. Indig D, Vecchiato CA. Drug and alcohol use among juvenile detainees: Findings from the 2009 NSW young people in custody health survey. Darwin: Australasian Professional Society on Alcohol and other Drugs; 2009.

115. Weatherburn D, Jones C, Freeman K, Makkai T. The Australian Heroin Drought and its Implications for Drug Policy. Sydney (Australia): NSW Bureau of Crime Statistics and Research; 2001 Oct.
