# Supplementary material for: The determinants of health and health status of individuals in police custody in Australia: A scoping review
Source: PLoS One. 2025 Dec 30;20(12):e0338957. doi: 10.1371/journal.pone.0338957 (PMC12753082; doi:10.1371/journal.pone.0338957)
Supplement: S6 Appendix — This appendix includes two tables which contain the characteristics of all included academic and grey literature information sources. This includes each information source’s jurisdiction of data collection, study design, sample size, aim, methods and conclusions. (DOCX) [file pone.0338957.s006.docx]

# **S6 Appendix: Included Information Sources**

[**Table 1: Academic Literature** 2](#_Toc200636622)

[**Table 2: Grey Literature** 21](#_Toc200636623)

[**References** 84](#_Toc200636624)

## **Table 1: Academic Literature**

| **Study** | **Title** | **Jurisdiction** | **Population** | **Study Design** | **Sample Size** | **Aim** | **Methods** | **Conclusions** |
| --- | --- | --- | --- | --- | --- | --- | --- | --- |
| Petschel & Gall 2000 [1] | A profile of deaths in custody in Victoria, 1991–96 | VIC | Individuals that died in custody in Victoria between January 1991 and December 1996. | Retrospective analysis of routinely collected data | 96 deaths (45 in PC) | To create a profile of the deaths that occurred in custody in Victoria from 1991 to 1996. | A retrospective quantitative analysis of data on deaths in custody from the Victorian Institute of Forensic Medicine database. | Accidental deaths and suicide remain high and are the cause of most custodial deaths in Victoria and elsewhere. |
| Makkai 2001 [2] | Patterns of recent drug use among a sample of Australian detainees | NSW, QLD, WA | Adult male police detainees held in one of the four study site locations who had been in detention for less than 48 hours. | Cross-sectional Study | 1179 | To ascertain the prevalence of recent drug use among police detainees. | Structured interviews using a questionnaire were used to collect information on detainee demographics and recent drug use. Urinalysis was also completed. | To effectively monitor patterns of drug use among at-risk populations, such as detainees, data collections grounded at the local level are necessary. |
| Heffernan et al., 2003 [3] | Substance-use disorders and psychological distress among police arrestees | QLD | Police detainees in custody for more than 12 hours at the Brisbane City Police Watch House in February and March 2001. | Cross-sectional Study | 288 | To determine the 12-month prevalence of substance-use disorders and psychological morbidity in an Australian arrestee population. | Researchers conducted interviews with eligible detainees and utilised two survey tools. The CIDI-Auto Core version 2.1 and the 28-item General Health Questionnaire. | Development of services for detoxification and treatment of this population is a pressing need. The findings provide crucial information for the planning and implementation of drug courts and court diversion systems. |
| Taylor et al., 2003 [4] | Monitoring the use of illicit drugs in four countries through the International Arrestee Drug Abuse Monitoring (I-ADAM) program | NSW, QLD, WA | Adult male arrestees in the USA, Australia, South Africa and England (only data relating to Australia extracted) | Cross-sectional Study | 2121 | To use I-ADAM data for 2000 from four countries (Australia, England, South Africa and the United States) to examine the rates of detainees testing positive for drugs and arrestee self-reports of illicit drug use in the past 12 months. | Data was collected using interviews and undertaking urinalysis. Quantitative analysis was completed. | There is a considerable amount of drug use across all four countries in the arrestee population. About half of all the arrestees in all four countries test positive for recent three-day marijuana use. |
| Brewer & Dalton 2008 [5] | Assessing the Importance of Culture in Explaining Drug Use amongst Indigenous Police Detainees in Adelaide | SA | Indigenous individuals in police custody in an urban setting in South Australia. | Cross-sectional Study | 307 | To consider and discuss a range of social, internal and environmental risk factors which have been identified within the literature as relating to the early adoption of drug using behaviours amongst both Indigenous and non-Indigenous  Australians. | Researchers reviewed relevant data collected from the DUMA sites in South Australia in 2004 and 2005 to assess whether Indigenous participants met DSM-IV criteria for drug dependence. | Early adoption of drug use amongst Indigenous police detainees may relate to an increased likelihood of sustained use and future dependence. |
| Griffiths et al., 2009 [6] | Sex differences in drug use and offending at the East Perth Watch House | WA | Adult police detainees held at East Perth Watch House between 1999 and 2007 who participated in the DUMA program and who provided a urine sample. | Retrospective analysis of routinely collected data | 4144 | To investigate the relationship between violent offending and illicit drug use in detainees. | Retrospective quantitative analysis of a subset of DUMA program data. | Offending behaviour differed between sexes. Females were more likely to be detained for a violent offense than male detainees, and more likely to test positive to an illicit drug within 48 hours of being detained. |
| Baksheev et al., 2010 [7] | Psychiatric disorders and unmet needs in Australian police cells | VIC | Police detainees in two metropolitan police stations in Melbourne, Australia. | Mixed Methods (Cross-sectional and Data Linkage) | 150 | To determine the prevalence of current psychiatric disorders and unmet needs in a sample of police cell detainees in Victoria. | Detainees were interviewed using the Structured Clinical Interview for DSM-IV-TR to assess for mental conditions in the last month, and the Camberwell Assessment of Need - Forensic Short Version was used to measure individual needs. Data linkage was also completed by accessing the state-wide public mental health database. | The current study highlights the significant psychiatric morbidity found in the police cell population, and the pressing need to evaluate standardized screening tools for mental illnesses to provide timely access to assessment and treatment services. |
| Sturgiss & Parekh 2010 [8] | The work of forensic physicians with police detainees in the Canberra City Watchhouse | ACT | Police detainees at the Canberra City Watchhouse that were seen by the Clinical Forensics ACT service. | Retrospective analysis of routinely collected data | 726 | To determine the characteristics of detainees seen by forensic physicians in the Canberra City Watchhouse, and the type of work performed by forensic physicians. | A retrospective clinical audit was conducted by forensic physicians working at Clinical Forensics ACT. Routinely entered data from consultations was reviewed and analysed quantitatively. | More than 50% of the detainees seen by the service were under the age of 30. Some individuals had more than 4 episodes of care recorded, with fourteen individuals accounting for 17.2% of the workload for the 2-year study period. Most of the workload in the Watchhouse involves medical review of detainees and not forensic procedures. As many detainees gave histories of mental health and/or drug and alcohol problems, it is essential that forensic physicians are skilled in these areas. |
| Ogloff et al., 2011 [9] | Psychiatric symptoms and histories among people detained in police cells | VIC | Individuals in police custody in one of nine police stations, in both rural and metropolitan Victoria, Australia. | Mixed Methods (Cross-sectional and Data Linkage) | 614 | To examine the psychiatric symptoms, mental health histories and psychiatric medication used in a sample of people detained in police cells. | Clinical interviews of detainees were completed, and the Brief Psychiatric Rating Scale was used to measure psychiatric symptoms. | Psychiatric symptoms experienced by people detained in police cells are common and present a considerable dilemma for police agencies which are generally ill equipped to address them. |
| Baksheev et al., 2011 [10] | Identification of mental illness in police cells: a comparison of police processes, the Brief Jail Mental Health Screen and the Jail Screening Assessment Tool | VIC | Adult police detainees in one of two busy metropolitan police stations in Melbourne between May 2008 and February 2009. Participants were excluded if they transferred from prison awaiting court, if they didn't speak English, or if they were detained solely for public drunkenness. | Cross-sectional Study | 150 | To investigate current police practices employed to identify those with a mental illness in police custody, and to evaluate the predictive utility of the BJMHS and the JSAT. | The Structured Clinical Interview for DSM-IV-TR was used to identify psychiatric disorders. Findings from the clinical interview were compared to the assessment made by police officers, and to the results from two screening tools: the BJMHS, and the JSAT. | There is a pressing need to further investigate standardised screening tools for mental illnesses in police cells, with an aim of introducing their application across services in  police custody. Such an introduction would assist in identifying those who require mental health care in custody and linking into services either in prison or the community. |
| Baksheev et al., 2012 [11] | Correlates of criminal victimisation among police cell detainees in Victoria, Australia | VIC | Police detainees at one of nine data collection sites in Victoria, between December 2006 and February 2009. | Mixed Methods (Cross-sectional and Data Linkage) | 764 | To investigate the rates and types of victimisation among people detained in police cells, with and without histories of mental illness. | Detainees were enrolled and surveyed while in police custody, and if permission was granted, further data was obtained through data linkage to participants police records and public mental health records. | It was found that the overwhelming majority (almost three quarters of the detainees) had reported to police that they had been the victim of a criminal offence. The most common victimisation experiences were for theft, violent crimes and property damage. |
| Baksheev et al., 2012 [12] | Psychopathology in Police Custody: The Role of Importation, Deprivation and Interaction Models | VIC | Adult police detainees in one of two busy metropolitan police stations in Melbourne between May 2008 and February 2009. Participants were excluded if they transferred from prison awaiting court, if they didn't speak English, or if they were detained solely for public drunkenness. | Mixed Methods (Cross-sectional and Data Linkage) | 150 | To examine the predictive power of personal factors (e.g., history of psychiatric hospitalisation), situational factors (e.g., police cell conditions), and their interactive effects to explain the occurrence of psychopathology in police custody. | Interviews were conducted with detainees. The JSAT was used to measure psychiatric symptomatology, social desirability bias was measured using the Marlowe-Crowne Social Desirability Scale, psychiatric disorders were assessed using the Structured Clinical Interview for DSM-IV-TR, and the physical conditions of cells were measured using the Checklist of Physical Conditions in Police Cells. Interview data was also linked to mental healthcare records from a statewide database. | Findings from the current study supported the importation model and provided partial support for the deprivation model in accounting for the occurrence of psychopathology among police cell detainees. |
| Marchetti 2012 [13] | Victims or offenders: Who were the 11 Indigenous female prisoners who died in custody and were investigated by the Australian Royal Commission into Aboriginal Deaths in Custody? | WA, QLD, SA | The primary focus population is deceased females in the RCIADIC, however comparative information on the deceased males is also presented. | Qualitative Study | 11 deaths, 9 of which were in police custody | To explore how the 11 deceased females in the RCIADIC became invisible as victims and offenders in favour of the Indigenous males. | A thematic content analysis of the death reports of the 11 females included in the RCIADIC combined with interviews with 48 people either involved in the RCIADIC or who worked in the Aboriginal Issues Units established from the enquiry. | The RCIADIC’s treatment of the problems concerning Indigenous women, particularly those that were detrimental to their safety, was inadequate. Although there were many procedural constraints placed upon the RCIADIC’s ability to conduct its inquiry, there is evidence to suggest that other inquiries, such as the Aboriginal Justice Inquiry of Manitoba, were able to surpass similar constraints and adopt an intersectional race and gender approach. |
| Swan & Goodman-Delahunty 2013 [14] | The Relationship between Drug Use and Crime among Police Detainees: Does Gender Matter? | NSW | Female police detainees in NSW at DUMA data collection sites between 1999 and 2005. | Retrospective analysis of routinely collected data | 867 | To examine the temporal order of substance use and offending and other correlates between substance use and offending in women who had been arrested by police and were confined in police custody. | Data collected through the DUMA program in NSW was analysed to assess the relationship between substance use and criminal offending using canonical correlation analysis. | Overall, the findings on substance use and offending for female police detainees revealed that substance use preceded crime for female offenders, and that other factors such as age and education influenced patterns of drug use and offending. |
| Porter 2013 [15] | Indigenous deaths associated with police contact in Australia: Event stages and lessons for prevention | WA, NT, NSW, SA, QLD, VIC, TAS | Indigenous individuals who died in police custody or custody-related operations between 1992 and 2008. | Content Analysis | 98 | To analyse factors involved in Indigenous deaths associated with police contact in Australia since the Royal Commission, with a view to identifying possible points of prevention. | Coroner reports were analysed for individual and situational variables, as well as recommendations. Variables are reported around a framework of the stages of the incident from the initial information or contact through to an officer decision to detain and subsequent after care. Subsets are detailed by cause of death. | The study showed that, in many cases, officers received information at the initial stages that warranted a response of detention, with a variety of requests for service from the public concerning potential endangerment and offending. However, there were also many cases where the situations that police encountered could have resulted in less serious courses of action, with the provision of alternatives to arrest, pursuit or police custody. |
| Simpson 2013 [16] | Chapter 3: Age, drug use and crime among police detainees *In* Caught red-eyed and red-handed: An exploration of cannabis use and criminal offending | NSW | Police detainees in NSW DUMA program data collection sites between 2006 and 2010. | Retrospective analysis of routinely collected data | 3570 | To examine the relationship between age, drug use, among those who come into contact with the criminal justice system. | A retrospective quantitative analysis of DUMA program data by age groups. | This study found a number of differences in drug use and offending between older and younger police detainees and detainees who fall into specific drug-user types. The findings highlight that cannabis remains a prominent drug in the lives of people who come into contact with the criminal justice system, particularly among young people who initiate use, use regularly at a younger age and whose past 12 months use is limited to cannabis. |
| Trofimovs & Dowse 2014 [17] | Mental health at the intersections: The impact of complex needs on police contact and custody for Indigenous Australian men | NSW | A cohort of Indigenous men with cognitive disabilities in NSW. | Retrospective analysis of routinely collected data | 131 | To explores patterns of police contact and custody for a cohort of Indigenous males with complex needs. | An analysis of linked administrative data compiled by University of NSW staff - the Mental Health Disorders and Cognitive Disability dataset. | This study has demonstrated that the presence of co-occurring mental illness fails to explain high levels of CJS contact for Indigenous Australian men with complex needs. So, whilst mental ill health may be one intersecting factor in a compound picture of disadvantage, the presence of an inter-locking range of social, material and individual factors is more crucial in predicting police contact and custody. |
| McArdle et al., 2017 [18] | Two cases of benign pneumomediastinum in patients with psychosis who had been restrained in police custody | TAS | Two patients presenting with acute psychosis and pneumomediastinum after having been restrained in police custody. | Case Report | 2 | To report on two instances of pneumomediastinum occurring in individuals in police custody following restraint. | Review of clinical notes. | N/A |
| Crissman 2019 [19] | Deaths of people with serious mental disorder: An exploration of deaths in custody and fatal police contacts | QLD | Individuals with serious mental disorders who died in the context of contact with the Queensland criminal justice system. | Mixed Methods (Quantitative and Qualitative) | 38 deaths of people with serious mental disorders were identified including 2 that occurred in police custody. | To examine the common factors present across deaths of people with serious mental disorders during incarceration or following contact with police in Queensland. | Data was sourced from the Coroners Court of Queensland. Quantitative analysis was completed for descriptive variables. Coroner's reports were also analysed using qualitative thematic analysis. | The findings of the study indicated PSMDs who died during incarceration were more likely to have had psychosis or mood disorders than other forms of mental disorder, and suicide was the most common cause of death. Within these incarceration settings, access to healthcare records, medication compliance, risk assessment and monitoring, and safe housing of at-risk prisoners may be potential areas to explore regarding prevention efforts. |
| Walsh & Counter 2019 [20] | Deaths in custody in Australia: A quantitative analysis of coroners’ reports | ACT, NSW, NT, QLD, SA, TAS, VIC, WA | The study included the reports of all deaths in custody where a full-text inquest was publicly available and published between 1991 and 2016. | Content Analysis | 505 deaths in custody were analysed including 43 that occurred in police facilities or vehicles. | To report on a large-scale documentary analysis of all publicly available coroners’ reports on deaths in custody released between 1991 and 2016. | Content analysis was undertaken on each coroner’s report. Data was extracted, coded and analysed. | In the 2015 United Nations’ Universal Periodic Review, Australia was noted to have relatively ‘high mortality’ in its prisons. The concerns of the UPR Working Group focused on high rates of Indigenous incarceration, excessive use of police force and the lack of medical and mental health care in Australian custodial facilities. Our findings tend to confirm that these concerns remain unaddressed. |
| Walker et al., 2020 [21] | “They just don’t care about us!”: Police custody experiences for young men with histories of injecting drug use | VIC | Young men (aged 18-25) with histories of injecting drug use who had been in police custody in Victoria. | Qualitative Study | 28 | To expand the body of literature on the perspectives and experiences of young men in police custody. | Flexible, semi-structured qualitative interviews were completed with participants. Accounts related to experiences of police custody and their treatment while in police custody were coded into themes and analysed. | The analysis of young men’s accounts of the treatment they report to have received in PC disrupts taken-for-granted assumptions about its purpose as a place to simply detain people awaiting court and/or transfer to prison. |
| Walker et al., 2020 [22] | “That’s the Lowest Place on Earth!” Experiences of the Carceral Spaces of Australian Police Custody for Marginalized Young Men | VIC | Young men aged 19-24 with a history of injecting drug use who were held in police custody prior to transfer to adult prison. | Qualitative Study | 28 | To analyse how the harsh, degrading, and hostile architectural spaces of police custody intersected with the lived experience and lived effects of young men that experienced these environments. | Sem-structured interviews were conducted with participants and recorded. Transcripts were coded into themes and analysed. | Our study has revealed how institutional places are layered with meanings, and how architectural design and geographical space convey meanings to their occupants—communicating who the space is for and what it is intended to do. Bacchi’s framework has facilitated the destabilizing of taken-for-granted assumptions about the purpose of police custody, illustrating how the nature of carceral space has an important role to play in understanding the intentions of carceral systems. |
| Payne & Langfield 2020 [23] | How risky are heroin markets? A multi-site study of self-reported risk perceptions among police detainees in Australia | NSW, QLD, VIC, SA, WA | Individuals in police custody in one of eight DUMA program data collection sites between 2000 and 2009 who lived within 10km of the police centre in which they were detained and who self-reported either heroin use or purchasing heroin in the last 30 days. | Retrospective analysis of routinely collected data | 2257 | To assess the perception of risk in heroin markets, over time, and by different categories (age, gender, race) of police detainees. | Retrospective quantitative analysis of a subset of DUMA program data. Multilevel logistic regression was completed to model the geographical and temporal heterogeneity of risk. | A little over half of all heroin market participants consider transacting in their local market to be somewhat or very risky —the others consider it not at all or only a little risky. |
| Payne & Langfield 2020 [24] | When two measures of drug dependency do not accord: Prevalence, correlates, and implications for treatment in the criminal justice context | Multiple states or territories – Specific jurisdiction not stated | Police detainees at DUMA data collection sites between 2004 and 2010 that completed the UNCOPE screening tool with researchers and who self-reported the use of at least one illicit drug in the 30 days prior to arrest. | Retrospective analysis of routinely collected data | 13818 | To explore self-reported dependency and its concordance with the outcomes of a clinical screening tool designed to indicate DSM-V drug dependence in criminal justice populations. | Researchers analysed data from DUMA data collection sites where both the usual questionnaire and urinalysis was conducted alongside the UNCOPE screening tool. They used univariate and bivariate analyses to identify concordance. | We find that not all offenders who self- report their dependency are clinically assessed as dependent. Similarly, not all those who are clinically assessed as dependent identify as such. |
| Langfield & Payne 2021 [25] | What Factors Predict the Self Identification of Drug Dependency Among Australian Police Detainees? Prevalence, Correlates, and Implications for the Criminal Justice System | Not Stated | Police detainees interviewed at one of the DUMA program data collection sites between 2004 and 2010. who reported using at least one type of drug in the last 30 days. | Retrospective analysis of routinely collected data | 14475 | To explore the prevalence with which recent drug-using police detainees self-identify as drug-dependent and, using logistic regression, model self-identification as a function of one’s demographic, and drug-use profile. | A retrospective quantitative analysis was completed on a subset of DUMA program data from between 2004 to 2010. | We find that being female and younger is associated with an increased odds of self-identifying oneself as dependent, controlling for drug use variables. Of the five drug types, primary heroin users are the most likely to self-identify, whereas cocaine, cannabis, and amphetamine users are less likely. |
| Langfiled & Payne 2021 [26] | ‘I am drug dependent’: a study of self-identification and prior criminal justice contact using archival data from the Drug Use Monitoring in Australia (DUMA) program | Not Stated | Police detainees interviewed at one of the DUMA program data collection sites between 2004 and 2010, who reported using at least one type of drug in the last 30 days. | Retrospective analysis of routinely collected data | 14475 | To explore the correlation between criminal justice contact and the self-identification of a drug dependency among a sample of recent drug-using police detainees. | A retrospective quantitative analysis of DUMA program data was completed to explore the relationship between self-identification as being drug dependent and socio-demographic variables using logistic regression. | We find, holding constant a detainee’s frequency, longevity and type of drug use, that contact with the criminal justice system is statistically associated with higher odds of self-identifying as dependent. Further, we found those detainees who have more frequent criminal justice contact more likely to report themselves as dependent. This finding contributes to ongoing research into the complexity of identity and the management and engagement of drug-using offenders in treatment programs offered throughout the criminal justice system. |
| Hine et al., 2021 [27] | When Suspects Resist Arrest: Prevalence, Correlates, and Implications for Front-Line Policing | NSW, NT, QLD, SA, VIC, WA | Adult detainees interviewed at one of 11 DUMA program data collection sites between 2001 and 2012. | Retrospective analysis of routinely collected data | 45567 | To examine the predictors associated with suspect resistance. | Data from the DUMA program was analysed using multivariate and multi-level logistic regression to identify factors that predict suspect resistance in terms of whether the suspect was charged with resisting arrest or not | Results showed that while suspect resistance was relatively rare, it was more common under specific situations. Factors relating to offender demographics, crime, temporal/situational, and policing district all contributed to whether suspects were charged with resisting arrest. Moreover, the results showed that the policing region was the strongest predictor of whether a detainee was charged with suspect resistance. |
| Walsh 2022 [28] | Women who die in custody: What Australian coroners’ reports tell us | Multiple States or Territories - Not Stated | Individuals who died in custody and whose coroner's reports were published between 1991 and 2020 and made publicly available online. | Mixed Methods (Quantitative and Qualitative) | 736 deaths are reported but in-depth qualitative analysis is undertaken for only 34, which included 7 deaths in police cells. | The aim of this research was to investigate the nature and circumstances of Australian women's deaths in custody. | A mixed methods approach was undertaken. Descriptive statistics were presented for all the 736 coroner's reports reviewed and a detailed qualitative content analysis was conducted on the reports of detainees that identified as female. | Australian women who die in custody experience all of the individual risk factors that are associated with women’s criminalisation and incarceration– mental illness, sexual abuse, domestic violence, drug and alcohol use – and in many cases, these individual risk factors contribute directly to women’s deaths. |
| Chidgey et al., 2022 [29] | Suicide deaths following police contact: A review of coronial inquest findings | Multiple States or Territories - Not Stated | Individuals that died by suicide, and had a published coronial report indicating that they had contact with police within 48 hours of their death | Retrospective analysis of routinely collected data | 149 deaths met inclusion criteria, of which 8 occurred in police custody | To fill a gap in the literature by examining coronial inquest findings from 2008 to 2018, to investigate whether police face any barriers and enablers when responding to people who are suicidal. | Jurisdictional coroner files were searched for potential cases where individuals died from suicide within 2 days of contact with police. A qualitative document analysis of included coroners case files was completed. | We identified four themes within the recommendations, which are broadly consistent with previous studies regarding the police response to people experiencing suicidal crisis. Despite dramatic improvements in investments to prevent suicide globally, suicide deaths are increasing. |
| van de Ven et al., 2022 [30] | Australian police detainees who use anabolic-androgenic steroids (AAS) and their involvement in violent crimes compared to detainees using substances other than AAS. | NSW, QLD, SA, WA | Individuals in police custody in one of five DUMA program data collection sites between 2017 and 2021. | Retrospective analysis of routinely collected data | 10105 | To compare police detainees who at the time of arrest report the use of anabolic-androgenic steroids with those who report using other substances in the past 12 months. | A retrospective quantitative analysis of DUMA program data was completed comparing the rates of violent crime among users of anabolic-androgenic steroids to users of other substances. | Committing violent offences does not seem to be a unique feature of individuals using anabolic-androgenic steroids, with the prevalence of violent crime among detainees no different to those who use other substances. |
| Gately et al., 2024 [31] | Complex lives and procedural barriers: Detainees’ “life happens” explanations for breaching orders | WA | Police detainees at the Perth Police Watch House that were detained due to breaching an order in the last 12 months. | Mixed Methods (Quantitative and Qualitative) | 230 | To explore the explanations that detainees provide when asked about the reasons for breaching order conditions. | Quantitative analysis of data collected through the DUMA program, and for detainees in breach of an order, further qualitative survey questions were administered. | The experiences of the detainees surveyed in this study highlight the difficulties of people in contact with the justice system. Of more importance, however, is the detainees’ descriptions of how their complicated lives impact on their ability to adhere to conditions issued to them. |

Abbreviations: AAS: Anabolic-androgenic steroids. ACT: Australian Capital Territory. BJMHS: Brief Jail Mental Health Screen. CIDI-Auto: Composite International Diagnostic Interview. CJS: Criminal Justice System. DSM-IV: The Diagnostic and Statistical Manual of Mental Disorders, Fourth Edition. DSM-IV-TR: The Diagnostic and Statistical Manual of Mental Disorders, fourth edition, text revision. DUMA: Drug Use Monitoring in Australia. I-ADAM: International Arrestee Drug Abuse Monitoring. JSAT: Jail Screening Assessment Tool. NT: Northern Territory. NSW: New South Wales. PC: Police custody. PSMDs: People with serious mental disorders. QLD: Queensland. RCIADIC: Royal Commission into Aboriginal Deaths in Custody. SA: South Australia. TAS: Tasmania. VIC: Victoria. WA: Western Australia.

## **Table 2: Grey Literature**

| **Study** | **Title** | **Jurisdiction** | **Population** | **Study Design** | **Sample Size** | **Aim** | **Methods** | **Conclusions** |
| --- | --- | --- | --- | --- | --- | --- | --- | --- |
| Dalton 2000 [32] | Australian Deaths in Custody and Custody-related Police Operations 1999 | NSW, VIC, QLD, WA, SA, NT | Individuals who have died while in custody (police or prison), or who have died while police or custodial officers attempted to detain them in the year 1999. | Government Agency Surveillance Report | 85 deaths total, 26 deaths in police custody, including 6 that died in police facilities and 20 that died in police operations. | To report on the deaths in custody that occurred in Australia in 1999. | A summary report of data collected by the NDICP, at the Australian Institute of Criminology for the year 1999. | N/A (only has a few conclusions on prison deaths). |
| Makkai et al., 2000 [33] | Patterns of Drug Use Amongst Police Detainees: 1999–2000 | NSW, QLD, WA | Individuals in police custody in one of four testing sites spread over Sydney, Gold Coast, and Perth. | Government Agency Report | 1759 | To provide interim findings from the Drug Use Monitoring Australia pilot up to the middle of 2000. | Face to face interviews and urine specimen analysis. | The authors observed stable patterns of cannabis and opiate use in police detainees over an 18-month period. Results varied by site indicating that local strategy and policy is vital. |
| Makkai et al., 2000 [34] | Drug Use Among Police Detainees | NSW | Police detainees in one of the two DUMA data collection sites in NSW, Australia. | Government Agency Report | 379 | To examine the level and type of drug use amongst a sample of detainees from two Local Area Commands in Sydney. | A combination of a self-report survey detailing a range of criminal justice, demographic, drug use and drug market participation information and voluntary urinalysis. | The DUMA results show illicit drug use to be widespread among detainees. Of the persons who provided a urine sample for the study, 75.1 per cent tested positive to a least one drug. Cannabis and opiates were the most commonly used substances. |
| Makkai 2000 [35] | Drug Use Monitoring in Australia (DUMA): 1999 Annual Report on Drug Use Among Adult Detainees | NSW, QLD, WA | Individuals in police custody at one of four DUMA program data collection sites in 1999. | Government Agency Surveillance Report | 1402 | To describe the key results from the DUMA program data collected during 1999. | A combination of a self-report survey detailing a range of criminal justice, demographic, drug use and drug market participation information and voluntary urinalysis. | N/A |
| Makkai 2000 [36] | Drug transactions: Some results from the DUMA project | NSW, QLD, WA | Adult male detainees at one of the four initial DUMA data collection sites in 1999. | Government Agency Report | Not Reported | To provide an assessment of the extent and nature of the illicit drug situation in Australia for 1998–99, as well as a synopsis of the primary illicit drug–cultivating and–production countries. | Data was collected through questionnaires, but other sources such as international drug reports, research documents, interviews and letters of request were also used to populate the report. | The data shows that drug markets vary according to site and drug. |
| Loxley & Lien 2001 [37] | Drug Use Monitoring in Australia: Western Australian 2000 Report on Drug Use Among Police Detainees | WA | Adults in police custody at the East Perth Lockup in 2000. | Government Agency Surveillance Report | 570 | To describe the key results from the DUMA data collected from the East Perth site during 2000. | A combination of a self-report survey detailing a range of criminal justice, demographic, drug use and drug market participation information and voluntary urinalysis. | Testing revealed that 60% of all respondents in 2000 were positive for cannabis; around 40% were positive for amphetamines and around 25% were positive for opiates and benzodiazepines. There were very few positive cases of cocaine, and around 5% were positive for methadone. The majority of respondents were male, and almost half were aged between 21 and 30. One in four defined themselves as Indigenous Australians. The offences for which they were charged were most likely to be property offences, with violent and traffic offences being the second and third most prevalent categories. |
| Collins & Mouzos 2001 [38] | Australian Deaths in Custody and Custody-related Police Operations, 2000 | NSW, VIC, QLD, WA, SA | Individuals who have died while in custody (police or prison), or who have died while police or custodial officers attempted to detain them in 2000. | Government Agency Surveillance Report | 91 deaths in custody with 25 occurring in police custody. | To provide the latest information on deaths in custody in Australia for the year 2000. | Data collected by the National Deaths in Custody Monitoring and Research Program for the year 2000 is summarised and presented in tables and figures. | N/A |
| Williams 2001 [39] | Deaths in Custody: 10 Years on from the Royal Commission | NSW, VIC, QLD, SA, WA, TAS, NT, ACT, External Australian Territory | Individuals who have died while in custody (police or prison), or who have died while police or custodial officers attempted to detain them from 1990-1999. | Government Agency Surveillance Report | 78 non-Indigenous deaths and 21 Indigenous deaths in police custody between 1990 -1999. | To examine the number and circumstances of deaths in custody for the decade examined by the Commission are compared with those which occurred in the decade since. | Analysis of case records collected by the NDICP. | Even with the addition of another category of deaths (police operations) in the decade since the Royal Commission, the average annual rate of death of Indigenous persons in custody, relative to population size, diminished slightly (from 4.4 per 100,000 to 3.8 per 100,000 persons). |
| Makkai & McGregor 2001 [40] | Drug Use Monitoring in Australia (DUMA): 2000 Annual Report on Drug Use Among Police Detainees | NSW, QLD, WA | Individuals in police custody in one of the four DUMA data collection sites in 2000. | Government Agency Surveillance Report | 2121 | To provide illicit drug use information on those people who are detained and brought to a police station. | A combination of a self-report survey detailing a range of criminal justice, demographic, drug use and drug market participation information and voluntary urinalysis. | N/A |
| Ombudsman Victoria 2002 [41] | Report on Conditions and Overcrowding in Police Cells | VIC | Individuals who have been sentenced, or refused bail, but who have not been received into the custody of the Correctional Services Commissioner. Also, any prisoner held in police custody. | Government Investigation or Inquiry | N/A | To record the results of a review into the effectiveness of action taken to date by the various authorities which have responsibility for prisoners in Victoria. | Interviews with staff and inspection of custodial facilities. | This investigation makes 10 recommendations including recommendations for improving health services, sanitation, access to fresh air, and the segregation of prisoners. |
| Freeman & Fitzgerald 2002 [42] | Drug use monitoring of police detainees in New South Wales: The first two years | NSW | Police detainees in Parramatta and Bankstown police stations from June 1999 to July 2001. | Government Agency Report | 1695 | The purpose of this bulletin is to report on some of the key findings that have emerged from the data collected at the two NSW DUMA sites throughout the project’s first two years of operation in NSW (from June 1999 to July 2001). | A combination of a self-report survey detailing a range of criminal justice, demographic, drug use and drug market participation information and voluntary urinalysis. | The DUMA results continue to show a high prevalence of illicit drug use among police detainees, with over 70 per cent of the sample indicating illicit drug use in the past 12 months and half of the sample indicating recent use of at least one illicit drug. |
| Poyser et al., 2002 [43] | Drug Driving Among Police Detainees in Three States of Australia: Final Report | NSW, QLD, WA | Individuals in police custody in one of three DUMA program data collection sites between 1999 and 2001. | Government Agency Report | 2525 | To utilise DUMA program data to describe the extent of drug driving amongst detainees, the patterns of drug use of these detainees, their socio-demographic profiles, and to provide empirical evidence for police deliberations. | A retrospective quantitative analysis of DUMA program data from 1999 to 2001 specifically focussing on detainees with traffic offenses. | In brief, it is a significant road safety concern that the majority of DUMA traffic detainees tested positive to psychoactive drugs other than alcohol. It should be noted that the data does not allow us to determine whether the traffic detainees were actually impaired while driving. It does appear, however, that taking drugs and driving is an acceptable practice amongst this sample. |
| Weierter & Lynch 2002 [44] | Drug use and crime: Findings from the DUMA survey | NSW, QLD, WA | Individuals in police custody at one of four DUMA program data collection sites between 1999 and 2001. | Government Agency Report | 5440 | To explore the relationship between drug dependency and crime, and between drug dependency and such factors as age, sex, schooling and drugtaking. | A retrospective quantitative analysis of DUMA data from a three-year period. | There are two areas of concern highlighted in the data. Firstly, there is a greater proportion of females testing positive for opiates compared to males suggesting a stronger than expected heroin-crime relationship. Secondly, polydrug use is a concern in the detainee population. |
| Collins & Mouzos 2002 [45] | Deaths in Custody: A Gender-specific Analysis | Multiple States or Territories - Not Stated | Females who have died in custody in Australia between 1980 and 2000. | Government Agency Report | 75 deaths with 38 occurring in police custody | To examine female deaths in all forms of custody, with specific focus on the circumstances and characteristics of the 75 female deaths in custody that have occurred in Australia between 1980 and 2000. | Retrospective analysis of data from the National Deaths in Custody Program | This study has identified the existence of gender differentials among persons who died in custody in Australia. Most importantly, the study has found that women do not die in the same circumstances as their male counterparts. |
| Collins 2002 [46] | Deaths in Custody in Australia: 2001 National Deaths in Custody Program (NDICP) Annual Report | NSW, VIC, QLD, SA, WA | Individuals who died while in custody (police, or prison detention), or who died while police or custodial officers attempted to detain them in 2001. | Government Agency Surveillance Report | 31 deaths in police custody including 7 category 1 deaths, and 2 deaths that occurred in police cells. | To present detailed tabulated information on deaths in custody in Australia for the year 2001 and to provide jurisdictional breakdowns for comparative purposes. | Data collected by the NDICP for the year 2001 are summarised and presented in tables and figures. | N/A |
| Makkai & McGregor 2002 [47] | Drug Use Monitoring in Australia (DUMA): 2001 Annual Report on Drug Use Among Police Detainees | NSW, QLD, WA | Individuals in police custody in one of the four DUMA data collection sites in 2001. | Government Agency Surveillance Report | 111 juveniles & 1808 adults | To provide illicit drug use information on those people who are detained and brought to a police station. | A combination of a self-report survey detailing a range of criminal justice, demographic, drug use and drug market participation information and voluntary urinalysis. | N/A |
| McGregor & Makkai 2003 [48] | Self-reported Drug Use: How Prevalent is Under-reporting? | NSW, QLD, SA, WA | Police detainees in one of the seven DUMA data collection sites between 1999 and 2002. | Government Agency Report | 6477 | To examine self-reported drug use among a sample of police detainees and compares that information with results of urinalysis testing | Results from DUMA self-report surveys were compared with results from urinalysis. | While detainees who use illicit drugs and have been in contact with the criminal justice system or in treatment are more likely to report accurately, others who lead more socially accepted lifestyles are more likely to under-report their drug use. |
| Collins & Ali 2003 [49] | Deaths in Custody in Australia: 2002 National Deaths in Custody Program (NDICP) Annual Report | NSW, QLD, WA, NT, VIC, TAS | Individuals who died while in custody (police, or prison detention), or who died while police or custodial officers attempted to detain them in 2002. | Government Agency Surveillance Report | 19 deaths in police custody including 5 category 1 deaths. No deaths occurred in police cells. | To present detailed tabulated information on deaths in custody in Australia for the year 2002 and to provide jurisdictional breakdowns for comparative purposes. | Data collected by the NDICP for the year 2002 are summarised and presented in tables and figures. | N/A |
| Wei et al., 2003 [50] | Drug Use Among a Sample of Juvenile Detainees | NSW, QLD | Juveniles in police custody in one of the NSW or QLD data collection sites in DUMA from 1999 to 2002. | Government Agency Report | 493 | To analyse some patterns of drug use, age at first drug use, the association between drug use and offending, and other characteristics of juvenile arrestees. | An analysis of data previously collected through the DUMA program between 1999 and 2002. | This paper has demonstrated that drug use among juvenile detainees is prevalent and that reported access to treatment is low. The importance of the juvenile justice system as a point of referral to drug treatment cannot be underestimated. |
| Makkai & McGregor 2003 [51] | Drug Use Monitoring in Australia (DUMA): 2002 Annual Report on Drug Use Among Police Detainees | NSW, QLD, SA, WA | Individuals in police custody in one of the four DUMA data collection sites in 2002. | Government Agency Surveillance Report | 3634 | To provide illicit drug use information on those people who are detained and brought to a police station. | A combination of a self-report survey detailing a range of criminal justice, demographic, drug use and drug market participation information and voluntary urinalysis. | N/A |
| Milner et al., 2004 [52] | Drug Use Monitoring in Australia: 2003 Annual Report on Drug Use Among Police Detainees | NSW, QLD, SA, WA, | Individuals in police custody at one of seven DUMA program data collection sites in 2003. | Government Agency Surveillance Report | 3705 | To describe the key results from the DUMA program data collected during 2003. | A combination of a self-report survey detailing a range of criminal justice, demographic, drug use and drug market participation information and voluntary urinalysis. | N/A |
| McCall 2004 [53] | Deaths in Custody in Australia: 2003 National Deaths in Custody Program (NDICP) Annual Report | NSW, VIC, QLD, WA, SA, NT | Individuals who died while in custody (police, or prison detention), or who died while police or custodial officers attempted to detain them in 2003. | Government Agency Surveillance Report | 29 deaths in police custody and custody-related police operations including 10 category 1 deaths, and 3 that occurred in police cells. | To provide the latest information on deaths in custody in Australia for the year 2003. | Data collected by the National Deaths in Custody Monitoring and Research Program for the year 2003 are summarised and presented in tables and figures. | In comparison to 2002, the total number of custodial deaths was similar. The number of deaths in police custody decreased from 33 in 2002 to 29 in 2003. Deaths in prison custody also declined from 50 deaths in 2002 to 39 deaths in 2003. In particular, Victoria recorded the lowest number of prison custody deaths (n=1) since the NDICP began recording deaths in custody in 1980. |
| Milner & McGregor 2004 [54] | Cocaine Use Among a Sample of Police Detainees | NSW, QLD, SA, WA | Police detainees in one of the seven DUMA data collection sites between 1999 and 2003. | Government Agency Report | 9510 | To examine the use of cocaine among a group of individuals detained by police and interviewed for the AIC’s DUMA program. | An analysis of data previously collected through the DUMA program between 1999 and 2003. | The analysis shows that while most cocaine use occurs in DUMA’s Sydney sites, use also occurs in other areas. Furthermore, cocaine users among this cohort are predominately multiple-drug users. |
| Sallybanks 2005 [55] | Monitoring Injuries in Police Custody: A Feasibility and Utility Study | NSW | Individuals injured in police custody in NSW between November 2001 and June  2003. | Government Agency Report | 260 | The main objective of the pilot study was to assess an injury monitoring system in police custody in NSW. The assessment aimed to 1) measure the nature and extent of injuries occurring during the custody process; 2) monitor service standards and duty of care; 3) deliver trend data; 4) inform evaluation activities in relation to the implementation of injury-minimisation policies and practices; and 5) inform police-related injury research in general and situational analyses more particularly | A retrospective analysis of data from the NSW computerised operational policing system was completed to identify individuals injured in police custody during the study period, and to collect information on injured detainee demographics and the circumstances of the injury. | The monitoring system is invaluable for extending our understanding of injuries occurring in custody. Such a system is a very useful management tool to assist in developing strategies regarding safe custody practice. |
| Australian Capital Territory Ombudsman 2005 [56] | Australian Capital Territory Ombudsman Annual Report 2004-2005 | ACT | This report includes two case studies: one of a woman, and another of a young male, held in the ACT City Watch House. | Government Agency Report | 2 | To comply with annual reporting requirements and report on major issues, challenges and achievements of the ACT Ombudsman in 2004-2005. | Qualitative descriptions and summaries of some cases are provided in the annual report. | N/A |
| Taylor & Bareja 2005 [57] | 2002 National Police Custody Survey | NSW, QLD, WA, SA, VIC, NT, ACT, TAS | Anyone taken into police custody in Australia and lodged in a police cell in the month of October 2002. | Government Agency Surveillance Report | 27047 | To presents findings from the fourth National Police Custody Survey which was conducted in October 2002. The document aims to obtain information on the extent and nature of police custody incidents over a one-month period in order to identify flows into and out of police custody, who goes into custody and why, and to provide comparisons in custody over time. | The report used data from two sources. Data from ACT, VIC, and SA were collected by police officers at the station level on hard copy forms and forwarded to the AIC for coding and data entry. Data from NSW, QLD, WA, NT and TAS were provided to the AIC in the form of electronic datasets. The data included detainee demographics, offense details, and duration in custody. | It appears that there has been some progress toward meeting key recommendations of the RCIADIC. This is reflected primarily in reduced rates of Indigenous custody in some jurisdictions, as well as a reduction in the percentage of custody incidents attributable to public drunkenness. |
| Putt et al., 2005 [58] | Indigenous Male Offending and Substance Abuse | NSW, QLD, SA, WA (police detainee data) | Adult male prisoners surveyed through the DUCO project in 2001, and adult police detainees surveyed through the DUMA program in 2002 and 2003. | Government Agency Report | 5797 male police detainees and 2135 male prisoners | To compare Indigenous and non-Indigenous male offenders’ drug use and offending in order to identify how better to prevent and respond to drug-related crime | A retrospective quantitative analysis of DUMA and DUCO data was completed. | The results indicate that Indigenous male offenders are more likely to have earlier and more serious contact with the criminal justice system. It also seems that urban Indigenous male offenders have similar experiences with illicit drugs to their non-Indigenous counterparts, including intravenous use, dependency and involvement in the illicit drug trade. |
| Schulte et al., 2005 [59] | Drug Use Monitoring in Australia: 2004 Annual Report on Drug Use Among Police Detainees | NSW, QLD, SA, WA | Individuals in police custody at one of seven DUMA program data collection sites in 2004. | Government Agency Surveillance Report | 3834 | To describe the key results from the DUMA program data collected during 2004. | A combination of a self-report survey detailing a range of criminal justice, demographic, drug use and drug market participation information and voluntary urinalysis. | N/A |
| Joudo & Veld 2005 [60] | Deaths in Custody in Australia: National Deaths in Custody Program Annual Report 2004 | NSW, QLD, VIC, WA, SA, NT, ACT | Individuals who died while in custody (police or prison), or who died while police or custodial officers attempted to detain them in 2004 | Government Agency Surveillance Report | 28 deaths occurred in police custody and custody-related operations, of which 9 were category 1 deaths and 1 death occurred in a police cell. | To provide the latest information on deaths in custody in Australia for the year 2004. | Data collected by the NDICP for 2004 is summarised and presented in tables and figures. | N/A - the 2004 report did not include a conclusion. |
| Office of the Inspector of Custodial Services 2005 [61] | Directed Review of the Management of Offenders in Custody | WA | N/A | Government Investigation or Inquiry | N/A | To inquire on the practices of the Department of Justice, the placement and transport of prisoners, the identification of infrastructure needs and prioritization requirements for the next decade, and likely staffing needs. | Consultation, community correction and prison visits, interviews, document reviews, and the review of submissions. | The report makes 162 recommendations to the Minister of Justice. |
| Ziersch & Turner 2005 [62] | Drug Use and Vehicle Crime - An Analysis of DUMA Data on Offenders Arrested for Motor Vehicle Theft | NSW, QLD, SA, WA | Police detainees at one of seven DUMA data collection sites between 2003 and 2004. | Government Agency Report | 7263 | To provide a summary of the characteristics of detainees apprehended for a motor vehicle theft offence and compare these characteristics to detainees apprehended for other offenses. | A retrospective quantitative analysis of DUMA program data with a specific focus on those apprehended for motor vehicle theft. | The findings of this study indicate that motor vehicle theft offenders are more likely to have earlier and more entrenched contact with the criminal justice system. These offenders recorded higher rates of imprisonment and were more likely to earn income from illegal activity. |
| Ombudsman Victoria & Office of Police Integrity Victoria 2006 [63] | Conditions for persons in custody: Report of Ombudsman Victoria and Office of Police Integrity | VIC | N/A | Government Investigation or Inquiry | N/A | To report on, and to clarify the conditions in both prisons and police cells at various stages of the custody process. | This Ombudsman investigation included reviewing documents, facility inspections, discussions with police and prison staff, and discussions with detainees. | Police are not jailers and do not see themselves as such. The lack of police training in this role, coupled with the inadequate physical conditions and design of police watch-houses makes it inappropriate to hold detainees for longer than 48 hours. Monitoring the length of stays of detainees in police cells over the last six months has shown that many detainees, including those who are vulnerable and/or have ‘protection’ status, are held in unsuitable conditions for long periods. |
| Mouzos et al., 2006 [64] | Drug use monitoring in Australia: 2005 annual report on drug use among police detainees | NSW, QLD, SA, WA | Individuals in police custody in one of the seven DUMA data collection sites in 2005. | Government Agency Surveillance Report | 3786 | To describe the key results from the DUMA data collected during 2005. | A combination of a self-report survey detailing a range of criminal justice, demographic, drug use and drug market participation information and voluntary urinalysis. | N/A |
| Joudo 2006 [65] | Deaths in custody in Australia: National Deaths in Custody Program annual report 2005 | NSW, VIC, QLD, WA, SA | Individuals who died while in custody (police or prison), or who died while police or custodial officers attempted to detain them in 2005 | Government Agency Surveillance Report | 20 deaths occurred in police custody and custody-related operations, of which 5 were category 1 deaths and 1 death occurred in a police cell. | To provide the latest information on deaths in custody in Australia for the year 2005. | Data collected by the NDICP for the year 2005 is summarised and presented in tables and figures. | N/A - the 2005 report did not include a conclusion. |
| Joudo 2006 [66] | Deaths in custody in Australia 1990–2004 | Multiple States or Territories – Not Stated | Individuals who have died while in custody (police or prison), or who have died while police or custodial officers attempted to detain them from 1990-2004. | Government Agency Surveillance Report | 452 deaths in police custody and police operations. 219 occurred in police custody (category 1a or 1b) and 233 occurred in police operations. | To examine the trends in deaths in custody over the 15-year period following the RCIADIC, from 1990 to 2004. | A summary report of data collected by the NDICP for the years 1990 to 2004. | Although the majority of Australian deaths in custody occurred in police custody between 1980 and 1989, the trend in the 15 years since the RCIADIC reveals a different picture. Prison custody deaths account for the majority of deaths in custody since 1990 and have exceeded all other deaths each year. Furthermore, while deaths in police custody have decreased since 1990, deaths in police operations have been increasing. |
| Australian Federal Police & the Commonwealth Ombudsman 2007 [67] | Review of ACT Policing’s Watchhouse operations: Joint report by the Australian Federal Police and the Commonwealth Ombudsman | ACT | N/A | Government Investigation or Inquiry | N/A | To examine the policies, practices, and procedures applying to watchhouse operations and to make recommendations on any actions required to improve all aspects of watchhouse operations. | The policies and procedures relevant to watchhouse operations, and staff training were reviewed. A review was conducted on the use of force and restraints, and the treatment of people with special needs. A sample of watchhouse records and videotapes were reviewed. Facilities and amenities were assessed, and interviews were also undertaken. | The Watchhouse facilities are in good condition and largely compliant with the 1991 recommendations of the  RCIADIC. However, the review identified deficiencies in many aspects of Watchhouse operations. |
| Ombudsman Victoria 2007 [68] | Investigation into the use of excessive force at the Melbourne Custody Centre | VIC | N/A | Government Investigation or Inquiry | N/A | To investigate the complaints of a detainee of the Melbourne Custody Centre, that alleged officers used excessive force against him. | A review of CCTV footage, interviews with custodial and medical officers from MCC, Victoria Police, and site management. | My investigation found that excessive force was used on Mr A by MCC officers which I considered was unwarranted in the circumstances. I am satisfied that MCC Officer X overreacted in the incident and used excessive force upon Mr A and that fellow Officer Z struck him to the head. |
| Mouzos & Smith 2007 [69] | Partner violence among a sample of police detainees | Multiple States or Territories - Not Stated. | Adults in police detention at one of the seven DUMA data collection sites where the addendum questions on violence in the home were administered. | Government Agency Report | 1597 | To examine intimate partner violence among a sample of police detainees. Associations with their drug use and other offending behaviour are also examined. | Administration of additional survey questions on family violence during the routine DUMA data collection. | Female detainees generally experienced more partner violence in their lifetime than males. However, it was also found that female detainees were more likely to be perpetrators of partner violence than male detainees. |
| Mouzos et al., 2007 [70] | Drug use monitoring in Australia: 2006 annual report on drug use among police detainees | NSW, NT, QLD, SA, VIC, WA | Police detainees in one of the nine DUMA data collection sites in 2006. | Government Agency Surveillance Report | 4555 | To describe the key results from the DUMA data collected throughout 2006. | A combination of a self-report survey detailing a range of criminal justice, demographic, drug use and drug market participation information and voluntary urinalysis. | N/A |
| Loxley 2007 [71] | Benzodiazepine use and harms among police detainees in Australia | NSW QLD WA | Police detainees over 18 years old that consented to participate in the DUMA surveys from 1999 to 2005. | Government Agency Report | 12835 | To investigates the prevalence of legal and illegal benzodiazepine use and harms in police detainees. | Analysis of previously collected DUMA data (1999-2005) specifically in relation to any self-reported legal or illegal use of benzodiazepines. | Almost 18% of the DUMA sample had used benzodiazepines in the previous month and 15% had used illegal benzodiazepines in the previous 12 months. The majority of illegal benzodiazepine users had also used heroin and/or amphetamines in the same time frame. |
| ACT Human Rights Commission 2007 [72] | Human Rights Audit on the operation of ACT Correctional Facilities under Corrections legislation | ACT | N/A | Government Investigation or Inquiry | 36 detainees were interviewed as part of the broader audit | To review the effect and implementation of existing and proposed Territory law governing the operations of ACT Correctional Facilities. In particular, the operation of Belconnen Remand Centre and Symonston Temporary Remand Centre will be audited. [Please note neither of these are police custody settings, however the report does include some small sections on police custody]. | A review of the legal framework, operations and practices of the two remand centres was undertaken. | This human rights audit makes 96 recommendations over seven broad categories which include changes to corrections culture, the health of detainees, systemic discrimination and the human treatment of detainees. |
| Crime and Misconduct Commission Queensland 2008 [73] | Drugs and Crime: Trends among watch-house detainees | NSW, QLD, SA, WA | Police detainees participating in the DUMA program at Queensland data collection sites between 1999 and 2005. Comparisons are also made to detainee data from NSW, WA, and SA. | Government Agency Report | 5260 Queensland detainees | To provide an accurate depiction of illegal drug use among Queensland watch-house detainees between 1999 and 2005, and comparative information (2004–05) about detainees in watch-houses in other states (NSW, WA and SA). | A retrospective analysis of DUMA program data focussing on data from Queensland sites. | This report has provided an in-depth analysis of the illicit drug-using behaviours and prior criminal histories of samples of Queensland watch-house detainees. It has also shown trends in these behaviours over time, and comparisons with other states.  Not surprisingly, the socio-demographic profile of the participating detainees indicated a predominance of single males, aged about 30 years, with low levels of education and high levels of unemployment. |
| Adams et al., 2008 [74] | Drug use monitoring in Australia: 2007 annual report on drug use among police detainees | NSW, NT, QLD, SA, VIC, WA | Police detainees in one of the ten DUMA data collection sites in 2007. | Government Agency Surveillance Report | 3911 | To describe the key results from the DUMA data collected in 2007 from the ten participating sites. | A combination of a self-report survey detailing a range of criminal justice, demographic, drug use and drug market participation information and voluntary urinalysis. | N/A |
| Adams et al., 2008 [75] | Drug driving among police detainees in Australia | NSW, QLD, SA, WA | Police detainees in selected DUMA data collection sites who had driven in the previous 12 months. | Government Agency Report | 1215 | To explore the characteristics of police detainees who drive after drug use and their perceptions of the risks in doing so. | Researchers implemented an addendum survey for DUMA participants, if they reported driving a vehicle in the previous 12 months. | This study highlights the significant issue of driving following the use of drugs and/ or alcohol in Australia. Over half of detainees who had driven a car or other vehicle in the past 12 months reported driving after they had used drugs other than alcohol and over one-third reported driving after they had used alcohol. |
| Joudo & Curnow 2008 [76] | Deaths in custody in Australia: National Deaths in Custody Program annual report 2006 | NSW, VIC, QLD, WA, SA, NT, ACT | Individuals who died while in custody (police or prison), or who died while police or custodial officers attempted to detain them in 2006 | Government Agency Surveillance Report | 22 deaths occurred in police custody and custody-related operations, of which 6 were category 1 deaths. No deaths occurred in police cell. | To provide the latest information on deaths in custody in Australia for the year 2006. | Data collected by the NDICP for the year 2006 is summarised and presented in tables and figures. | The overall number of deaths in custody has decreased during the period covered by the NDICP collection. Police custody and custody-related deaths have been declining since 2002 despite a change in definition in 1990 that expanded the remit of the program. |
| Crime and Misconduct Commission Queensland 2009 [77] | Restoring Order: Crime prevention, policing and local justice in Queensland’s Indigenous communities | QLD | N/A | Government Investigation or Inquiry | N/A | To examine issues relating to policing in Queensland’s Indigenous communities and make recommendations with respect to three terms of reference: 1. Possible changes to existing policy and procedure that would result in improved relations between the QPS and Indigenous communities. 2. Current practices relating to detention and monitoring of detainees in police custody and the possible involvement of community justice groups or other civilians in the monitoring of detainees. 3. The optimal use of state resources to deliver criminal justice services in Queensland’s Indigenous communities. | The inquiry has gathered information from five different sources: 1. Consultations with Queensland’s Indigenous communities  2. Written and oral submissions 3. A public forum held in Cairns 4. Queensland criminal justice system data, mostly provided by the QPS 5. A review of policy and research literature. | The number of deaths occurring in police watch-house cells has decreased dramatically in Queensland since the Royal Commission, but all such deaths remain tragic events that warrant the closest scrutiny. The decrease in deaths has been accompanied by improvements in watch-house facilities and improvements in the standard of care provided to detainees after the Royal Commission’s recommendations. |
| Forsythe & Adams 2009 [78] | Mental health, abuse, drug use and crime: does gender matter? | NSW, QLD, SA, WA | Police detainees 18 years and over in one of the seven long-term DUMA data collection sites between 2002-2006. | Government Agency Report | 18280 | To explore the relationship between drug use, offending, mental health and experiences of abuse among a sample of police detainees in Australia. | Addendum questions on mental health and prior child abuse were administered in addition to the usual DUMA self-report survey and urinalysis. | The results from this study provide further support for the differential patterns of male and female drug use and offending. |
| Loxley & Adams  2009 [79] | Women, drug use and crime: findings from the Drug Use Monitoring in Australia program | NSW, QLD, SA, WA | Adults in police custody at participating DUMA sites between the second quarter of 2002 and the end of 2006. | Government Agency Report | 17861 | There are three key aims. To assess the factors associated with women's drug use and/or criminal behaviour compared to men's. To assess the extent to which women in police detention are similar to women in prison. To examine the extent to which Indigenous women are similar to non-Indigenous women in DUMA. | Retrospective analysis of DUMA data from 2002 to 2006 with comparisons made between male and female police detainees, and between female detainees, the general population in Australia, and the prisoner population. | The trajectories towards drug use and/or criminal behaviour appear to be different among female police detainees in DUMA from those of men. In support of past research, female police detainees were the more likely to be using illicit drugs on a regular basis prior to their first involvement with the criminal justice system, while the opposite pattern was found for male police detainees. |
| Curnow & Joudo Larsen  2009 [80] | Deaths in custody in Australia: National Deaths in Custody Program 2007 | NSW, VIC, QLD, WA, SA, NT | Individuals who have died while in custody (police or prison), or who have died while police or custodial officers attempted to detain them in 2007. | Government Agency Surveillance Report | 74 deaths in custody with 29 occurring in police custody. | To provide the latest information on deaths in custody in Australia for the year 2007. | Data collected by the NDICP for the year 2007 is summarised and presented in tables and figures. | There has been a significant decline in the total number of deaths which have occurred in custody since 1997. Over this period, deaths declined from 105 in 1997 to 74 in 2007. |
| Office of Police Integrity, Victoria 2010 [81] | Update on conditions in Victoria Police cells | VIC | Individuals held in police cells in Victoria in 2009. | Government Investigation or Inquiry | 24,777 | To summarises the findings of an audit conducted by the Professional Standards Assurance Unit of the OPI from April to December 2009 of  category ‘A’ police cells operated by Victoria Police. | Inspections of 22 police stations were completed between April and December 2009. Facilities, systems and processes were reviewed and evaluated. | Inconsistency between cell complexes on a broad range of issues emerges as the major theme arising from the cells audit conducted by OPI. The inconsistencies can be explained in variety of ways. The availability of staff and the willingness of managers to assign them to custodial duties affects the flexibility that is incorporated into watch-house rules. The location of the complex can have a significant impact on the conditions experienced by detainees. Conditions varied according to whether cells were adjacent to a court, in the city or in the country. Quieter cells and cells in rural locations were often more flexible regarding the number of visits a detainee could have. Yet staff at busier cells generally exhibited a greater knowledge of detainee management practices. |
| Gaffney et al., 2010 [82] | Drug use monitoring in Australia: 2008 annual report on drug use among police detainees | NSW, NT, QLD, SA, VIC, WA | Police detainees in one of the nine DUMA data collection sites. | Government Agency Surveillance Report | 4107 adults and 136 juveniles | To describe the key results from the Drug Use Monitoring in Australia program in 2008 from the nine included sites. | A self-report survey detailing a range of criminal justice, demographic, drug use and drug market participation information as well as voluntary urinalysis. | No conclusion or unifying summary like those provided in more recent years is provided in this edition. |
| Lyneham et al., 2010 [83] | Deaths in custody in Australia: National Deaths in Custody Program 2008 | WA, VIC, NT, NSW, QLD, SA | Individuals who have died while in custody (police or prison), or who have died while police or custodial officers attempted to detain them in 2008. | Government Agency Surveillance Report | 86 total deaths in custody, with 32 in police custody and custody-related operations. | To report on deaths in prison custody, as well as deaths in police custody and custody-related operations across Australian states and territories for the year 2008. | Data collected by the NDICP for the year 2008 is summarised and presented in tables and figures. | The number of deaths in police custody and custody-related operations remained stable between 1990 and 2000, peaked in 2002 and then declined each year to 2006. In 2008, there was a marginal increase from the 2007 figure, but overall, the number of deaths remains in line with recorded levels in recent years. |
| Office of the Inspector of Custodial Services 2010 [84] | Thematic review of court security and custodial services in Western Australia | WA | N/A | Government Investigation or Inquiry | N/A | To review services generally conducted under the Court Security and Custodial Services Act 1999, inclusive of those provided both by the public and private sectors. | The review included custodial facility and court custody centre inspections, interviews, and the review of submissions. | Almost a decade after the CSCS Contract the police lockup system remains unreformed, with police largely unrelieved of associated custodial duties. Reform of the system of management of police lockups in Western Australia, in parallel with deliberations towards the CSCS Contract re-tender, is urgently required. |
| Gately et al., 2011 [85] | Amphetamine use among detainees at the East Perth Watch House: What is the impact on crime? | WA | Police detainees in the East Perth Watch House from 1999 to 2009. | Government Agency Report | 6993 | There were three primary objectives. 1) To Identify and analyse the relationships between amphetamine use indicators and specific crime classifications taking account of potential confounders such as age, gender and other drug use. 2) Develop predictor variables for the links between amphetamine use indicators and specific crime classifications. 3) To identify and analyse co-occurring changes in amphetamine use indicators and trends in reported crime in WA over the six-year period between 2002 and 2008 | Data was taken from DUMA, from the WA Police monthly drug seizure data, and from the WA monthly reported crime data. Logistic regression was completed to predict whether amphetamine use was associated with specific types of crimes. | This study revealed the relationships between important indicators such as drug use, crime and interventions that can be used to provide support for the provision or cessation of specific intervention activities. |
| McGregor et al., 2011 [86] | Prescription drug use among detainees: Prevalence, sources and  links to crime | NSW, NT, QLD, SA, VIC, WA | Police detainees in one of the eight DUMA data collection sites that administered the addendum survey on prescription drug use. | Government Agency Report | 986 | To assess the prevalence of non-medical prescription drug use among police detainees and the differences between users and non-users of prescription drugs. | A self-report survey detailing a range of criminal justice, demographic, drug use and drug market participation information as well as voluntary urinalysis. | The prevalence of non-medical prescription drug use in the previous 12 months (19%) was markedly higher among police detainees in comparison to the general community. Prescription drug use was higher in women, younger people, the unemployed and detainees who considered themselves drug dependent. |
| Sweeney & Payne 2011 [87] | Poly drug use among police detainees | NSW, NT, QLD, SA, VIC, WA | Adults in police detention in one of the nine data collection sites for the Drug Use Monitoring in Australia program. | Government Agency Report | 3852 | To assess the number of poly drug users in police detention, the types of drugs they use and how these patterns differ over time. | Analysis of data collected in 2009 through the Drug Use Monitoring Australia program. | The prevalence of poly drug use varies depending on how it is defined and calculated. It is estimated that 44 percent of detainees had used two or more different drug types in the 12 months prior to their arrest; 30 percent had used two or more drug types in the past 30 days and 12 percent were recent poly drug users, having used two or more different drug types in the 48 hours before their arrest. |
| Ness & Payne 2011 [88] | Patterns of mephedrone, GHB, Ketamine and Rohypnol use among police detainees: Findings from the DUMA program | NSW, NT, QLD, SA, VIC, WA | Police detainees in one of the eight DUMA data collection sites in the third quarter of 2010. | Government Agency Report | 824 | To report on the results of an addendum survey completed by DUMA on emerging or less common drug types. | Addendum questions on mephedrone, Rohypnol, GHB and Ketamine were administered in addition to the usual DUMA self-report survey and urinalysis. | This study is the first to use a large sample of Australian police detainees to investigate both the knowledge and prevalence of use for newly emerging and less commonly used drugs such as mephedrone, GHB, Ketamine and Rohypnol. While the findings indicate a relatively low level of use, there nevertheless remains a need for ongoing assessment to identify changing trends and patterns of use that can be responded to accordingly |
| The Parliament of the Commonwealth of Australia 2011 [89] | Doing Time - Time for Doing: Indigenous youth in the criminal justice system | NSW, WA | N/A | Government Investigation or Inquiry | N/A | To inquire into the high levels of involvement of Indigenous juveniles and young adults in the criminal justice system. | A review of submissions from Commonwealth, state and territory government departments, police organizations, non-profit organizations, and Indigenous rights' advocacy groups. In addition, 18 hearings were held, and visits to youth detention sites were completed. | From Mr Shayne Neumann MP, "Too many Indigenous juveniles and youth have done time in our detention centres and prisons. Now it is time to do something about it. It is my hope that this report, Doing Time – Time for Doing, will assist in bringing about positive change for Indigenous Australians and that the future will be one of strength and partnership between all Australians." |
| Macgregor & Payne 2011 [90] | Increase in use of methamphetamine | NSW, NT, QLD, SA, VIC, WA | Adults in police custody at one of nine DUMA program data collection sites between 1999 and 2011. | Government Agency Report | 42079 | To report on the use of methamphetamine among police detainees between 1999 and 2011. | A retrospective quantitative analysis of DUMA program surveillance data. | Trend analysis demonstrates that after falling to a low of 13 percent in 2009, methamphetamine use among police detainees has once again increased—with 21 percent of detainees reporting methamphetamine use, according to data collected so far in 2011 |
| Sweeney & Payne 2011 [91] | Alcohol and assault on Friday and Saturday nights: Findings from the DUMA program | NSW, NT, QLD, SA, VIC, WA | Assault offenders in police custody at any one of nine DUMA interview sites between the hours of 6pm and 6am Friday night and 6pm and 6am on Saturday night in 2009 and 2010. | Government Agency Report | 170 | To examine the nature of alcohol consumption by assault offenders detained by the police and interviewed as part of the AIC’s DUMA program. | A retrospective analysis of a subset of DUMA program data, specifically alcohol consumption and crime attribution data from detainees charged with assault on Friday and Saturday evenings. | The results show that detainees charged with assault on a Friday and Saturday night are substantially different to those detainees charged at all other times and days of the week. In particular, assault offenders detained over the weekend were more likely to have consumed alcohol in the 48 hours prior to their arrest. |
| Sweeney & Payne 2011 [92] | Alcohol and disorderly conduct on Friday and Saturday nights: Findings from the DUMA program | NSW, QLD, VIC, WA, NT, SA | Police detainees in one of nine DUMA data collection sites detained for disorderly conduct on either a Friday or Saturday night between June 2009 and December 2010. | Government Agency Report | 161 | To examine the prevalence and nature of alcohol consumption by a sample of alleged offenders detained by the police during Friday and Saturday nights. | A retrospective quantitative analysis of a subset of DUMA program data collected on certain days was completed. | The results show that detainees charged with disorderly conduct on a Friday or Saturday night are substantially different to those detainees charged at other times and on other days. In particular, disorderly conduct offenders detained over the weekend were more likely to have consumed alcohol in the 48 hours prior to their arrest, to have mixed alcoholic drinks, and to be aged between 18 and 25 years. |
| Sweeney & Payne 2011 [93] | Victimisation and fear of crime among a sample of police detainees: Findings from the DUMA program | NSW, QLD, WA, SA, VIC, NT | Police detainees in one of eight DUMA data collection sites in the second quarter of 2010 that were administered DUMA program addendum questions. | Government Agency Report | 816 | To examine the self-reported victimisation and fear of victimisation for three crime types—physical assault, burglary and motor vehicle theft, among police detainees. | Addendum questions on victimization e were administered in addition to the usual DUMA self-report survey and urinalysis. | A substantial proportion of alleged offenders in the criminal justice system are also victims of crime and the expectation of future victimisation among police detainees was higher than or equal to the prevalence of actual victimisation in the past 12 months. |
| Payne & Gaffney 2012 [94] | How much crime is drug or alcohol related? Self-reported attributions of police detainees | NSW, NT, QLD, SA, VIC, WA | Individuals in police custody in one of the nine participating DUMA sites in the second half of 2009. | Government Agency Report | 1884 | To examine the self-reported alcohol and drug attributions of police detainees across Australia. | Retrospective analysis of new questions on detainees’ attribution of alcohol and drug use to their current detention added to the DUMA survey from 2010. | Nearly half of all police detainees attributed their current offending to alcohol or drugs—alcohol being more frequently attributed to by detainees than all other drugs combined. |
| Forsythe & Gaffney 2012 [95] | Mental disorder prevalence at the gateway to the criminal justice system | NSW, QLD, WA | Police detainees in one of the five DUMA data collection sites that administered the addendum survey on mental health. | Government Agency Report | 690 | The aims of this study are to 1) describe the mental disorder diagnoses reported by detainees, 2) estimate the unmet need for comprehensive psychological assessment and/or treatment among detainees, and 3) describe the illicit drug use and offending patterns of detainees with mental disorders. | A self-report survey detailing a range of criminal justice, demographic, drug use and drug market participation information as well as voluntary urinalysis. The mental health addendum included self-reported mental health and the use of a mental health screening tool. | This study found that a high proportion of people arrested by police had been previously diagnosed with mental disorders. |
| Sweeney & Payne 2012 [96] | ‘Initiation into drug use’ addendum: Findings from the DUMA program | NSW, QLD, SA, VIC, WA | Adult police detainees at one of eight DUMA program data collection sites that were administered the addendum questions on drug use initiation in 2009. | Government Agency Report | 842 | To understand why drug-using detainees first experimented with illicit drugs. | Addendum questions on drug use initiation were administered in addition to the usual DUMA self-report survey and urinalysis. | For these detainees, the two most frequently recorded reasons for experimenting with drugs was peer pressure or personal curiosity—a finding that is generally consistent for cannabis, speed and illegal opiates, and similar to the results of other surveys of drug users |
| Macgregor & Payne 2012 [97] | Cannabis use and mental health: Findings from a sample of offenders in police custody | NSW, QLD, WA | Police detainees in one of the five DUMA data collection sites that administered the addendum survey on mental health in quarter 1 and quarter 4 of 2010. | Government Agency Report | 1873 | To identify individuals in police custody that were likely to be experiencing a mental health related disorder. | Addendum questions on mental health were administered using the CMHS screening tool, in addition to the usual DUMA self-report survey and urinalysis. | It appears that cannabis use is a statistically significant and independent predictor of the likelihood that a police detainee will be assessed as having a probable mental health-related disorder requiring further psychological assessment. |
| Sweeney & Macgregor 2012 [98] | Decrease in use of ecstasy/MDMA | NSW, NT, QLD, SA, VIC, WA | Individuals in police custody at DUMA sites that self-reported use of MDMA. | Government Agency Report | 187 in 2010 and 170 in 2011. | To provide an update on police detainee ecstasy use in 2010 and 2011. | Retrospective analysis of a subset of police detainee data collected through the DUMA program. | An examination of the trend data revealed that after reaching a peak of 11 percent in 2009 (n=428), the prevalence of recent ecstasy use among police detainees has since halved - down to five percent in both 2010 (n=187) and 2011 (n=170). Self-reported data suggested that ecstasy had become harder to obtain, that there were fewer dealers selling the drug and that the quality was perceived to have declined. |
| Sweeney & Payne 2012 [99] | Drug use monitoring in Australia: 2009–10 report on drug use among police detainees | NSW, NT, QLD, SA, VIC, WA | Police detainees in one of the nine DUMA data collection sites in 2009 and 2010. | Government Agency Surveillance Report | 7575 adults and 186 juveniles | To describe the key results from the DUMA data collected throughout 2009 and 2010 | A combination of a self-report survey detailing a range of criminal justice, demographic, drug use and drug market participation information and voluntary urinalysis. | N/A |
| Bradford & Payne 2012 [100] | Illicit Drug Use and Property Offending among Police Detainees | NSW, NT, QLD, SA, VIC, WA | Police detainees interviewed at participating DUMA sites between 2008 and 2010 who had at least one charge recorded at the time of arrest. | Government Agency Report | 9453 | The primary objective of the current study was to examine whether the frequency of recent illicit drug use is related to higher levels of offending among police detainees in Australia. In particular, the study investigated whether the frequency of property offending escalates with offenders’ self-reported illicit drug use. | A retrospective analysis of a subset of DUMA data was completed. Authors analysed the association between explanatory demographic and drug use variables and their outcome of interest - the number of property charges against a detainee at the time of arrest. | These outcomes clearly demonstrate that heavy drug use, of either amphetamines or opioids in the 30 days prior to arrest, is associated with frequency of property offending. This has important implications for the treatment of drug using offenders within the criminal justice system. |
| Ng & Macgregor 2012 [101] | Pharmaceutical drug use among police detainees | NSW, NT, QLD, SA, VIC, WA | Adult police detainees in one of the nine DUMA data collection sites in Q3 of 2011. | Government Agency Report | 825 | To provide updated information about the prevalence of pharmaceutical drug use, the extent to which such drugs are obtained through illegal or illegitimate means, and perceptions of their availability. | A retrospective quantitative analysis of a subset of DUMA program data on pharmaceutical drug use. | Overall, the results indicated that 36 percent of detainees reported using at least one of five different pharmaceutical drug types in the past 12 months. One in three of these detainees (or 13% of all detainees) had access to the drug via a legitimate prescription given to them by a doctor; while the remaining two thirds of users (or 23% of all detainees) had sourced at least some of their pharmaceutical drugs through illegitimate means. |
| Forsythe 2013 [102] | Measuring mental health in criminology research: Lessons from the Drug Use Monitoring in Australia program | NSW, QLD, WA | Police detainees in one of the five DUMA data collection sites that administered the addendum survey on mental health. | Government Agency Report | 690 | To describe and discuss the process and challenges inherent in measuring mental health concerns among alleged offenders in police custody. The report also describes the results from a trial of using the Corrections Mental Health Screen instrument on police detainees in DUMA. | A self-report survey detailing a range of criminal justice, demographic, drug use and drug market participation information as well as voluntary urinalysis. The mental health addendum included self-reported mental health and the use of a mental health screening tool. | Measuring the prevalence of mental disorders is not a straightforward task and is all the more complex in field research that takes place in the criminal justice system. When researchers including cueing in their questions about mental health conditions it had two impacts; first, it defined what was meant by the term mental health problems and therefore increased the number of detainees who reported having been diagnosed and second, it appeared to remind detainees of additional diagnoses. |
| Lyneham & Chan 2013 [103] | Deaths in custody in Australia to 30 June 2011: Twenty years of monitoring by the National Deaths in Custody Program since the Royal Commission into Aboriginal Deaths in Custody | NSW, VIC, QLD, WA, SA, TAS, NT, ACT | Individuals who died while in custody (police or prison), or who died while police or custodial officers attempted to detain them in from 1980 to mid-2011. | Government Agency Surveillance Report | From 1980 to mid-2011 903 deaths occurred in police custody and custody-related operations, of which 521 were category 1 deaths. | To present information on the nature and circumstances of death in crime-related custodial settings - prison, juvenile justice and police custody that have occurred in Australia between 1 January 1980 and 30 June 2011. | Data collected by the NDICP between 1980 to mid-2011 is summarised and presented in tables and figures. | In a similar fashion to the long-term trends seen in prison custody deaths, police custody deaths have also exhibited a curvilinear trend. Specifically, from 1989–90 to the peak in 2001–02, the number of deaths increased, after which they began to decline each year, with the number recorded in both 2009–10 and 2010–11 being at the lower end of historical totals. |
| Community Development and Justice Standing Committee (Parliament of Western Australia) 2013 [104] | In Safe Custody: Inquiry into Custodial Arrangements in Police Lock-ups | WA | N/A | Government Investigation or Inquiry | N/A | To assess whether the current arrangements for police custody in WA fully comply with the recommendations of the RCIADIC and the OPCAT. To assess detainees access to medical and legal services, as well as the design of police lock-ups, and training of custodial staff. Oversight mechanisms were also reviewed. | This inquiry included the analysis of submissions, the testimony of witnesses, and the content of public hearings. Investigators also completed site visits and inspections of lock-ups in regional and metropolitan WA. | The inquiry identified 50 key findings and makes 22 recommendations across detainee access to medical services, legal services, the conditions of police lock-ups, the oversight of lock-up operations and the training of custodial staff. |
| Payne et al., 2013 [105] | Attitudes and perceptions towards drug driving amongst a sample of cannabis using police detainees | NSW, NT, QLD, SA, VIC, WA | Police detainees at participating DUMA data collection sites in 2008 who self-reported driving in the past 12 months. | Government Agency Report | 562 | To present findings related to drug driving derived from a sample of police detainees surveyed as part of DUMA. | Addendum questions on drink and drug driving were administered in addition to the usual DUMA self-report survey and urinalysis. | The findings from this study reinforces that drug driving, and in particular driving after the use of cannabis, is an ongoing concern to law enforcement and other criminal justice policy makers and practitioners. Relatively high rates of recent drug use were found among those who were driving at the time of their arrest (35%) as well as relatively high rates of self-reported cannabis drug driving among all police detainees (19%), the majority of whom admitted drug driving at least once a week, and often more frequently. |
| Victorian Ombudsman 2014 [106] | Investigation into deaths and harm in custody | VIC | N/A | Government Investigation or Inquiry | N/A | To determine the prevalence and nature of deaths and the incidence of harm in custody. To determine the factors contributing to deaths and harms in custody. To assess whether responsible agencies are taking adequate steps to minimise deaths in custody. To identify what more can be done to minimise the risk of deaths in custody. | Visiting prisons, police cells, and juvenile justice facilities, interviewing detainees, witnesses and family members, examining documentation from the Department of Justice, Department of Human Services, Victoria Police, and Forensicare. | The investigation identified a number of common issues impacting on the ability of custodial operators to ensure the safety and wellbeing of people in their care. These included: overcrowding, the management of detainees at risk of self-harm, transitional support services, access to health care, and accountability. |
| Doyle et al., 2014 [107] | Victorian Auditor-General’s Report: Prisoner Transportation | VIC | N/A | Government Investigation or Inquiry | N/A | To assess how effectively, efficiently and economically prisoners have been transported throughout Victoria’s criminal justice system. | The audit methodology included:  1) A desktop review of prisoner transportation and prisoner health 2) A review of the relevant policies and legislation  3) quantitative data analysis 4) examination of current contracts in place for prisoner transport  5) Interviews with agencies (Corrections Victoria, Victoria Police, and contractors). | There is no overarching and coordinated oversight of prisoner transportation across the justice system. Governance and risk management processes do not cover the full movement of prisoners within the justice system but focus on each area separately and in isolation from each other. |
| Coghlan & Goldsmid 2015 [108] | Findings from the DUMA program: Methamphetamine drug market trends | NSW, QLD, SA, WA | Adult police detainees in one of the six DUMA data collection sites in 2014. | Government Agency Report | 2310 | To examine recent trends in the use, availability, purity and price of methamphetamine based on Australian police detainee reports obtained via the DUMA program in 2014. | A combination of a self-report survey detailing a range of criminal justice, demographic, drug use and drug market participation information and voluntary urinalysis. | An increasing number of police detainees have tested positive via urinalysis to methamphetamine since 2009. In 2014, 37 percent of police detainees tested positive. The upward trend in methamphetamine use among police detainees evidenced in the DUMA data is contrary to the reported prevalence of use in the general population, which has remained relatively stable. |
| Ng et al., 2015 [109] | Drug use monitoring in Australia: 2011–12 report on drug use among police detainees | NSW, NT, QLD, SA, VIC, WA | Individuals over the age of 18 in police custody in DUMA data collection sites that consented to an interview and urinalysis. | Government Agency Surveillance Report | 7591 adults and 130 juveniles | To describe the key results from the DUMA data collected January 2011 to December 2012 from the nine different sites. | A combination of a self-report survey detailing a range of criminal justice, demographic, drug use and drug market participation information and voluntary urinalysis. | Overall, across the 2011–12 period, the most notable trend in illicit drug use within the Australian detainee sample was an increase in prevalence of use of amphetamines. A slight decrease in prevalence of use was observed for cannabis, which has continued on a downwards trend since 1999 and heroin. The prevalence of use of benzodiazepines, cocaine and MDMA remained relatively stable across the period. |
| Gannoni et al., 2015 [110] | Methamphetamine in Brisbane: Perspectives from DUMA police detainees | QLD | Adult police custody detainees in Brisbane Police Watch House in quarter 1 and 2 of 2015. | Government Agency Report | 381 | To provide information on what methamphetamine is, the nature and extent of use among Brisbane police detainees, and details of the Brisbane amphetamine market. | An analysis of a subset of DUMA data collected exclusively from Brisbane Police Watch House. | In 2015, almost half of all police detainees (43%) at the Brisbane Police Watch House who provided urine samples to the DUMA program tested positive to methamphetamine. This is the highest recorded rate of methamphetamine use among Brisbane detainees in DUMA’s  history. |
| Baker & Cussen 2015 [111] | Deaths in custody in Australia: National Deaths in Custody Program 2011–12 and 2012–13 | NSW, VIC, QLD, WA, SA, NT, TAS | Individuals who have died while in custody (police or prison), or who have died while police or custodial officers attempted to detain them between 2011 - 2013. | Government Agency Surveillance Report | 144 deaths in custody, 73 in 2011–12 (31 police custody and custody-related operations) and 71 in 2012–13 (18 police custody and custody-related operations). Altogether four of the deaths occurred in institutional police settings. | To report on deaths in prison custody, as well as deaths in police custody and custody-related operations across Australian states and territories for the 2011–12 and 2012–13 financial years. | A summary of data collected by the NDICP for the years 2011-12 and 2012-13. | N/A |
| Coghlan et al., 2015 [112] | Drug use monitoring in Australia: 2013–14 report on drug use among police detainees | NSW, QLD, SA, WA | Police detainees in one of the six DUMA data collection sites. | Government Agency Surveillance Report | 3456 adults and 41 juveniles | To describe the key results from the Drug Use Monitoring in Australia program from July 2013 to December 2014 from the six included sites. | A self-report survey detailing a range of criminal justice, demographic, drug use and drug market participation information as well as voluntary urinalysis. | Overall, in 2013–14 the most notable trend in illicit drug use within the Australian detainee sample was a 13 percent increase in detainees testing positive to amphetamines. There was also a 4% increase in detainees testing positive to at least one drug type |
| Brown 2015 [113] | Explaining the property crime drop: The offender perspective | NSW, NT, QLD, SA, VIC, WA | Police detainees in one of the eight DUMA data collection sites that answered the addendum survey questions on property crime in 2012. | Government Agency Report | 470 | To develop plausible hypotheses to explain the recent property crime drop in Australia by asking those that are closest to offending behaviour (police detainees) what factors may have contributed to the decline. | Addendum questions on property crime were administered in addition to the usual DUMA self-report survey and urinalysis. | Among the 470 police detainees who gave a response, 15% (n=72) disputed that property crime had dropped, believing it had in fact increased. Improvements in security were identified more often than any other reason for the property crime drop. |
| Coghlan & Goldsmid 2015 [114] | Findings from the DUMA program: Impact of reduced methamphetamine supply on consumption of illicit drugs and alcohol | NSW, QLD, SA, WA | Police detainees at participating DUMA data collection sites who reported using methamphetamine in the previous 12 months. | Government Agency Report | 259 | To assess whether methamphetamine users had previously experienced a period when methamphetamine was hard to get and whether this had affected the quantity of illicit drugs or alcohol they used at that time. | Addendum questions on the availability of methamphetamine were administered in addition to the usual DUMA self-report survey and urinalysis. | In conclusion, the findings suggest that harm minimisation may be realised among the methamphetamine-using population through policy and law enforcement efforts aimed at reducing methamphetamine supply. However, a significant proportion of detainees reported never experiencing a shortage of methamphetamine. This indicates that methamphetamine has remained readily available across Australia, despite an increased number of seizures by law enforcement. |
| Goldsmid et al., 2015 [115] | Findings from the DUMA program: Drink and drug driving among police detainees | NSW, QLD, SA, WA | Police detainees at participating DUMA data collection sites in 2014 who self-reported driving in the past 12 months. | Government Agency Report | 285 | To examine whether the deterrence preconditions of a perceived likelihood of experiencing random roadside drug testing and perceived legitimacy of random roadside drug testing, via a recognised association between illicit drug use and impaired driving ability, are present in a police detainee sample. | Addendum questions on drink and drug driving were administered in addition to the usual DUMA self-report survey and urinalysis. | The findings suggest that detainees do perceive a risk related to drink and drug driving, in terms of impairment in driving ability and potential detection by police. These conditions are likely to result in deterrence from drink and drug driving for some detainees. However, there was marked variability in the strength of these relations across substances. |
| Patterson et al., 2015 [116] | Methamphetamine in Perth: Perspectives from DUMA police detainees | WA | Police detainees in Perth watch house in quarter 1 and 2 of 2015. | Government Agency Report | 1111 | To provide information about what methamphetamine is, the nature and extent of use of methamphetamine among Perth police detainees, and details of the Perth methamphetamine market can assist police to identify risks to themselves and others. | A combination of a self-report survey detailing a range of criminal justice, demographic, drug use and drug market participation information and voluntary urinalysis. | In 2015, almost half of all police detainees (43%) at the Perth watch house who provided urine samples to the DUMA program tested positive to methamphetamine. This means that frontline police and watch house auxiliary staff are likely to be in regular contact with methamphetamine users. |
| Kapira et al., 2015 [117] | Methamphetamine in Adelaide: Perspectives from Police Detainees | SA | Individuals in police custody at the Adelaide Watch House in the first half of 2015. | Government Agency Report | 239 | To describe the trends in methamphetamine usage among police detainees in Adelaide. | A combination of a self-report survey detailing a range of criminal justice, demographic, drug use and drug market participation information and voluntary urinalysis. | N/A |
| Payne et al., 2015 [118] | Homelessness and housing stress among police detainees: Results from the DUMA program | NSW, QLD, WA, SA, VIC, NT | Individuals in police custody at one of nine DUMA program data collection sites in the second quarter of 2011. | Government Agency Report | 946 | To examine the prevalence and nature of homelessness among a sample of police detainees. | A retrospective quantitative analysis of DUMA program data collected in 2011. | By expanding questioning around accommodation to include secondary and tertiary homelessness this study showed that the estimated prevalence of homelessness among police detainees to be 22%. |
| Independent Broad-based Anti-Corruption Commission 2016 [119] | Operation Ross: An investigation into police conduct in the Ballarat Police Service Area | VIC | All four women described in the investigation were victims of alleged excessive force by members of Ballarat Police Station. One while held in the cells, and three while visiting reception who were subsequently taken into custody. | Government Investigation or Inquiry | 4 | To investigate allegations of excessive force used against four women at the Ballarat watch house. To assess whether any human rights had been violated, and the appropriateness of internal reporting and handling by Victoria Police. | Public examination was undertaken at Ballarat Law Courts in May 2016. Statements taken from 13 witnesses involved in the cases. | IBAC made four recommendations to improve Victoria Police processes for handling complaints, enhancing officer training, and enhancing officer understanding of policy and guidelines of appropriate practice. |
| Fuller et al., 2016 [120] | Managing intoxicated offenders: Best practice in responding to individuals affected by drugs and alcohol | NSW, QLD, SA, WA | Multiple populations are discussed in this report. Firstly, individuals that have died in police custody between 2002 and 2011 while intoxicated with drugs or alcohol. Secondly, detainees interviewed through the DUMA program in 2014 whose data could be correlated with police records. | Government Agency Report | The cases of 41 individuals who died in custody is reviewed including 12 that died in the watch house. Quantitative analysis of DUMA program data of 216 detainees is also included. | To enhance the understanding of effective strategies to manage intoxicated offenders in custody and to develop a best practice framework to guide police practice. | Authors undertook a broad range of methods in this report. Police policies and procedures in NSW, QLD, WA, and SA were examined qualitatively. Quantitative and qualitative analyses were completed for publicly available coroner's reports for 2002-2011, where the individual died in police custody, had alcohol or drugs in their system, and did not die as part of a motor vehicle pursuit. Quantitative analysis of DUMA program data was completed. Focus group consultations were completed with police representatives, and a national roundtable was held in Canberra in April 2015. | Most individuals in police custody have alcohol or other drugs in their system, to some degree. While in custody, police officers are responsible for the safety and wellbeing of offenders, and this duty of care includes managing intoxication and withdrawal-related risks. The research team proposed the CALM framework to assist police identify and manage the risks associated with intoxication and withdrawal. |
| Patterson et al., 2016 [121] | Methamphetamine in Sydney: Perspectives from DUMA police detainees | NSW | Individuals in police custody at one of the participating Sydney sites from 2014 to 2015. | Government Agency Report | 171 | To provide information on the nature and extent of use of methamphetamine among Sydney police detainees. | Retrospective analysis of data collected from the two Sydney based DUMA sites. | In 2015, a substantial proportion of detainees who provided urine samples to the DUMA program at both the Bankstown (29%) and Surry Hills police stations (53%) tested positive to methamphetamine. |
| Gately et al., 2016 [122] | Drug Use Monitoring in Australia (DUMA): An expansion into the Pilbara, Western Australia | WA | Police detainees in Perth or South Hedland DUMA program data collection sites in 2012 and 2013. | Government Agency Report | 260 | To provide empirical evidence on the role of alcohol and other drugs, and a clear picture of the local drug market, in a remote regional town in Western Australia. | Data from Perth and South Hedland DUMA program data collection sites from 2012 and 2013 were compared with a focus on substance use and criminal behaviour. | The findings suggest the holistic approach to dealing with higher levels of substance use in the region should continue, with a focus on reducing demand through treatment and interventions, reducing alcohol supply through restrictions, and minimising the harm of high levels of alcohol use through education. |
| Fuller & Goldsmid 2016 [123] | The nature of risk during interactions between the police and intoxicated offenders | Multiple States or Territories – Not stated | Individuals who died in police custody between 2002 and 2011 with alcohol or illicit drugs in their system at the time of death based on the autopsy report. | Government Agency Report | 41 including 9 that died in cells | To examine how intoxication influences the nature and magnitude of the risk associated with police officer/offender interactions. | Information contained in coroner’s reports was extracted, coded and analysed qualitatively. | While the offenders’ intoxication was not the primary reason for their involvement with the police, intoxication is an important determinant of the level of risk inherent in the interaction. In line with previous research, these findings demonstrate that risk levels are related to intoxication via the effect of alcohol and other drugs on offenders’ behaviour and health. |
| Goldsmid & Willis 2016 [124] | Methamphetamine use and acquisitive crime: Evidence of a relationship | NSW, QLD, SA, WA | Police detainees in one of five DUMA program data collection sites in quarter 3 and 4 of 2013. | Government Agency Report | 1146 | This paper examines the engagement in acquisitive crime, and perceived motivations for methamphetamine driven crime, of a sample of Australian police detainees recruited in 2013 through the Drug Use Monitoring in Australia program. | A retrospective quantitative analysis of DUMA program data collected in 2013 was undertaken. Sociodemographic data from methamphetamine users were compared to non-users. | Methamphetamine users reported deriving a significantly higher proportion of their income from crime than non-users. Logistic regression analysis reveals the use of methamphetamine, heroin and/or cannabis predicts engagement in acquisitive crime when other drug use and polydrug use is controlled for. |
| The State of Queensland (Crime and Corruption Commission) 2017 [125] | Police use of force in Queensland watch-houses | QLD | The population assessed in the CCTV component of the report, were detainees at one of 5 police watch houses across Queensland, including one located in an Indigenous community. | Government Investigation or Inquiry | A subset of the investigation involved reporting on detainees using CCTV - the entrance of 331 detainees into watch-houses was reviewed in this section. | To improve understanding of the nature and frequency of force being used by officers in Queensland watch-houses, examine injuries to people detained in a watch-house resulting from the use of force and assess compliance with QPS use of force and injury reporting requirements. | Investigators examined CCTV footage from five watch-houses, reviewed QPS administrative data, and Crime and Corruption Commission allegations. | The report found that on average, 3 out of every 10 detainees experienced some use of force at the front charge counter, and that a considerable proportion of the uses of force involved compliant detainees. No detainee injuries were identified by the review, but 6 recommendations for improvement were made to QPS. |
| White & Gooda 2017 [126] | Chapter 25 The path into detention *In* Report of the Royal Commission and Board of Inquiry into the Protection and Detention of Children in the Northern Territory | NSW | Juveniles in the Northern Territory | Government Investigation or Inquiry | N/A | To assess how children were treated in detention centres in the Northern Territory and to also look into the welfare system in the Northern Territory. | A royal commission including submissions, interviews, and inspections. The section on arrest and police custody included descriptions of and inspections of watch-houses. | Chapter 25 includes 43 recommendations across police, diversion, bail and court treatment for juveniles. |
| Gannoni & Goldsmid 2017 [127] | Readiness to change drug use and help-seeking intentions of police detainees: Findings from the DUMA program | NSW, QLD, SA, WA | Adult police detainees in one of four DUMA program data collection sites in the second quarter of 2014 who reported the use of at least one drug in the previous 12 months. | Government Agency Report | 321 | To explore the readiness to change drug use and help-seeking intentions of Australian police detainees with drug problems. | Addendum questions on drug dependence and readiness to change were administered in addition to the usual DUMA self-report survey and urinalysis. Dependence was measured with the MINI Plus tool, readiness to change was assessed with the RCQ-D tool, and help seeking intentions were assessed with the GHSQ tool. | The analysis revealed those detainees most in need of drug treatment were also those most ready to change their drug use. The findings serve as a reminder of the need and desire for interventions for drug abuse among the police detainee population and have implications for the development of intervention strategies aimed at reducing drug use among offender populations. |
| Patterson & Sullivan 2018 [128] | The cannabis market: Police detainee perspectives | NSW, QLD, SA, WA | Adult police detainees held in participating sites in Brisbane, Adelaide, Perth and Sydney who reported using cannabis in the previous 12 months. | Government Agency Report | 2559 | To report on the trends in cannabis use among police detainees. | A combination of a self-report survey detailing a range of criminal justice, demographic, drug use and drug market participation information and voluntary urinalysis. | Despite a general decline in cannabis use, the continuing popularity of cannabis among the police detainee population indicates a need to understand the nature and extent of use. |
| Sullivan & Patterson 2018 [129] | The ecstasy market: Police detainee perspectives | NSW, QLD, SA, WA | Adult police detainees in DUMA data collection sites between January 2015 and December 2016. | Government Agency Report | 4400 | To provide new data on ecstasy use among a sample of Australian police detainees. | A retrospective analysis of DUMA data including the results of self-report surveys and urinalysis. | About one-sixth of detainees reported they had recently used ecstasy, although only two percent reported dependence. On average in 2015–16, recent users reported using ecstasy three days out of the last 30 days before detention and, on these days, their average consumption was one gram of ecstasy. |
| Ticehurst & Sullivan 2018 [130] | The methamphetamine market: police detainee perspectives | NSW, QLD, SA, WA | Adult police detainees in DUMA data collection sites between January 2015 and December 2016. | Government Agency Report | 4400 | To provide new data on methamphetamine use among a sample of Australian police detainees. | A retrospective analysis of DUMA data including the results of self-report surveys and urinalysis. | In 2015–16, most police detainees reported that they had used methamphetamine in the previous 12 months, and the majority of these had used the drug in the last 30 days. More than half of the detainees who reported use in the previous 12 months reported injecting the drug. Forty two percent of detainees who had used methamphetamine in the previous 12 months also reported being dependent. |
| Inspector of Custodial Services 2018 [131] | Inspection of 24-hour court cells in NSW | NSW | N/A | Government Investigation or Inquiry | N/A | To report on inspections completed in 2017 of all 24-hour court cells managed by Corrective Services NSW. | Onsite visits to each court cell facility were completed between May and June 2017. Investigators conducted interviews with management, police officers, custodial officers, nurses, detainees, and the NSW Ombudsman. Data from Custodial Services NSW and Justice Health and Forensic Mental Health Network was also used as part of the review. | This report makes 37 recommendations regarding the duration of admission, the conditions, the access to healthcare, and the maintenance of court cells in NSW. |
| Sullivan et al., 2018 [132] | Prescription opioid use among Australian police detainees | NSW, QLD, SA, WA | Police detainees at one of four DUMA program data collection sites in 2016 that administered the addendum question on prescription drug use. | Government Agency Report | 389 | To assess how prevalent non-medical prescription opioid use among Australian police detainees is, which opioids are used, and what the characteristics of users are. | Addendum questions on prescription drug use were administered in addition to the usual DUMA self-report survey and urinalysis. | One-quarter of detainees had used prescription opioids in the last 12 months and 71 percent of these users had sourced or diverted at least some of their opioids illegitimately. Detainees who reported recent non-medical use of prescription opioids were more likely to be male and were, on average, slightly older than those reporting prescribed-use only and those reporting use of other illicit drugs. |
| Patterson et al., 2018 [133] | Use and misuse of prescription drugs among police detainees | NSW, QLD, SA, WA | Adults in police custody at DUMA program data collection sites in January and February 2016. | Government Agency Report | 386 | To examine the patterns of prescription drug use among a sample of police detainees. | Addendum questions on prescription drug use were administered in addition to the usual DUMA self-report survey and urinalysis. Associations between prescription drug use and offending was then explored. | This paper provides additional evidence that non-medical prescription drug use is an important issue in Australia, disproportionately affecting police detainees. Forty percent of detainees had used prescription drugs non-medically in the previous 12 months, a level eight times higher than the level at which the Australian population reports non-medical use of pharmaceuticals, including prescription drugs. |
| Patterson & Sullivan 2018 [134] | The heroin market: Police detainee perspectives | NSW, QLD, SA, WA | Adult police detainees held in participating sites in Brisbane, Adelaide, Perth and Sydney who reported using heroin in the previous 12 months. | Government Agency Report | 521 | To report on the trends in heroin use among police detainees. | A combination of a self-report survey detailing a range of criminal justice, demographic, drug use and drug market participation information and voluntary urinalysis. | Although heroin use among police detainees continues to decrease, detainee comments and ratings of availability in 2015–16 indicate that heroin is becoming more readily available in the illicit drug market, demonstrating the importance of continuing to monitor trends in its use. |
| Ticehurst et al., 2018 [135] | National Deaths in Custody Program: Deaths in custody in Australia 2013–14 and 2014–15 | NSW, VIC, QLD, WA, SA, NT | Individuals who died while in custody (police or prison), or who died while police or custodial officers attempted to detain them in from 2013 to 2015. | Government Agency Surveillance Report | 34 deaths occurred in police custody and custody-related operations, of which 21 were category 1 deaths and 3 deaths occurred in police cells. | To provide the latest information on deaths in custody in Australia for the years 2013-14 and 2014-15. | Data collected by the NDICP for the years 2013-14 and 2014-15 are summarised and presented in tables and figures. | In 2013–14 the number of deaths in police custody and custody-related operations was the lowest in 26 years for both Indigenous and non-Indigenous people, with numbers increasing again slightly in 2014–15. |
| Sullivan & Patterson 2018 [136] | Fentanyl use by police detainees remains unchanged | NSW, QLD, SA, WA | Police detainees at participating DUMA data collection sites who reported using pharmaceutical opioids. | Government Agency Report | 1044 | To provide new data on the prevalence of fentanyl use among a sample of Australian police detainees. | Addendum questions on pharmaceutical opioid use were administered in addition to the usual DUMA self-report survey and urinalysis. | The percentage of police detainees reporting fentanyl use in the last 12 months appears unchanged at 3% from the previous time it was measured in 2016. |
| Patterson et al., 2018 [137] | Polydrug use among police detainees | NSW, QLD, SA, WA | Adult police detainees at DUMA program data collection sites in 2016. | Government Agency Report | 2199 | To investigate the extent of polydrug use and recent polydrug trends among police detainees. | A retrospective quantitative analysis of a subset of DUMA program data on polydrug use. | Urinalysis data indicated that detainees under-reported polydrug use, suggesting that more detainees may be at risk of the harms associated with polydrug use than survey data suggest. |
| Patterson et al., 2018 [138] | Drug use monitoring in Australia: 2015 and 2016 report on drug use among police detainees | NSW, QLD, SA, WA | Individuals in police custody in one of the five DUMA data collection sites in 2015 and 2016. | Government Agency Surveillance Report | 4445 | To describe the key results from the DUMA program data collected during 2015 and 2016. | A combination of a self-report survey detailing a range of criminal justice, demographic, drug use and drug market participation information and voluntary urinalysis. | N/A |
| Goldsmid 2019 [139] | Findings from the DUMA program: The influence of cannabis dependency and use on criminal offending, through the eyes of police detainees | WA, QLD, SA, NSW | Individuals in police custody at one of five DUMA program data collection sites in 2013. | Government Agency Report | 1149 | To report on the demographic characteristics and drug use findings of cannabis users captured in the 2013 DUMA program. | A retrospective quantitative analysis of DUMA program data collected in 2013 is completed. Data was collected using the usual DUMA self-report survey and urinalysis. | There appears to be a high degree of unmet need for treatment for cannabis dependency among Australian detainees. Almost all detainees who self-reported cannabis dependency also reported recent cannabis use. |
| Gannoni & Bricknell 2019 [140] | National Deaths in Custody Program: Deaths in custody in Australia 2015–16 | NSW, WA, QLD, SA, VIC, TAS, NT | Individuals who died while in custody (police or prison), or who died while police or custodial officers attempted to detain them in 2015-16 | Government Agency Surveillance Report | 20 deaths occurred in police custody and custody-related operations, of which 14 were category 1 deaths, and 0 occurred in police cells. | To provide the latest information on deaths in custody in Australia for the years 2015-16. | Data collected by the NDICP for the year 2015-16 is summarised and presented in tables and figures. | N/A |
| Gannoni & Bricknell 2019 [141] | National Deaths in Custody Program: Deaths in custody in Australia 2016–17 | NSW, QLD, WA, SA, NT | Individuals who died while in custody (police or prison), or who died while police or custodial officers attempted to detain them in 2016-17 | Government Agency Surveillance Report | 17 deaths occurred in police custody and custody-related operations, of which 10 were category 1 deaths and one death occurred in a police cell. | To provide the latest information on deaths in custody in Australia for the years 2016-17. | Data collected by the National Deaths in Custody Monitoring and Research Program for the year 2015-16 is summarised and presented in tables and figures. | N/A |
| Gannoni & Bricknell 2019 [142] | Indigenous deaths in custody: 25 years since the Royal Commission into Aboriginal Deaths in Custody | NSW, VIC, QLD, WA, SA, TAS, ACT, NT, External Australian Territory | Individuals who died while in custody (police, or prison detention), or who died while police or custodial officers attempted to detain them from the 1991-92 financial year to 2015-16. | Government Agency Surveillance Report | 2044 deaths in custody were included in the analysis. 744 deaths occurred in police custody including 146 deaths of Indigenous detainees. Of these, 64 were category 1 deaths. | To provide a picture of trends and characteristics of Indigenous deaths in prison and police custody in the 25 years since the RCIADIC. | A retrospective analysis of data from the NDICP database | In 1991, the RCIADIC concluded Indigenous people were no more likely to die in custody than non-Indigenous people but were significantly more likely to be arrested and imprisoned. The same remains true today. |
| Sullivan & English 2019 [143] | Is alcohol and energy drink consumption associated with antisocial behaviour? | NSW, QLD, SA, WA | Individuals in police custody at DUMA data collection sites in 2017. | Government Agency Report | 459 | To investigate the extent to which detainees reported having drunk alcohol and energy drinks at high-risk levels and compared this with the high-risk drinking of detainees who reported having consumed alcohol but not energy drinks. | Addendum questions on alcohol and energy drink consumption were administered in addition to the usual DUMA self-report survey and urinalysis. | Detainees engage in harmful behaviours, including criminal offences, while they drink alcohol and while they drink alcohol mixed with energy drinks. The high-risk drinking and other harmful behaviours that many in the sample reported reinforce the importance of court-based and mandated referral pathways into drug treatment, and prison-based alcohol treatment. |
| Patterson et al., 2019 [144] | Drug use monitoring in Australia: Drug use among police detainees, 2017 | NSW, QLD, SA, WA | Police detainees in one of the five DUMA data collection sites in 2017. | Government Agency Surveillance Report | 2293 | To report on the results of DUMA data collected in 2017. | A combination of a self-report survey detailing a range of criminal justice, demographic, drug use and drug market participation information and voluntary urinalysis. | N/A |
| Voce & Sullivan 2019 [145] | Drug use monitoring in Australia: Drug use among police detainees, 2018 | NSW, QLD, SA, WA | Police detainees in one of the five DUMA data collection sites in 2018. | Government Agency Surveillance Report | 2418 | To describes drug use, drug market participation and the extent to which police detainees’ alleged offences were related to drug or alcohol use. | A combination of a self-report survey detailing a range of criminal justice, demographic, drug use and drug market participation information and voluntary urinalysis. | N/A |
| Goldsmid 2019 [146] | Findings from the DUMA program: Impact of reduced cannabis supply on the consumption of illicit drugs and alcohol | WA, QLD, SA, NSW | Detainees in DUMA program data collection sites in quarter 3 of 2013 that indicated cannabis use in the previous 12 months. | Government Agency Report | 277 | To examine retrospective self-reports from cannabis users of the impact that periods of reduced cannabis supply had on their consumption of cannabis, alcohol and other illicit drugs. | Addendum questions on the cannabis market were administered in addition to the usual DUMA self-report survey and urinalysis. A quantitative analysis was then completed. | In terms of harm minimisation, supply-reduction approaches to cannabis appear to have merit. The majority of cannabis-using detainees reported when periods of reduced cannabis supply have been experienced, they either reduced consumption or abstained from cannabis. |
| Morgan & Gannoni 2020 [147] | Methamphetamine dependence and domestic violence among police detainees | NSW, NT, QLD, SA, VIC, WA | Male police detainees in one of eight DUMA program data collection sites in the fourth quarter of 2012 who reported being in a relationship. | Government Agency Report | 351 | To explore the relationship between methamphetamine dependence and domestic violence among male police detainees. | Addendum questions on domestic violence were administered in addition to the usual DUMA self-report survey and urinalysis. A subset of addendum respondents (males) answers were then analysed using multivariate logistic regression. | Overall, rates of self-reported domestic violence among drug dependent detainees were very high, with close to two-thirds of methamphetamine dependent detainees reporting that they had been abusive towards a current or former partner in the previous 12 months. The likelihood of recent domestic violence was significantly higher for methamphetamine dependent detainees than for detainees who had used but were not dependent on methamphetamine, even after other factors were taken into account. |
| Voce & Sullivan 2020 [148] | Why Australian police detainees choose to use (or not use) non-prescribed fentanyl | NSW, QLD, SA, WA | Police detainees in DUMA data collection sites in July and August 2019 that were administered the addendum questions on fentanyl use. | Government Agency Report | 566 | To better understand the motivations of Australian police detainees who use non-prescribed fentanyl. | Addendum questions on fentanyl use were administered in addition to the usual DUMA self-report survey and urinalysis. | This study suggests non-prescribed fentanyl use is driven by the same motivations underlying other forms of illicit drug use, particularly a desire for intoxication, easing withdrawal from other drugs, and self-medicating physical and psychological distress. |
| Voce & Sullivan 2020 [149] | Is there fentanyl contamination in the Australian illicit drug market? | NSW, QLD, SA, WA | Police detainees in DUMA data collection sites in July and August 2019 that were administered the addendum questions on fentanyl use. | Government Agency Report | 566 | To investigate fentanyl use among police detainees participating in the Drug Use Monitoring in Australia program. | Addendum questions on fentanyl use were administered in addition to the usual DUMA self-report survey and urinalysis. | These results provide an early warning of possible unintended fentanyl use in Australia, particularly among people who use other illicit drugs. The study also provides evidence of the combined use of fentanyl and methamphetamine, which may represent intentional use of fentanyl to ease withdrawal  symptoms or to produce an intense high. |
| Doherty & Sullivan 2020 [150] | Drug Use Monitoring in Australia: Drug use among police detainees, 2019 | NSW, QLD, SA, WA | Adult police detainees in one of five DUMA program data collection sites in 2019. | Government Agency Surveillance Report | 2230 | To describe the key results from the DUMA program data collected during 2019. | A combination of a self-report survey detailing a range of criminal justice, demographic, drug use and drug market participation information and voluntary urinalysis. | N/A |
| Doherty & Sullivan 2020 [151] | How and where police detainees obtain methamphetamine | NSW, QLD, WA | Police detainees aged 18 and over at participating DUMA data collection sites who self-reported using methamphetamine in the past 12 months. | Government Agency Report | 672 | This study seeks to understand the types of markets from which a group of drug users in Australia obtain methamphetamine, and how this varies according to demographic factors and patterns of use. | Addendum questions on where methamphetamine was sourced from were administered in addition to the usual DUMA self-report survey and urinalysis. | The findings suggest detainees most commonly obtained methamphetamine through closed drug markets using social supply. However, detainees with more harmful patterns of use also commonly used semi-open markets. |
| Doherty & Bricknell 2020 [152] | Deaths in custody in Australia 2017–18 | NSW, VIC, QLD, WA, SA, ACT | Individuals who have died while in custody (police or prison), or who have died while police or custodial officers attempted to detain them in 2017-18. | Government Agency Surveillance Report | 21 deaths in police custody and custody-related operations including 17 category 1 deaths and 3 deaths that occurred inside police cells. | To provide the latest information on deaths in custody in Australia for the years 2017-18. | Data collected by the NDICP for the year 2017-18 is summarised and presented in tables and figures. | N/A |
| Doherty & Bricknell 2020 [153] | Deaths in custody in Australia 2018–19 | WA, NSW, VIC, QLD, SA, TAS | Individuals who have died while in custody (police or prison), or who have died while police or custodial officers attempted to detain them in 2018-19. | Government Agency Surveillance Report | 24 deaths in police custody and custody-related operations including 16 category 1 deaths, but none of the deaths occurred inside police cells for this reporting period. | To provide the latest information on deaths in custody in Australia for the years 2018-19. | Data collected by the NDICP for the year 2018-19 is summarised and presented in tables and figures. | N/A |
| Sullivan & Voce 2020 [154] | The social supply of pharmaceutical opioids | NSW, QLD, SA, WA | Adult police detainees in one of five DUMA program data collection sites that administered the addendum questions on pharmaceutical opioid use in 2018. | Government Agency Report | 1195 | To explore the prevalence, predictors and nature of the ‘social supply’ of pharmaceutical opioids among police detainees. | Addendum questions on pharmaceutical opioid use were administered in addition to the usual DUMA self-report survey and urinalysis. | More than half of the police detainees who had used pharmaceutical opioids for non-medical purposes in the past 12 months had accessed these drugs through social supply. Almost all of these individuals had sourced the opioids from family and friends for free, and a smaller number had swapped other drugs for them. A substantial proportion of detainees also bought pharmaceutical opioids from family and friends. |
| Voce et al., 2020 [155] | COVID-19 pandemic constricts methamphetamine supply in Perth | WA | Individuals in police custody in Perth in Q2 of 2020. | Government Agency Report | 145 | To examine the methamphetamine market and prevalence of methamphetamine use in Perth during the COVID-19 pandemic. | Data collection from individuals in police custody in Perth through the DUMA program were compared to historical DUMA data from 2017-19. | This study suggests the unprecedented economic and social impacts of the COVID-19 pandemic in Australia may have had significant consequences for Perth’s methamphetamine market. Detainees reported that methamphetamine had become extremely difficult to source in Perth, with fewer dealers selling or sharing the drug. |
| Voce & Sullivan 2021 [156] | Drug use monitoring in Australia: Drug use among police detainees, 2020 | NSW, QLD, SA, WA | Adult police detainees in one of five DUMA program data collection sites in 2020. | Government Agency Surveillance Report | 1754 | To describe the key results from the DUMA program data collected during 2020. | A combination of a self-report survey detailing a range of criminal justice, demographic, drug use and drug market participation information and voluntary urinalysis. | N/A |
| Doherty & Sullivan 2021 [157] | Deaths in custody in Australia 2019–20 | NSW, VIC, QLD, WA, TAS, NT | Individuals who have died while in custody (police or prison), or who have died while police or custodial officers attempted to detain them in 2019-20. | Government Agency Surveillance Report | 24 deaths in police custody and custody-related operations including 20 category 1 deaths. No deaths occurred in police cells. | To provide the latest information on deaths in custody in Australia for the years 2019-20. | Data collected by the NDICP for the year 2019-20 is summarised and presented in tables and figures. | N/A |
| Doherty 2021 [158] | Deaths in custody in Australia 2020–21 | NSW, QLD, VIC, TAS | Individuals who died while in custody (police, or prison detention), or who died while police or custodial officers attempted to detain them in 2020-21. | Government Agency Surveillance Report | 16 deaths in police custody including 11 category 1 deaths. No deaths occurred in police cells. | To present detailed tabulated information on deaths in custody in Australia for the year 2020-21 and to provide jurisdictional breakdowns for comparative purposes. | Data collected by the NDICP for the year 2020-21 are summarised and presented in tables and figures. | N/A |
| Doherty et al., 2021 [159] | Impact of the COVID-19 pandemic on cannabis demand and supply in Australia | NSW, QLD, SA, WA | Police detainees in participating sites in Perth, Brisbane, Adelaide and Sydney in July and August 2020. | Government Agency Report | 446 | To examine the impact of the COVID-19 pandemic on cannabis demand and supply in Australia. | Addendum questions on COVID-19 were administered in addition to the usual DUMA self-report survey and urinalysis. | This study suggested that overall cannabis availability and price did not change substantially but, the amount of cannabis consumed appeared to increase. |
| Law Enforcement Conduct Commission 2022 [160] | Operation Kimbla: Report to the parliament pursuant to section 132 Law Enforcement Conduct Commission Act 2016 | NSW | An indigenous juvenile in police custody in NSW (exact site undisclosed). | Government Investigation or Inquiry | 1 | This document reports on the Law Enforcement Conduct Commission's investigation (Operation Kimbla) of the treatment on a young Indigenous person in police cells. | Review of CCTV and interview of involved officers. | One officer was found to be engaged in serious misconduct in his treatment of the civilian. |
| Voce & Sullivan 2022 [161] | Drug use monitoring in Australia: Drug use among police detainees, 2021 | NSW, QLD, SA, WA | Adult police detainees in one of five DUMA program data collection sites in 2021. | Government Agency Surveillance Report | 2223 | To describe the key results from the DUMA program data collected during 2021. | A combination of a self-report survey detailing a range of criminal justice, demographic, drug use and drug market participation information and voluntary urinalysis. | N/A |
| McAlister & Bricknell 2022 [162] | Deaths in custody in Australia 2021-22 | NSW, QLD, VIC, WA, SA | Individuals who died while in custody (police, or prison detention), or who died while police or custodial officers attempted to detain them in 2021-2022. | Government Agency Surveillance Report | 22 deaths in police custody including 18 category 1 deaths. 3 deaths occurred in police cells. | To present detailed tabulated information on deaths in custody in Australia for the year 2021-22 and to provide jurisdictional breakdowns for comparative purposes. | Data collected by the NDICP for the year 2021-22 are summarised and presented in tables and figures. | N/A |
| Mazerolle et al., 2022 [163] | Independent review into investigations of police-related deaths, and domestic and family violence deaths in Queensland | QLD | One section of this report contains data on individuals that have died in police custody in Queensland. | NGO or Independent Report / Inquiry | The report includes discussion of 34 deaths in police custody in Queensland between 2011 and 2021, and 43 coroners reports of either deaths in police custody or police operations between 2012 and 2019. | This report was commissioned in response to the State Coroner’s recommendation that current arrangements for investigating deaths in police custody or deaths in the course of police operations should be reviewed. The primary concern raised is whether police should investigate the actions and conduct of other police.  A second focus was included in the review that tasked the review team to consider mechanisms for investigating domestic and family violence deaths where there had been prior police contact. | A broad methodological approach was taken combining quantitative and qualitative social science approaches and legal and policy analysis. Data gathered and analysed  included relevant policies, procedures, coronial reports, inquiry reports, stakeholder interviews and analysis of all in scope death investigations including those with prior police contacts where relevant. | Interviews with stakeholders and thematic review of coronial reports show that people generally view the Ethical Standards Command investigations to be of a high standard. However, the perception of ‘police investigating police’ is regarded as not acceptable to the community. The authors also found that the separate roles of the Ethical Standards Command, Crime Corruption Commission, and Coroner are not well understood even by those who participate regularly in these matters. The independent report makes 7 recommendations in relation to the investigation of deaths in custody, and 5 recommendations related to investigative mechanisms for family violence deaths with prior police contact. |
| Ombudsman NT 2023 [164] | Extraordinary restraint: Spit hood & emergency restraint chair use on children in police custody | NT | The investigation includes the review of 30 children who were held in police custody in the NT during 2020 or 2021 and who had either a spit hood or emergency restraint chair or both used on them while they were in custody. | Government Investigation or Inquiry | 30 | To assess the rationale, background, and merits of the ongoing use of spit hoods and emergency restraint chairs by the NT police on children, and to assess the adequacy of policies, training and oversight of their use. | The investigation involved a review of relevant documentation provided by NT police, and a review of all occasions where a spit hood or emergency restraint chair were used on children in police custody in 2020 and 2021. | This investigation recommends that NT Police should cease the use of spit hoods and should develop a plan for phasing out emergency restraint chairs in place of alternatives (as has already been done in youth detention facilities). |
| McAlister et al., 2023 [165] | Deaths in custody in Australia 2022–23 | NSW, SA, QLD, VIC, WA, TAS | Individuals who died while in custody (police, or prison detention), or who died while police or custodial officers attempted to detain them in 2022-2023. | Government Agency Surveillance Report | 40 deaths in police custody including 29 category 1 deaths. 2 deaths occurred in police cells. | To present detailed tabulated information on deaths in custody in Australia for the year 2022-23 and to provide jurisdictional breakdowns for comparative purposes. | Data collected by the NDICP for the year 2022-23 are summarised and presented in tables and figures. | N/A |
| Queensland Family & Child Commission 2023 [166] | Who’s responsible: Understanding why young people are being held longer in Queensland watch houses | QLD | Children held in watch houses in Queensland | Government Agency Report | 30 case files | To investigate why the amount, and length of stay of children in police custody in Queensland is increasing. This includes analysis of 30 individual case files of young people held for extended periods in watch houses, an analysis of legislation differences between Queensland and other states, and a statistics profile of key areas of the criminal justice system. | A quantitative and qualitative mixed methods approach was undertaken | The report makes 5 recommendations to Youth Justice, the Queensland Government, and Queensland Police Force aimed at reducing the duration that children are kept in police watch houses. |
| Office of the Queensland Ombudsman 2024 [167] | Cairns and Murgon watch-houses inspection report: focus on detention of children | QLD | Children in watch-houses in Queensland on the day that the Ombudsman inspected. | Government Agency Report | On the day of inspection, 11 children were held in Cairns watch-house and 3 were held in Murgon. Some were interviewed. Data from 381 child admissions to Cairns watch-house and 93 to Murgon watch-house are also reviewed. | To report on the inspection of the Cairns watch-house in January 2024 and Murgon watch-house in November 2023 by the Queensland Ombudsman. | Inspections were undertaken by the Ombudsman, and relevant police, government, and youth detention documentation was reviewed. Watch-house staff and detainees were interviewed. | The report highlights the significant risk of harm to children in watch-houses. It makes 19 recommendations to minimize or avoid these types of harms. |
| Miles et al., 2024 [168] | Deaths in custody in Australia 2023–24 | NSW, VIC, QLD, WA, TAS | Individuals who died while in custody (police, or prison detention), or who died while police or custodial officers attempted to detain them in 2023-2024. | Government Agency Surveillance Report | 27 deaths in police custody including 18 category 1 deaths. 2 deaths occurred in police cells. | To present detailed tabulated information on deaths in custody in Australia for the year 2023-24 and to provide jurisdictional breakdowns for comparative purposes. | Data collected by the NDICP for the year 2023-24 are summarised and presented in tables and figures. | N/A |
| Commonwealth National Prevention Mechanism 2024 [169] | Post visit summary: Christmas Island Police Station | External Australian Territory | N/A | Government Agency Report | N/A | To report on an inspection of Christmas Island Police Station undertaken by OPCAT monitors in August 2023. These inspection are completed to monitor the treatment of people in detention, the conditions of their detention and identify any systemic issues where there is a risk of torture or ill-treatment. | Inspection of the police cells were undertaken on two occasions, and the service was reviewed over five domains: safety, respect, purposeful activity, wellbeing, and physical and mental health. | This report makes 16 recommendations across the equipment, operation of the police cells. |
| Commonwealth National Preventative Mechanism 2024 [170] | Post Visit Summary: Cocos (Keeling) Islands Police Station | External Australian Territory | N/A | Government Agency Report | N/A | To report on an inspection of Cocos (Keeling) Island Police Station undertaken by three OPCAT monitors in August 2023. These inspection are completed to monitor the treatment of people in detention, the conditions of their detention and identify any systemic issues where there is a risk of torture or ill-treatment. | Cocos Island Police Station was visited and inspected, and the service was reviewed over five domains: safety, respect, purposeful activity, wellbeing, and physical and mental health. | The report makes 17 recommendations across detainee monitoring, custodial communication systems, the provision of exercise space, and upgrades to cell design. |
| Commonwealth National Preventive Mechanism 2024 [171] | Post Visit Summaries: ACT Policing Watch House and ACT Police Stations | ACT | N/A | Government Agency Report | 3 males were in detention at the time of the inspection. | To report on the inspections of the ACT Policing Watch House in June 2023, and ACT Police Stations in April and May 2023 conducted by the ACT Ombudsman and Commonwealth NPM team. These inspection are completed to monitor the treatment of people in detention, the conditions of their detention and identify any systemic issues where there is a risk of torture or ill-treatment. | The ACT Policing Watch House and four police stations (Belconnen, Gungahlin, Tuggeranong, and Woden) were inspected. The services were reviewed over five domains: safety, respect, purposeful activity, wellbeing, and physical and mental health. | Eleven suggestions were made relevant to the police stations and 21 suggestions were made to the ACT Policing Watch House. |
| Commonwealth National Preventive Mechanism 2024 [172] | Post Visit Summary: Jervis Bay Police Station | ACT | N/A | Government Agency Report | N/A | To report on the inspection of the Jervis Bay Police Station in December 2023 conducted by the ACT Ombudsman and Commonwealth NPM team. This inspection was completed to monitor the treatment of people in detention, the conditions of their detention and identify any systemic issues where there is a risk of torture or ill-treatment. | The Jervis Bay Police Station was inspected. The services were reviewed over five domains: safety, respect, purposeful activity, wellbeing, and physical and mental health. | The report makes 15 recommendations across fire safety, staff communication, detainee privacy, ligature points, and detainee transport. |

Acronyms: AIC: Australian Institute of Criminology. ACT: Australian Capital Territory. CCTV: Closed-Circuit Television. DUCO: Drug Use Careers of Offenders. DUMA: Drug Use Monitoring in Australia. GHSQ: General Help-Seeking Questionnaire. IBAC: Independent Broad-based Anti-corruption Commission. MDMA: 3,4-methylenedioxymethamphetamine. MCC: Melbourne Custody Centre. MINI: Mini International Neuropsychiatric Interview. NDICP: National Deaths in Custody Program. NT: Northern Territory. NSW: New South Wales. OPCAT: Optional Protocol to the Convention against Torture and Other Cruel, Inhuman or Degrading Treatment or Punishment. OPI: Office of Police Integrity. PC: Police custody. QLD: Queensland. QPS: Queensland Police Service. RCIADIC: Royal Commission into Aboriginal Deaths in Custody. RCQ-D tool: Dutch Readiness to Change Questionnaire. SA: South Australia. TAS: Tasmania. VIC: Victoria. WA: Western Australia.

## **References**

1. Petschel K, Gall JA. A profile of deaths in custody in Victoria, 1991-96. J Clin Forensic Med. 2000;7(2):82-7. doi: <https://dx.doi.org/10.1054/jcfm.2000.0370>.

2. Makkai T. Patterns of recent drug use among a sample of Australian detainees. Addiction. 2001;96(12):1799-808. doi: <https://dx.doi.org/10.1046/j.1360-0443.2001.961217999.x>.

3. Heffernan EB, Finn J, Saunders JB, Byrne G. Substance-use disorders and psychological distress among police arrestees. Med J Aust. 2003;179(8):408-11. doi: <https://dx.doi.org/10.5694/j.1326-5377.2003.tb05617.x>.

4. Taylor B, Brownstein HH, Parry C, Plüddemann A, Makkai T, Bennett T, et al. Monitoring the Use of Illicit Drugs in Four Countries Through the International Arrestee Drug Abuse Monitoring (I-Adam) Program. Criminal Justice. 2003;3(3):269-86. doi: 10.1177/14668025030033003.

5. Brewer R, Dalton D. Assessing the Importance of Culture in Explaining Drug Use amongst Indigenous Police Detainees in Adelaide. Curr Issues Crim Justice. 2008;20(2):265-86. doi: 10.1080/10345329.2008.12035808.

6. Griffiths P, McGregor C, Kalic R, Gately N. Sex differences in drug use and offending at the East Perth Watch House. Drug Alcohol Rev. 2009;28:A25.

7. Baksheev GN, Thomas SDM, Ogloff JRP. Psychiatric disorders and unmet needs in Australian police cells. Aust N Z J Psychiatry. 2010;44(11):1043-51. doi: <https://dx.doi.org/10.1080/00048674.2010.503650>.

8. Sturgiss EA, Parekh V. The work of forensic physicians with police detainees in the Canberra City Watchhouse. J Forensic Leg Med. 2011;18(2):57-61. Epub 20101216. doi: 10.1016/j.jflm.2010.11.014. PubMed PMID: 21315298.

9. Ogloff J, Warren L, Tye C, Blaher F, Thomas S. Psychiatric symptoms and histories among people detained in police cells. Soc Psychiatry Psychiatr Epidemiol. 2011;46(9):871-80. doi: <https://dx.doi.org/10.1007/s00127-010-0256-5>.

10. Baksheev GN, Ogloff J, Thomas S. Identification of mental illness in police cells: A comparison of police processes, the Brief Jail Mental Health Screen and the Jail Screening Assessment Tool. Psychol Crime Law. 2011;18(6):529-42. doi: <https://dx.doi.org/10.1080/1068316X.2010.510118>.

11. Baksheev GN, Warren LJ, Ogloff JRP, Thomas SDM. Correlates of criminal victimisation among police cell detainees in Victoria, Australia. Police Pract Res. 2012;14(6):522-34. doi: <https://dx.doi.org/10.1080/15614263.2012.670032>.

12. Baksheev GN, Thomas SDM, Ogloff JRP. Psychopathology in police custody: The role of importation, deprivation and interaction models. Int J Forensic Ment Health. 2012;11(1):24-32. doi: <https://dx.doi.org/10.1080/14999013.2012.667512>.

13. Marchetti E. Victims or offenders: Who were the 11 Indigenous female prisoners who died in custody and were investigated by the Australian Royal Commission into Aboriginal Deaths in Custody? Int Rev Vict. 2013;19(1):37-49. doi: <https://dx.doi.org/10.1177/0269758012447218>.

14. Swan AC, Goodman-Delahunty J. The relationship between drug use and crime among police detainees: Does gender matter? Int J Forensic Ment Health. 2013;12(2):107-15. doi: <https://dx.doi.org/10.1080/14999013.2013.787561>.

15. Porter LE. Indigenous deaths associated with police contact in Australia: Event stages and lessons for prevention. Aust N Z J Criminol. 2013;46(2):178-99. doi: <https://dx.doi.org/10.1177/0004865813483294>.

16. Simpson M. Caught red-eyed and red-handed: An exploration of cannabis use and criminal offending. Sydney (Australia): University of New South Wales; 2013.

17. Trofimovs J, Dowse L. Mental health at the intersections: The impact of complex needs on police contact and custody for Indigenous Australian men. Int J Law Psychiatry. 2014;37(4):390-8. doi: <https://dx.doi.org/10.1016/j.ijlp.2014.02.010>.

18. McArdle DJ, Howie RS, Harle RA. Two cases of benign pneumomediastinum in patients with psychosis who had been restrained in police custody. Aust N Z J Psychiatry. 2017;51(4):412-3. doi: <https://dx.doi.org/10.1177/0004867416659367>.

19. Crissman B. Deaths of people with serious mental disorder: An exploration of deaths in custody and fatal police contacts. Austr J Soc Iss. 2019;54(3):245-66. doi: 10.1002/ajs4.70.

20. Walsh T, Counter A. Deaths in custody in Australia: A quantitative analysis of coroners’ reports. Curr Issues Crim Justice. 2019;31(2):143-63. doi: 10.1080/10345329.2019.1603831.

21. Walker S, Higgs P, Stoove M, Wilson M. "They just don't care about us!": Police custody experiences for young men with histories of injecting drug use. Aust N Z J Criminol. 2020;53(1):102-20. doi: <https://dx.doi.org/10.1177/0004865819868004>.

22. Walker S, Higgs P, Stoove M, Wilson M. "That's the Lowest Place on Earth!" Experiences of the carceral spaces of Australian police custody for marginalized young men. Qual Health Res. 2020;30(6):880-93. doi: <https://dx.doi.org/10.1177/1049732319897603>.

23. Payne JL, Langfield CT. How risky are heroin markets? A multi-site study of self-reported risk perceptions among police detainees in Australia. Int J Drug Policy. 2020;90(9014759):103062. doi: <https://dx.doi.org/10.1016/j.drugpo.2020.103062>.

24. Payne JL, Langfield CT. When two measures of drug dependency do not accord: prevalence, correlates, and implications for treatment in the criminal justice context : A preprint. Open Science Framework. 2020. doi: <https://osf.io/preprints/socarxiv/tz2ax_v1>.

25. Langfield CT, Payne JL. What factors predict the self-identification of drug dependency among Australian police detainees? Prevalence, correlates, and implications for the criminal justice system. J Drug Issues. 2021;51(1):3-22. doi: 10.1177/0022042620952768.

26. Langfield CT, Payne JL. ‘I am drug dependent’: A study of self-identification and prior criminal justice contact using archival data from the Drug Use Monitoring in Australia (DUMA) program. Curr Issues Crim Justice. 2021;34(1):95-111. doi: 10.1080/10345329.2021.1973655.

27. Hine KA, Payne JL, Piquero AR. When suspects resist arrest: Prevalence, correlates, and implications for front-line policing. Police Q. 2021;24(2):135-58. doi: <https://dx.doi.org/10.1177/1098611120957767>.

28. Walsh T. Women who die in custody: What Australian coroners’ reports tell us. Howard J Crime Justice. 2022;61(4):540-55. doi: 10.1111/hojo.12495.

29. Chidgey K, Procter N, Baker A, Grech C. Suicide deaths following police contact: A review of coronial inquest findings. Death Stud. 2022;46(3):675-83. Epub 20200502. doi: 10.1080/07481187.2020.1758243. PubMed PMID: 32362242.

30. van de Ven K, Zahnow R, Livingston M. Australian police detainees who use anabolic-androgenic steroids (AAS) and their involvement in violent crimes compared to detainees using substances other than AAS. Perform Enhancement Health. 2023;11(1). doi: 10.1016/j.peh.2022.100242.

31. Gately N, Rock S, Finney JL, Parry C. Complex lives and procedural barriers: Detainees’ “life happens” explanations for breaching orders. J Criminology. 2024;58(2):299-320. doi: 10.1177/26338076241265980.

32. Dalton V. Australian deaths in custody and custody-related police operations 1999. Canberra (Australia): Australian Institute of Criminology; 2000 Jun.

33. Makkai T, Johnson D, Loxley W. Patterns of drug use amongst police detainees: 1999-2000. Canberra (Australia): Australian Institute of Criminology; 2000.

34. Makkai T, Fitzgerald J, Doak P. Drug use among police detainees. Sydney (Australia): NSW Bureau of Crime Statistics and Research; 2000 Mar.

35. Makkai T. Drug Use Monitoring in Australia (DUMA): 1999 annual report on drug use among adult detainees. Canberra (Australia): Australian Institute of Criminology; 2000.

36. Makkai T. Drug transactions: some results from the DUMA project. Australian Illicit Drug Report 1998-99. Canberra (Australia): Commonwealth of Australia; 2000.

37. Loxley WM, Lien D. Drug use monitoring in Australia: Western Australian 2000 report on drug use among police detainees. Perth (Australia): National Drug Research Institute; 2001 Oct.

38. Collins L, Mouzos J. Australian deaths in custody and custody-related police operations, 2000. Canberra (Australia): Australian Institute of Criminology; 2001.

39. Williams P. Deaths in custody: 10 years on from the Royal Commission. Canberra (Australia): Australian Institute of Criminology; 2001 Apr.

40. Makkai T, McGregor K. Drug use monitoring in Australia (DUMA): 2000 annual report on drug use among police detainees. Canberra (Australia): Australian Institute of Criminology; 2001.

41. Victorian Ombudsman. Report on conditions and overcrowding in police cells. Melbourne (Australia): Victorian Ombudsman; 2002 May.

42. Drug use monitoring of police detainees in New South Wales: The first two years. Sydney (Australia): NSW Bureau of Crime Statistics and Research; 2002 Sep.

43. Poyser C, Makkai T, Norman L, Mills L. Drug driving among police detainees in three states of Australia: Final report. Canberra (Australia): Commonwealth Department of Health and Ageing; 2002 Aug.

44. Weierter S, Lynch M. Drug use and crime: findings from the DUMA survey. Brisbane (Australia): Crime and Misconduct Commission; 2002.

45. Collins L, Mouzos J. Deaths in custody: A gender-specific analysis. Canberra (Australia): Australian Institute of Criminology; 2002 Sep.

46. Collins L. Deaths in custody in Australia: 2001 National Deaths in Custody Program (NDICP) annual report. Canberra (Australia): Australian Institute of Criminology; 2002.

47. Makkai T, McGregor K. Drug Use Monitoring in Australia (DUMA): 2001 annual report on drug use among police detainees. Canberra (Australia): Australian Institute of Criminology; 2002.

48. McGregor K, Makkai T. Self-reported drug use: How prevalent is under-reporting? Canberra (Australia): Australian Institute of Criminology; 2003 Jun.

49. Collins L, Ali M. Deaths in custody in Australia: 2002 National Deaths In Custody Program (NDICP) annual report. Canberra (Australia): Australian Institute of Criminology; 2003.

50. Wei Z, Makkai T, McGregor K. Drug use among a sample of juvenile detainees. Canberra (Australia): Australian Institute of Criminology; 2003 Jun.

51. Makkai T, McGregor K. Drug Use Monitoring in Australia (DUMA): 2002 annual report on drug use among police detainees. Canberra (Australia): Australian Institute of Criminology; 2003.

52. Milner L, Mouzos J, Makkai T. Drug Use Monitoring in Australia: 2003 annual report on drug use among police detainees. Canberra (Australia): Australian Institute of Criminology; 2004.

53. McCall M. Deaths in custody in Australia: 2003 National Deaths in Custody Program (NDICP) annual report. Canberra (Australia): Australian Institute of Criminology; 2004.

54. Milner L, McGregor K. Cocaine use among a sample of police detainees. Canberra (Australia): Australian Institute of Criminology; 2004 May.

55. Sallybanks J. Monitoring injuries in police custody: A feasibility and utility study. Canberra (Australia): Australian Institute of Criminology, 2005.

56. Ombudsman ACT. Annual report 2004 - 2005. Canberra (Australia): Australian Capital Territory Ombudsman, 2005.

57. Taylor N, Bareja M. 2002 national police custody survey. Canberra (Australia): Australian Institute of Criminology; 2005.

58. Putt J, Payne J, Milner L. Indigenous male offending and substance abuse. Canberra (Australia): Australian Institute of Criminology; 2005 Feb.

59. Schulte C, Mouzos J, Makkai T. Drug use monitoring in Australia: 2004 annual report on drug use among police detainees. Canberra (Australia): Australian Institute of Criminology; 2005.

60. Joudo J, Veld M. Deaths in custody in Australia: National Deaths in Custody Program annual report 2004. Canberra (Australia): Australian Institute of Criminology; 2005.

61. Office of the Inspector of Custodial Services. Directed review of the management of offenders in custody. Perth (Australia): Government of Western Australia; 2005 Nov.

62. Ziersch E, Turner N. Drug use and vehicle crime: an analysis of DUMA data on offenders arrested for motor vehicle theft. Adelaide (Australia): National Comprehensive Auto-theft Research System; 2005 Dec.

63. Ombudsman Victoria & Office of Police Integrity Victoria. Conditions for persons in custody: Report of Ombudsman Victoria and Office of Police Integrity. Melbourne (Australia): Victorian Ombudsman; 2006 Jul.

64. Mouzos J, Smith L, Hind N. Drug use monitoring in Australia: 2005 annual report on drug use among police detainees. Canberra (Australia): Australian Institute of Criminology; 2006.

65. Joudo J. Deaths in custody in Australia: National Deaths in Custody Program annual report 2005. Canberra (Australia): Australian Institute of Criminology; 2006.

66. Joudo J. Deaths in custody in Australia 1990-2004. Canberra (Australia): Australian Institute of Criminology; 2006 Apr.

67. Australian Federal Police & Commonwealth Ombudsman. Review of ACT Policing’s watchhouse operations: A joint report by the Australian Federal Police and the Commonwealth Ombudsman. Canberra (Australia): Commonwealth of Australia; 2007 Jun.

68. Ombudsman Victoria. Investigation into the use of excessive force at the Melbourne Custody Centre Melbourne (Australia): Ombudsman Victoria; 2007 Nov.

69. Mouzos J, Smith L. Partner violence among a sample of police detainees. Canberra (Australia): Australian Institute of Criminology; 2007.

70. Mouzos J, Hind N, Smith L, Adams K. Drug use monitoring in Australia: 2006 annual report on drug use among police detainees. Canberra (Australia): Australian Institute of Criminology; 2007.

71. Loxley W. Benzodiazepine use and harms among police detainees in Australia. Canberra (Australia): Australian Institute of Criminology; 2007 May.

72. ACT Human Rights Commission. Human Rights Audit on the operation of ACT Correctional Facilities under Corrections legislation. Canberra (Australia): Australian Institute of Criminology; 2007 Jul.

73. Crime and Misconduct Commission Queensland. Drugs and crime: Trends among watch-house detainees. Brisbane (Australia): Crime and Misconduct Commission; 2008 Mar.

74. Adams K, Sandy L, Smith L, Triglone B. Drug use monitoring in Australia: 2007 annual report on drug use among police detainees. Canberra (Australia): Australian Institute of Criminology; 2008.

75. Adams K, Smith L, Hind N. Drug driving among police detainees in Australia. Canberra (Australia): Australian Institute of Criminology; 2008 Jun.

76. Joudo J, Curnow J. Deaths in custody in Australia: National Deaths in Custody Program annual report 2006. Canberra (Australia): Australian Institute of Criminology; 2008.

77. Crime and Misconduct Commission Queensland. Restoring order: Crime prevention, policing and local justice in Queensland’s Indigenous communities. Brisbane (Australia): Crime and Misconduct Commission; 2009 Nov.

78. Forsythe L, Adams K. Mental health, abuse, drug use and crime: does gender matter? Canberra (Australia): Australian Institute of Criminology; 2009 Nov.

79. Loxley W, Adams K. Women, drug use and crime: findings from the Drug Use Monitoring in Australia program. Canberra (Australia): Australian Institute of Criminology; 2009.

80. Curnow J, Joudo Larsen J. Deaths in custody in Australia: National Deaths in Custody Program 2007. Canberra (Australia): Australian Institute of Criminology; 2009.

81. Office of Police Integrity [Victoria]. Update on conditions in Victoria Police cells. Melbourne (Australia): Office of Police Integrity; 2010 Jun.

82. Gaffney A, Jones W, Sweeney J, Payne J. Drug use monitoring in Australia: 2008 annual report on drug use among police detainees. Canberra (Australia): Australian Institute of Criminology; 2010.

83. Lyneham M, Joudo Larsen J, Beacroft L. Deaths in custody in Australia: National Deaths in Custody Program 2008. Canberra (Australia): Australian Institute of Criminology; 2010.

84. Office of the Inspector of Custodial Services. Thematic review of court security and custodial services in Western Australia. Perth (Australia): Government of Western Australia; 2010 May.

85. Gately N, Fleming J, Morris R, McGregor C. Amphetamine use among detainees at the East Perth Watch House: What is the impact on crime? Perth (Australia): School of Law & Justice, Edith Cowan University; 2011 Apr.

86. McGregor C, Gately N, Fleming J. Prescription drug use among detainees: Prevalence, sources and links to crime. Canberra (Australia): Australian Institute of Criminology; 2011 Aug.

87. Sweeney J, Payne J. Poly drug use among police detainees. Canberra (Australia): Australian Institute of Criminology; 2011 Aug.

88. Ness A, Payne J. Patterns of mephedrone, GHB, Ketamine and Rohypnol use among police detainees: Findings from the DUMA program. Canberra (Australia): Australian Institute of Criminology; 2011 May.

89. The Parliament of the Commonwealth of Australia. Doing time - time for doing: Indigenous youth in the criminal justice system Canberra (Australia): Commonwealth of Australia; 2011 Jun.

90. Macgregor S, Payne J. Increase in use of methamphetamine. Canberra (Australia): Australian Institute of Criminology; 2011 Nov.

91. Sweeney J, Payne J. Alcohol and assault on Friday and Saturday nights: Findings from the DUMA program. Canberra (Australia): Australian Institute of Criminology; 2011 May.

92. Sweeney J, Payne J. Alcohol and disorderly conduct on Friday and Saturday nights: Findings from the DUMA program. Canberra (Australia): Australian Institute of Criminology; 2011 May.

93. Sweeney J, Payne J. Victimisation and fear of crime among a sample of police detainees: Findings from the DUMA program. Canberra (Australia): Australian Institute of Criminology; 2011 Jun.

94. Payne J, Gaffney A. How much crime is drug or alcohol related? Self-reported attributions of police detainees. Canberra (Australia): Australian Institute of Criminology; 2012 May.

95. Forsythe L, Gaffney A. Mental disorder prevalence at the gateway to the criminal justice system. Canberra (Australia): Australian Institute of Criminology; 2012 Jul.

96. Sweeney J, Payne J. “Initiation into drug use” addendum: Findings from the DUMA program. Canberra (Australia): Australian Institute of Criminology; 2012 Aug.

97. Macgregor S, Payne J. Cannabis use and mental health: Findings from a sample of offenders in police custody [Internet] 2012 [cited 2025 Jun 5]. Available from: <https://cannabissupport.com.au/cannabis-use-and-mental-health-findings-from-a-sample-of-offenders-in-police-custody/>.

98. Sweeney J, Macgregor S. Decrease in use of ecstasy/MDMA. Canberra (Australia): Australian Institute of Criminology; 2012 Feb.

99. Sweeney J, Payne J. Drug use monitoring in Australia: 2009-10 report on drug use among police detainees. Canberra (Australia): Australian Institute of Criminology; 2012.

100. Bradford D, Payne J. Illicit drug use and property offending among police detainees. Sydney (Australia): NSW Bureau of Crime Statistics and Research; 2012 Jan.

101. Ng S, Macgregor S. Pharmaceutical drug use among police detainees. Canberra (Australia): Australian Institute of Criminology; 2012.

102. Forsythe L. Measuring mental health in criminology research: Lessons from the Drug Use Monitoring in Australia program. Canberra (Australia): Australian Institute of Criminology; 2013.

103. Lyneham M, Chan A. Deaths in custody in Australia to 30 June 2011: Twenty years of monitoring by the National Deaths in Custody Program since the Royal Commission into Aboriginal Deaths in Custody. Canberra (Australia): Australian Institute of Criminology; 2013.

104. Community Development and Justice Standing Committee (Parliament of Western Australia). In safe custody: Inquiry into custodial arrangements in police lock-ups. Perth (Australia): Parliament of Western Australia; 2013 Nov.

105. Payne J, Sweeney J, Macgregor S. Attitudes and perceptions towards drug driving amongst a sample of cannabis using police detainees [Internet]: Cannabis Information & Support; 2013 [cited 2025 June 4]. Available from: <https://cannabissupport.com.au/ing-police-detainees/>.

106. Victorian Ombudsman. Investigation into deaths and harm in custody. Melbourne (Australia): Victorian Ombudsman; 2014 Mar.

107. Doyle J, Lonsdale M, Pitt S-A, Anismova A, Sheard C. Prisoner transportation. Melbourne (Australia): Victorian Auditor-General’s Office; 2014 Jun.

108. Coghlan S, Goldsmid S. Findings from the DUMA program: Methamphetamine drug market trends. Canberra (Australia): Australian Institute of Criminology; 2015 Sep.

109. Ng S, Gannoni A, Coghlan S, Goldsmid S. Drug use monitoring in Australia: 2011-12 report on drug use among police detainees. Canberra (Australia): Australian Institute of Criminology; 2015.

110. Gannoni A, Goldsmid S, Patterson E. Methamphetamine in Brisbane: Perspectives from DUMA police detainees. Canberra (Australia): Australian Institute of Criminology; 2015 Dec.

111. Baker A, Cussen T. Deaths in custody in Australia: National Deaths in Custody Program 2011-12 and 2012-13. Canberra (Australia): Australian Institute of Criminology; 2015.

112. Coghlan S, Gannoni A, Goldsmid S, Patterson E, Willis M. Drug use monitoring in Australia: 2013-14 report on drug use among police detainees. Canberra (Australia): Australian Institute of Criminology; 2015.

113. Brown R. Explaining the property crime drop: The offender perspective. Canberra (Australia): Australian Institute of Criminology; 2015 Feb.

114. Coghlan S, Goldsmid S. Findings from the DUMA program: Impact of reduced methamphetamine supply on consumption of illicit drugs and alcohol. Canberra (Australia): Australian Institute of Criminology; 2015 Feb.

115. Goldsmid S, Coghlan S, Patterson E. Findings from the DUMA program: Drink and drug driving among police detainees. Canberra (Australia): Australian Institute of Criminology; 2015 May.

116. Patterson E, Goldsmid S, Gannoni A. Methamphetamine in Perth: Perspectives from DUMA police detainees. Canberra (Australia): Australian Institute of Criminology; 2015 Dec.

117. Kapira M, Goldsmid S, Gannoni A. Methamphetamine in Adelaide: Perspectives from police detainees. Canberra (Australia): Australian Institute of Criminology; 2015 Dec.

118. Payne J, Macgregor S, McDonald H. Homelessness and housing stress among police detainees: Results from the DUMA program. Canberra (Australia): Australian Institute of Criminology; 2015 Feb.

119. Independent Broad-based Anti-Corruption Commission. Operation Ross: An investigation into police conduct in the Ballarat Police Service Area. Melbourne (Australia): State of Victoria; 2016 Nov.

120. Fuller G, Goldsmid S, Brown R. Managing intoxicated offenders: Best practice in responding to individuals affected by drugs and alcohol. Canberra (Australia): National Drug Law Enforcement Research Fund; 2016.

121. Patterson E, Goldsmid S, Gannoni A. Methamphetamine in Sydney: Perspectives from DUMA police detainees. Canberra (Australia): Australian Institute of Criminology; 2016 Mar.

122. Gately N, Ellis S, Morris R. Drug Use Monitoring in Australia (DUMA): An expansion into the Pilbara, Western Australia. Canberra (Australia): National Drug Law Enforcement Research Fund; 2016.

123. Fuller G, Goldsmid S. The nature of risk during interactions between the police and intoxicated offenders. Canberra (Australia): Australian Institute of Criminology; 2016 Dec.

124. Goldsmid S, Willis M. Methamphetamine use and acquisitve crime: Evidence of a relatonship. Canberra (Australia): Australian Institute of Criminology; 2016 Oct.

125. The State of Queensland (Crime and Corruption Commission). Police use of force in Queensland watch-houses. Brisbane (Australia): Crime and Corruption Commission; 2017 Dec.

126. White M, Gooda M. Chapter 25: The path into detention. Royal Commission into the protection and detention of children in the Northern Territory. Volume 2B. Canberra (Australia): Commonwealth of Australia; 2017 Nov.

127. Gannoni A, Goldsmid S. Readiness to change drug use and help-seeking intentions of police detainees: Findings from the DUMA program. Canberra (Australia): Australian Institute of Criminology; 2017 Jan.

128. Patterson E, Sullivan T. The cannabis market : Police detainee perspectives. Canberra (Australia): Australian Institute of Criminology; 2018 Apr.

129. Sullivan T, Patterson E. The ecstasy market: Police detainee perspectives. Canberra (Australia): Australian Institute of Criminology; 2018 Apr.

130. Ticehurst A, Sullivan T. The methamphetamine market: Police detainee perspectives. Canberra (Australia): Australian Institute of Criminology; 2018 Apr.

131. Inspector of Custodial Services. Inspection of 24-hour court cells in NSW. Sydney (Australia): State of New South Wales; 2018 Jun.

132. Sullivan T, Ticehurst A, Bricknell S. Prescription opioid use among Australian police detainees. Canberra (Australia): Australian Institute of Criminology; 2018 Apr.

133. Patterson E, Sullivan T, Ticehurst A. Use and misuse of prescription drugs among police detainees. Canberra (Australia): Australian Institute of Criminology; 2018 Apr.

134. Patterson E, Sullivan T. The heroin market: Police detainee perspectives. Canberra (Australia): Australian Institute of Criminology; 2018 Apr.

135. Ticehurst A, Napier S, Bricknell S. National Deaths in Custody Program: Deaths in custody in Australia 2013-14 and 2014-15. Canberra (Australia): Australian Institute of Criminology; 2018.

136. Sullivan T, Patterson E. Fentanyl use by police detainees remains unchanged. Canberra (Australia): Australian Institute of Criminology; 2018 Sep.

137. Patterson E, Sullivan T, Bricknell S. Polydrug use among police detainees. Canberra (Australia): Australian Institute of Criminology; 2018 Dec.

138. Patterson E, Sullivan T, Ticehurst A, Bricknell S. Drug use monitoring in Australia: 2015 and 2016 report on drug use among police detainees. Canberra (Australia): Australian Institute of Criminology; 2018.

139. Goldsmid S. Findings from the DUMA program: The influence of cannabis dependency and use on criminal offending, through the eyes of police detainees [Internet]: Cannabis Information & Support; 2015 [cited 2025 3 Feb]. Available from: <https://cannabissupport.com.au/the-influence-of-cannabis-dependency-and-use-on-criminal-offending/>.

140. Gannoni A, Bricknell S. National Deaths in Custody Program: Deaths in custody in Australia 2015-16. Canberra (Australia): Australian Institute of Criminology; 2019.

141. Gannoni A, Bricknell S. National Deaths in Custody Program: Deaths in custody in Australia 2016-17. Canberra (Australia): Australian Institute of Criminology; 2019.

142. Gannoni A, Bricknell S. Indigenous deaths in custody: 25 years since the Royal Commission into Aboriginal deaths in custody. Canberra (Australia): Australian Institute of Criminology; 2019 Feb.

143. Sullivan T, English F. Is alcohol and energy drink consumption associated with antisocial behaviour? Canberra (Australia): Australian Institute of Criminology; 2019 Mar.

144. Patterson E, Sullivan T, Bricknell S. Drug use monitoring in Australia: Drug use among police detainees, 2017. Canberra (Australia): Australian Institute of Criminology; 2019.

145. Voce A, Sullivan T. Drug use monitoring in Australia: Drug use among police detainees, 2018. Canberra (Australia): Australian Institute of Criminology; 2019.

146. Goldsmid S. Findings from the DUMA program: Impact of reduced cannabis supply on consumption of illicit drugs and alcohol [Internet]: Cannabis Information & Support; 2019 [cited 2025 3 Feb]. Available from: <https://cannabissupport.com.au/criminal-justice-bulletin/>.

147. Morgan A, Gannoni A. Methamphetamine dependence and domestic violence among police detainees. Canberra (Australia): Australian Institute of Criminology; 2020 Feb.

148. Voce A, Sullivan T. Why Australian police detainees choose to use (or not use) non-prescribed fentanyl. Canberra (Australia): Australian Institute of Criminology; 2020 Jun.

149. Voce A, Sullivan T. Is there fentanyl contamination in the Australian illicit drug market? Canberra (Australia): Australian Institute of Criminology; 2020 Mar.

150. Doherty L, Sullivan T. Drug use monitoring in Australia: Drug use among police detainees, 2019. Canberra (Australia): Australian Institute of Criminology; 2020.

151. Doherty L, Sullivan T. How and where police detainees obtain methamphetamine. Canberra (Australia): Australian Institute of Criminology; 2020 Apr.

152. Doherty L, Bricknell S. Deaths in custody in Australia 2017-18. Canberra (Australia): Australian Institute of Criminology; 2020.

153. Doherty L, Bricknell S. Deaths in custody in Australia 2018-19. Canberra (Australia): Australian Institute of Criminology; 2020.

154. Sullivan T, Voce I. The social supply of pharmaceutical opioids. Canberra (Australia): Australian Institute of Criminology; 2020 Mar.

155. Voce A, Finney J, Gately N, Sullivan T. COVID-19 pandemic constricts methamphetamine supply in Perth. Canberra (Australia): Australian Institute of Criminology; 2020 Sep.

156. Voce A, Sullivan T. Drug use monitoring in Australia: Drug use among police detainees, 2020. Canberra (Australia): Australian Institute of Criminology; 2021.

157. Doherty L, Sullivan T. Deaths in custody in Australia 2019-20. Canberra (Australia): Australian Institute of Criminology; 2021.

158. Doherty L. Deaths in custody in Australia 2020-21. Canberra (Australia): Australian Institute of Criminology, 2021.

159. Doherty L, Sullivan T, Voce A. Impact of the COVID-19 pandemic on cannabis demand and supply in Australia. Canberra (Australia): Australian Institute of Criminology; 2021 Jul.

160. Law Enforcement Conduct Commission. Operation Kimbla: Report to the Parliament pursuant to section 132 Law Enforcement Conduct Commission Act 2016. Sydney (Australia): State of New South Wales; 2022 Apr.

161. Voce A, Sullivan T. Drug use monitoring in Australia: Drug use among police detainees, 2021. Canberra (Australia): Australian Institute of Criminology; 2022.

162. McAlister M, Bricknell S. Deaths in custody in Australia 2021–22. Canberra (Australia): Australian Institute of Criminology, 2022.

163. Mazerolle L, Ransley J, Marchetti E, Crowley L. Independent review into investigations of police-related deaths, and domestic and family violence deaths in Queensland. Brisbane (Australia): The University of Queensland & Griffith University; 2022.

164. Ombudsman NT. Extraordinary restraint: Spit hood & emergency restraint chair use on children in police custody. Darwin (Australia): Ombudsman NT; 2023 Jun.

165. McAlister M, Miles H, Bricknell S. Deaths in custody in Australia 2022–23. Canberra (Australia): Australian Institute of Criminology, 2023.

166. The State of Queensland (Queensland Family and Child Commission). Who’s responsible: Understanding why young people are being held longer in Queensland watch houses. Brisbane (Australia): The State of Queensland; 2023 Nov.

167. Inspector of Detention Services (Queensland Ombudsman). Cairns and Murgon watch‑houses inspection report: Focus on detention of children. Brisbane (Australia): The State of Queensland (Office of the Queensland Ombudsman); 2024 Sep.

168. Miles H, McAlister M, Bricknell S. Deaths in custody in Australia 2023–24. Canberra (Australia): Australian Institute of Criminology; 2024 Dec.

169. Commonwealth Ombudsman & Commonwealth National Preventative Mechanism. Post visit summary: Christmas Island police station. Canberra (Australia): Commonwealth Ombudsman; 2024 Aug.

170. Commonwealth Ombudsman & Commonwealth National Preventative Mechanism. Post visit summary: Cocos (Keeling) Islands police station. Canberra (Australia): Commonwealth Ombudsman; 2024 Aug.

171. ACT Ombudsman & Commonwealth National Preventative Mechanism. Post visit summaries: ACT Policing watch house and ACT police stations. Canberra (Australia): Commonwealth Ombudsman; 2024 Jun.

172. Commonwealth Ombudsman & Commonwealth National Preventative Mechanism. Post visit summary: Jervis Bay police station. Canberra (Australia): Commonwealth Ombudsman; 2024 Oct.
