# Supplementary material for: The determinants of health and health status of individuals in police custody in Australia: A scoping review
Source: PLoS One. 2025 Dec 30;20(12):e0338957. doi: 10.1371/journal.pone.0338957 (PMC12753082; doi:10.1371/journal.pone.0338957)
Supplement: S8 Appendix — A figure is provided for each jurisdiction in Australia showing the number of identified publications each year between 2000 and 2024. (DOCX) [file pone.0338957.s008.docx]

## **S8 Appendix: Publications per year by jurisdictions**
