# Supplementary figures and images for: The determinants of health and health status of individuals in police custody in Australia: A scoping review
Source: PLoS One. 2025 Dec 30;20(12):e0338957. doi: 10.1371/journal.pone.0338957 (PMC12753082; doi:10.1371/journal.pone.0338957)

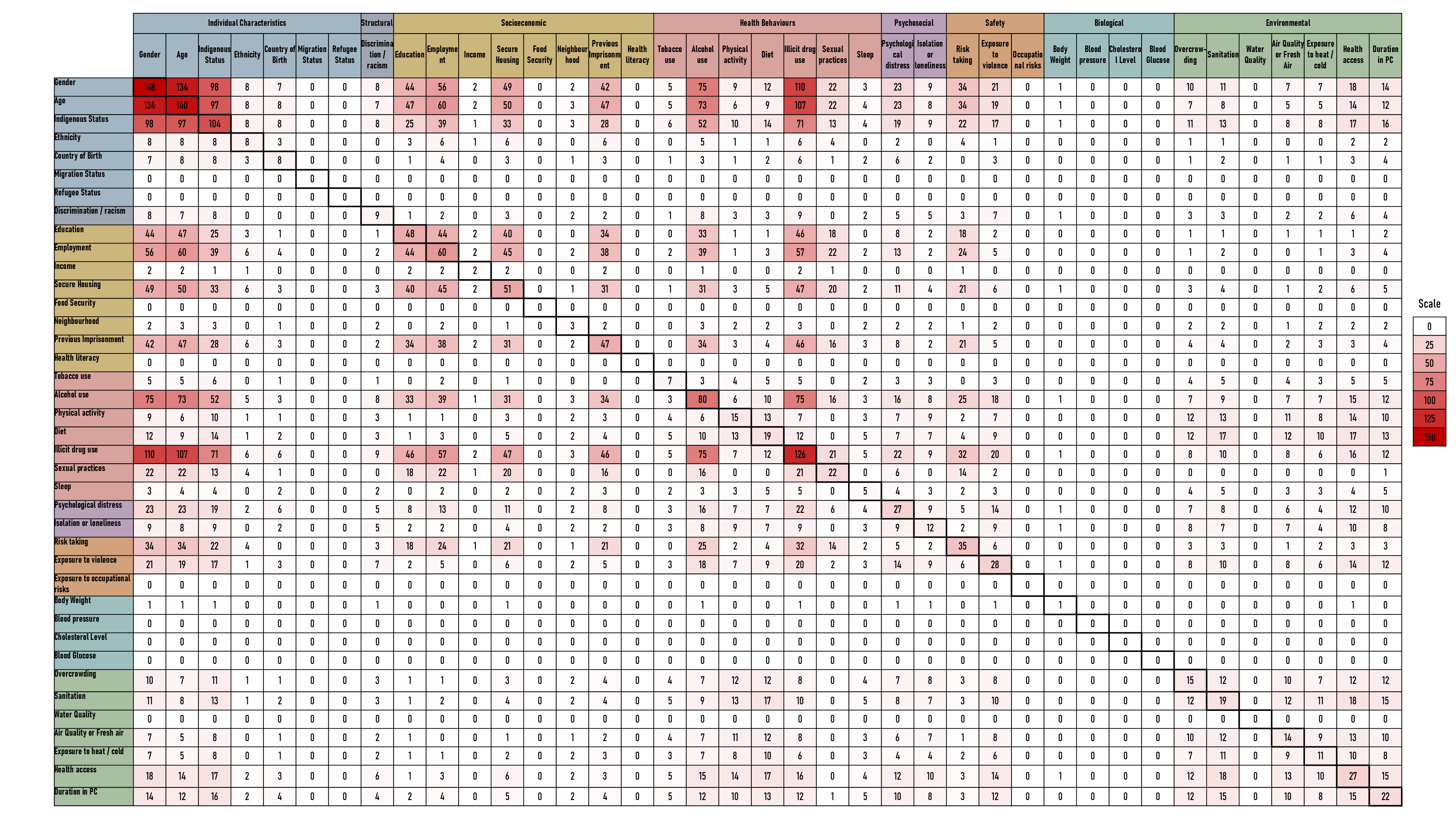

Supplement: S9 Fig — This heatmap visualizes the frequency with which pairs of health determinants co-occurred across all included sources. Each cell represents a unique determinant pairing, with deeper shades of red indicating higher co-occurrence. The diagonal from top left to bottom right represents the frequency of each determinant appearing independently. Blank or pale cells indicate little to no co-occurrence. (TIF) [file pone.0338957.s009.tif]

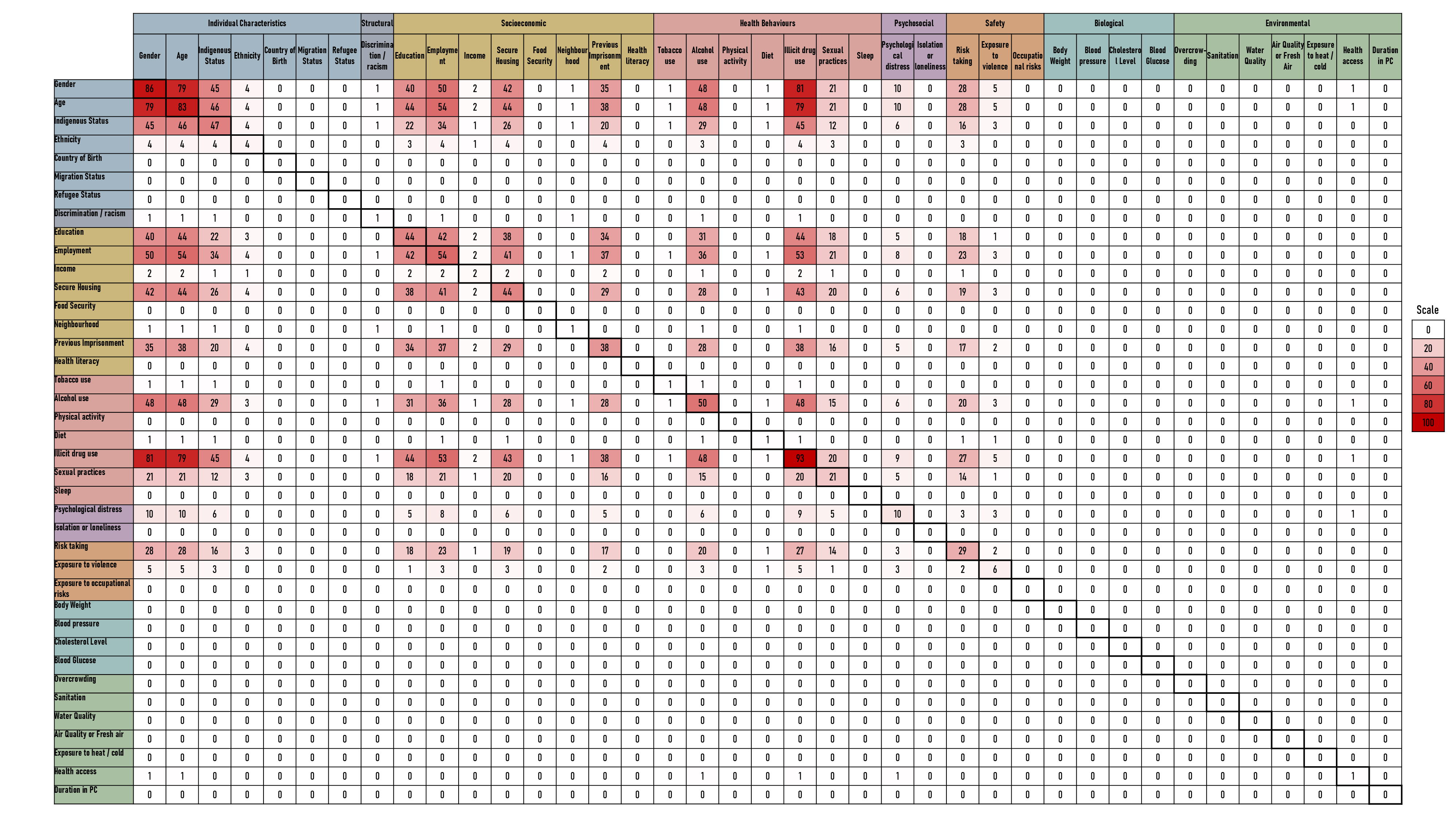

Supplement: S10 Fig — This heatmap visualizes the frequency with which pairs of health determinants co-occurred across sources that utilized data from the Drug Use Monitoring in Australia (DUMA) program. Each cell represents a unique determinant pairing, with deeper shades of red indicating higher co-occurrence. The diagonal from top left to bottom right represents the frequency of each determinant appearing independently. Blank or pale cells indicate little to no co-occurrence. This figure shows that sources utilizing DUMA program data only reported on selected determinants, and did not include any information on others, such as the environmental conditions in police custody. (TIF) [file pone.0338957.s010.tif]

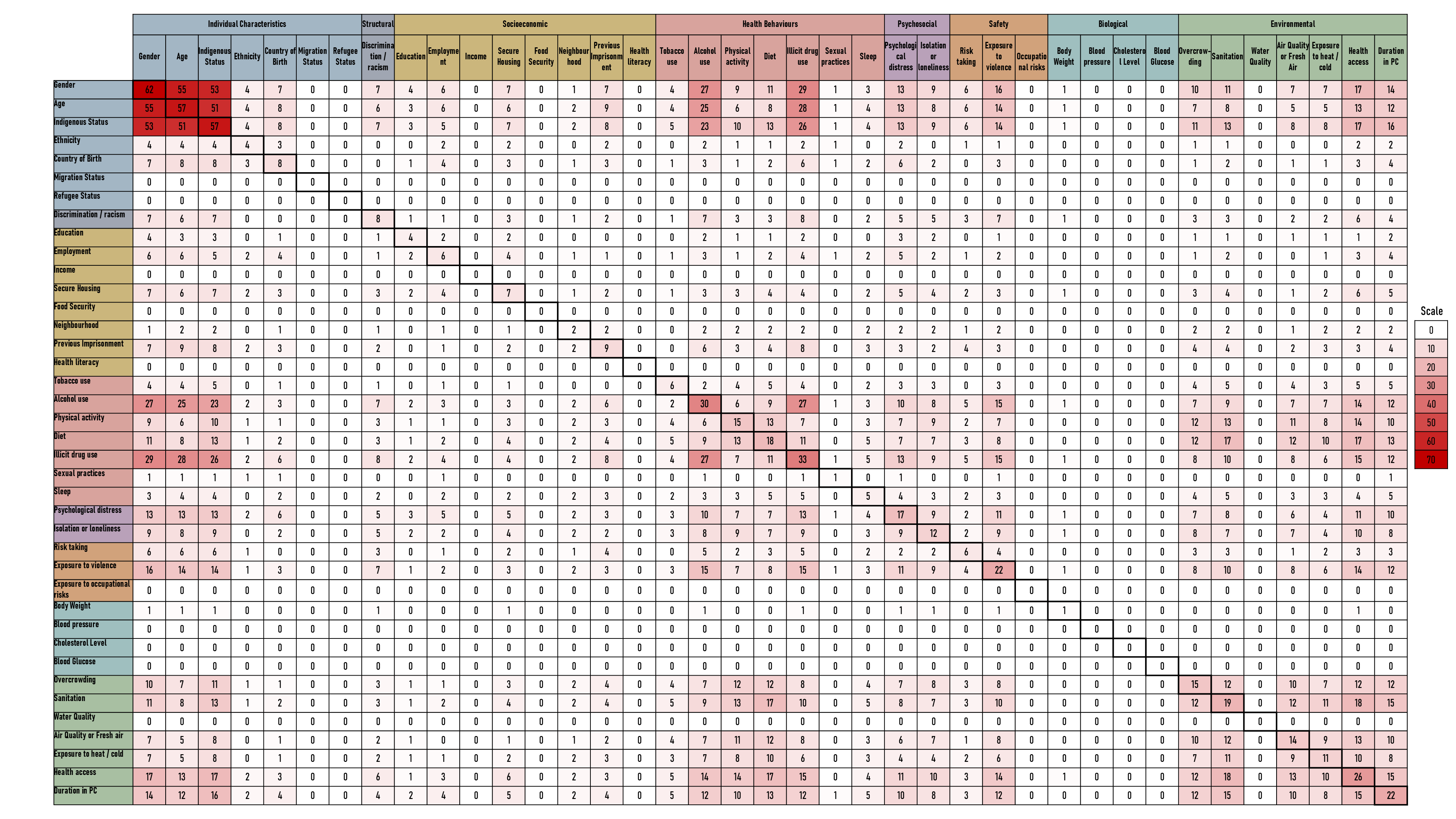

Supplement: S11 Fig — This heatmap visualizes the frequency with which pairs of health determinants co-occurred across sources that did not utilize data from the Drug Use Monitoring Australia (DUMA) program. Each cell represents a unique determinant pairing, with deeper shades of red indicating higher co-occurrence. The diagonal from top left to bottom right represents the frequency of each determinant appearing independently. Blank or pale cells indicate little to no co-occurrence. This figure shows that, outside of the DUMA program, there is some information available on health behavior, safety, and environmental determinants, which were largely absent from DUMA program publications (see S10 Fig). (TIF) [file pone.0338957.s011.tif]
